# Evolution of Somite Compartmentalization: A View From *Xenopus*

**DOI:** 10.3389/fcell.2021.790847

**Published:** 2022-01-17

**Authors:** Bruno Della Gaspera, Laure Weill, Christophe Chanoine

**Affiliations:** Faculté des Sciences Biomédicales et Fondamentales, Université de Paris-UMR INSERM 1124, Paris, France

**Keywords:** somite compartmentalization, *Xenopus*, dermomyotome, sclerotome, endotome, cell potency, evolution, myotome

## Abstract

Somites are transitory metameric structures at the basis of the axial organization of vertebrate musculoskeletal system. During evolution, somites appear in the chordate phylum and compartmentalize mainly into the dermomyotome, the myotome, and the sclerotome in vertebrates. In this review, we summarized the existing literature about somite compartmentalization in *Xenopus* and compared it with other anamniote and amniote vertebrates. We also present and discuss a model that describes the evolutionary history of somite compartmentalization from ancestral chordates to amniote vertebrates. We propose that the ancestral organization of chordate somite, subdivided into a lateral compartment of multipotent somitic cells (MSCs) and a medial primitive myotome, evolves through two major transitions. From ancestral chordates to vertebrates, the cell potency of MSCs may have evolved and gave rise to all new vertebrate compartments, i.e., the dermomyome, its hypaxial region, and the sclerotome. From anamniote to amniote vertebrates, the lateral MSC territory may expand to the whole somite at the expense of primitive myotome and may probably facilitate sclerotome formation. We propose that successive modifications of the cell potency of some type of embryonic progenitors could be one of major processes of the vertebrate evolution.

## 1 Introduction

In amniote vertebrates, somite development has been the subject of intense research over many decades, giving insight into the morphological and molecular processes leading to their formation, compartmentalization, and differentiation (Brand-Saberi and Christ, 2000; Buckingham, 2001; Christ et al., 2007; Applebaum and Kalcheim, 2015; Hirst and Marcelle et al., 2015; Chal and Pourquié, 2017; Tani et al., 2020). In anamniote vertebrates, such as *Xenopus*, which is a standard amphibian model of embryonic development, somite development still remains less explored, whereas much more effort has been made in zebrafish, the teleost counterpart (Keller, 2000; Stickney et al., 2000; Scaal and Wiegreffe, 2006; Sabillo et al., 2016; Keenan and Currie, 2019; Lewandowski et al., 2020).

**FIGURE 1:**
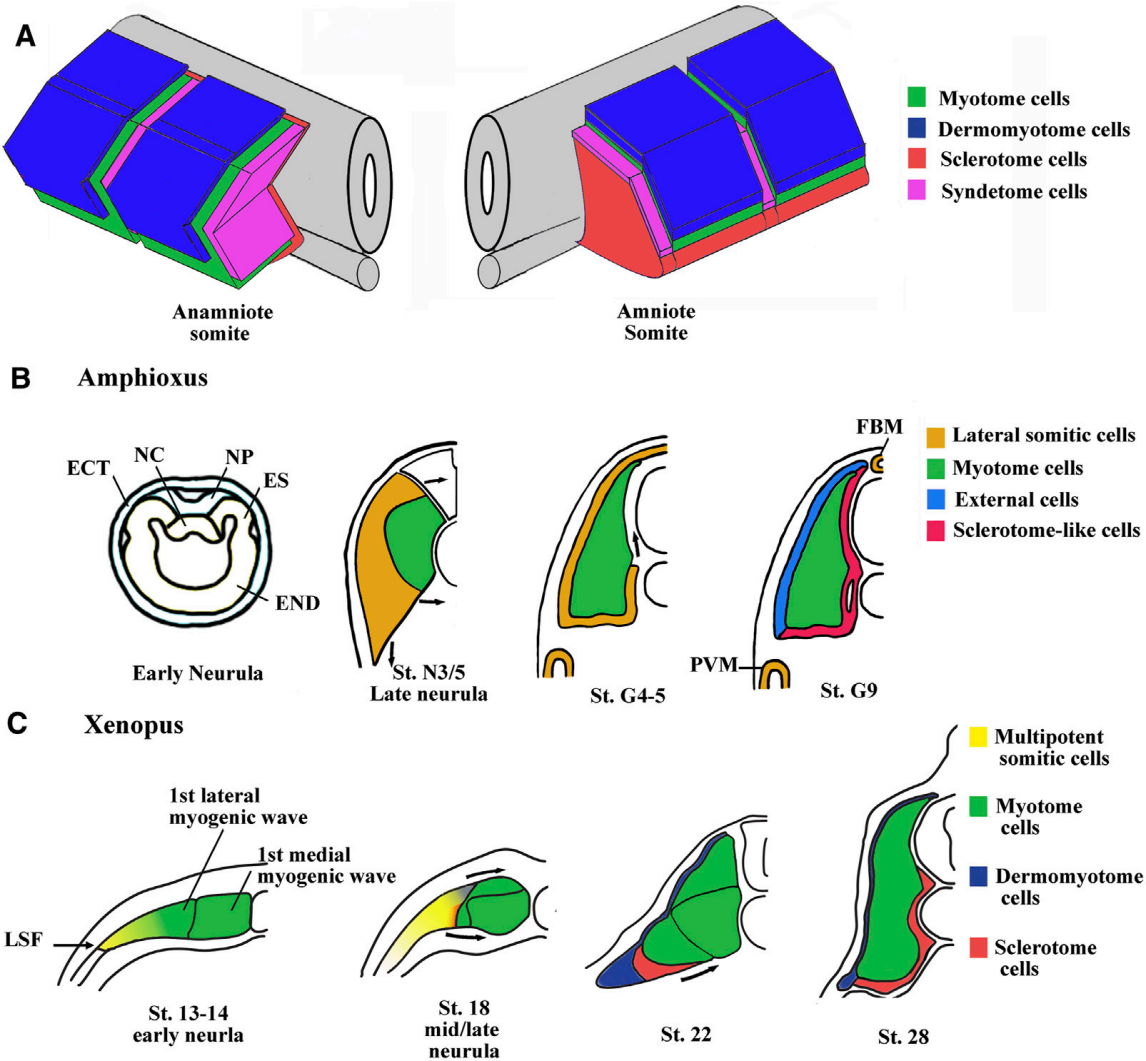
Main characteristics of somite compartmentalization in vertebrates. **(A)** Comparison of somite organization between amniotes and anamniotes. Schematic view of somites slightly after the phylotypic stage. In anamniotes, the somite organization is adapted to the ondulatory swimming of the larvae and harbors a chevron shape with the myotome occupying the majority of the somite. The thin layer of dermomyotome cells is in dorso-lateral position and the sclerotome layer is ventro-medially stranded between the myotome and the midline structures (neural tube and notochord). The syndetome at the origin of the dorsal tendons arises from the sclerotome. The tenocytes project cytoplasmic extensions between muscle cells of adjacent somites. In amniotes, the spatial organization is the same, but the myotome compartment is reduced and the sclerotome is larger. **(B)** Somite compartmentalization in amphioxus. The anterior and intermediate somites are formed by enterocoely from the endoderm at the early neurula stage. The somites are subdivided into a medial myotome and a lateral domain at late neurula stage. The sclerotome-like cells seem to migrate from the lateral domain to position themselves medially between the myotome and the axial structures. The lateral domain also gives rise to the dorsal external cells, the medial fin box mesothelium (FBM) and the latero-ventral perivisceral mesothelium (PVM). Modified from Mansfield et al. (2015) and Yong et al. (2021). ES, epithelial somite; NC, notochord; NP, neural plate; END, endoderm; ECT, ectoderm. **(C)** Somite compartmentalization in *Xenopus*. The somite is initially medio-laterally organized with the myotome in medial position and multipotent somitic cells (MSCs) in lateral one. The myotome forms first and is initially made up of a medial- and a lateral-population of muscle cells. The MSCs appear at lateral somitic Frontier (LSF) at the beginning of neurulation and envelop next dorsally and ventrally the myotome to give rise to both dermomyotome and sclerotome.

Somites are metameric units only found in chordate phylum, located in the dorsal region of the embryo on either side of the notochord and the neural tube. After gastrulation, somites which are formed from the paraxial mesoderm, segment, and differentiate in an antero-posterior direction in close coordination with embryo elongation at its posterior end. In vertebrates, the bilaterally symmetric somite pairs appear at constant intervals, according to a mechanism known as the “clock and wavefront model” leading to the formation of a border separating the posterior cells of the nascent somite from the presomitic mesoderm (Dequéant and Pourquié, 2008; Hubaud and Pourquié, 2014). During their differentiation, somites subdivide into the dermomyotome, the myotome, the sclerotome, and finally the syndetome. These divisions form the basis of the axial organization of musculoskeletal system. For instance, the vertebrae and ribs derive from the sclerotome and dorsal tendons from syndetome while skeletal muscles of the trunk and limbs originate from the dermomyotome and the myotome (Tajbakhsh et al., 1997; Brent et al., 2003; Scaal, 2016). The somite organization at the phylotypic stage (the stage of development with the highest homology in vertebrates) is comparable between amniote and anamniote vertebrates, with a dermomyotome in the dorso-lateral location, a sclerotome in the ventro-medial location, and a myotome separating these two compartments (Figure 1A).

Nevertheless, the initial phase of compartmentalization differs between amniotes and anamniotes. In amniote vertebrates, the newly formed somite is a naïve territory that rapidly compartmentalizes into the dermomyotome and the sclerotome. Subsequently, myotome cells arise from the dermomyotome and position themselves between these two compartments, while the syndetome appears in the sclerotome vicinity of the myotome. In anamniotes, the myotome appears first, before somite formation, and the somites are essentially composed of muscle cells (Weinberg et al., 1996; Hopwood et al., 1992; Stickney et al., 2000; Scaal and Wiegreffe, 2006; Della Gaspera et al., 2012a; Gros et al., 2004). In addition, the initial phase of compartmentalization is mainly dorso-ventrally organized in amniotes with a dorso-lateral dermomyotome and a ventro-medial sclerotome, whereas it seems initially medio-laterally subdivided in anamniotes with a medial myotome and an undifferentiated lateral territory (Devoto et al., 1996; Hinits et al., 2009; Della Gaspera et al., 2012a; Della Gaspera et al., 2012b). Compartmentalization is also medio-laterally organized in cephalochordates, another chordate subphylum, which also possesses somites, suggesting that this compartmentalization scheme was shared by the last common chordate ancestor (Mansfield et al., 2015). The cephalochordate amphioxus somite has a medial myotome but no characteristic dermomyotome or sclerotome (Holland, 1996). Nevertheless, in this species, it has been observed that cells originating from the lateral somitic region migrate under the myotome and creep in medially between the myotome and the notochord to form a sclerotome-like compartment (Mansfield et al., 2015) (Figure 1B). We have also determined that cells from the lateral somitic frontier (LSF) in *Xenopus* give rise to both the sclerotome and the dermomyotome *via* the dorsal and the ventral route around the myotome (Della Gaspera et al., 2019) (Figure 1C). Consequently, we recently proposed a model of somite organization that describes the evolutionary history of their compartmentalization from the last common ancestor of chordates to amniote vertebrates and explains the common lateral origin of the sclerotome and the dermomyotome (Della Gaspera et al., 2019). The ancestral organization of somites in the last chordate ancestor would have been subdivided into a myotome medially positioned and into a compartment composed of multipotent somitic cells (MSCs) laterally positioned. This myotome differentiates first and can be defined as the primitive myotome, and the MSCs differentiate later and give rise to distinct cell types (Figures 1B, C). The compartmentalization of the somite would have undergone two major evolutionary transitions. The first one would have occurred during the transition from the last common ancestor of chordates to that of vertebrates, and allowed the MSCs differentiation capacities to increase in order to give rise to all the new vertebrate compartments, i.e., the dermomyotome itself, its hypaxial region, and the sclerotome. The second transition from anamniotes to amniotes would have driven the expansion of the lateral territory of MSCs to the whole somite at the expense of the primitive myotome leading, in particular, to the formation of a larger sclerotome (Della Gaspera et al., 2012a; Della Gaspera et al., 2019).

In this review, we describe the formation and compartmentalization of somites in *Xenopus* in comparison with other vertebrates. We also highlight signals and transcription factors influencing the development and regionalization of somites. Furthermore, we discuss the arguments in favor of the present model tracing the evolutionary history of the somite, and its potential implication on the formation of muscle-associated tissues.

## 2 Somite Compartmentalization in *Xenopus*

### 2.1 The First Myogenic Wave Gives Rise to the Primitive Myotome

**FIGURE 2:**
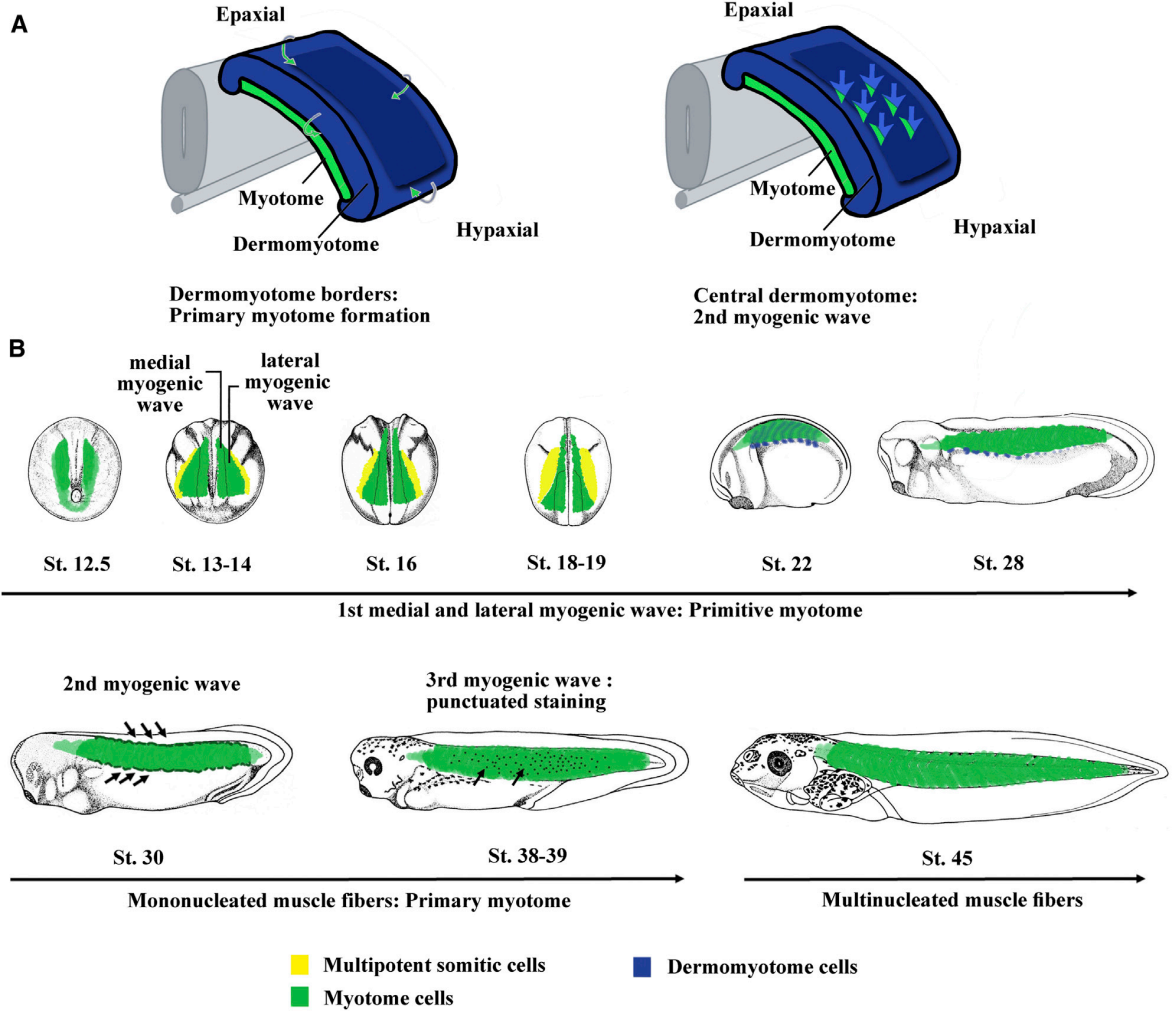
The myogenic waves and the myotome formation in amniotes and *Xenopus*. **(A)** The myotome is essentially derived from the dermomyotome in amniotes. The primary myotome is made of mononucleated cells arising from the four borders of the dermomyotome. Next cells coming from the central dermomyotome invade the primary myotome and contribute to the myotome growth. Hence, two myogenic waves are at the origin of myotome formation. Modified from Lagha et al. (2008a). **(B)** Myotome is the main somite compartment in *Xenopus* and is formed by at least three myogenic waves. The first myogenic wave is made up of two subpopulations, a medial and a lateral one, constitutes the primitive myotome and arises directly from paraxial mesoderm. The second myogenic wave arises from epaxial and hypaxial border of the dermomyotome at stage 28–30. The third myogenic wave has been visualized by myf5 mRNA staining that marked isolated round cells inside the myotome at stage 37–38. The myotome is initially made up of mononucleated fibers until stage 45 when the first multinucleated muscle fibers were observed. Hence, it can be considered that both the first wave of primitive myotome and the second wave of hypaxial and epaxial dermomyotome contribute to the formation of primary myotome. The third could participate to plurinucleated fibers formation and myotome growth. St., stage.

In vertebrates, successive waves of myoblasts contribute in building the skeletal striated muscle tissue (Kahane et al., 1998a and b; Kahane et al., 2001). In amniote somites, two myogenic waves essentially derived from the dermomyotome have been identified (Figure 2A). The first embryonic wave is at the origin of muscle fibers that form the primary myotome. The second wave participates in myotome growth (Gros et al., 2004 and 2005). In *Xenopus* and zebrafish, the first signs of myogenesis appear early, during the blastula/gastrula transition, long before somite formation (Hopwood et al., 1992; Weinberg et al., 1996; Coutelle et al., 2001; Polli and Amaya, 2002). The myogenesis of skeletal muscle is orchestrated in vertebrates by the four bHLH transcription factors of the Myod family (Comai et al., 2014; Hernández-Hernández et al., 2017). They were classified as master genes since the four members are able to convert fibroblasts into skeletal muscle cells (Davis et al., 1987; Braun et al., 1989; Rhodes and Konieczny, 1989).

Based on the expression of the four myogenic regulatory factors (MRFs) of the Myod family (Myod1, Myf5, Myf6, and Myogenin), three myogenic waves have been identified in *Xenopus* (Grimaldi et al., 2004; Della Gaspera et al., 2012a) (Figure 2B). The first wave at the origin of the primitive myotome appears before dermomyotome formation and is composed of two myogenic populations differentiating in different places and times (Figure 2B). Indeed, from stage 9.5, Myf5 and Myod1 start to be expressed in the dorso-lateral marginal zone and initiate medial myogenesis, which gives rise to the first differentiated fibers located near the notochord at the gastrulation/neurulation transition (Gurdon et al., 1984; Hopwood et al., 1989; Frank and Harland, 1991; Hopwood et al., 1991; Scales et al., 1991; Kato and Gurdon, 1993; Fisher et al., 2002; Polli and Amaya, 2002; Della Gaspera et al., 2012a). During gastrulation, Myod1 expressed in the marginal zone also participates in the activation of genes involved in somitogenesis and seems to link myogenesis to somite formation (Maguire et al., 2012). At the beginning of neurulation from stage 13, a hallmark of lateral myogenesis is the strong expression of Myod1 mRNA in the lateral paraxial mesoderm. These myogenic cells differentiate during somitogenesis (Frank and Harland, 1991; Della Gaspera et al., 2012a). The muscle fibers corresponding to these two myogenic populations are distributed separately at tailbud stage. The dorso-lateral cells of the marginal zone (medial myogenesis) remain associated with the notochord in the head and trunk regions, whereas most of the ventral cells (lateral myogenesis) give rise to muscle fibers that envelop the medial ones during neurulation (Krneta-Stankic et al., 2010). The formation of the first muscle fibers presents a peculiarity in *Xenopus* since it is initiated in the head somites (w, x, y, and z) of the preotic region (Chung et al., 1989; Della Gaspera et al., 2012a). These singular somites gradually disappear during the next embryonic phases (Chung et al., 1989). In *Xenopus*, most of the identified genes of the muscle development program are implicated in the formation of the primitive myotome and somites suggesting that both are interconnected. Among them, three genes, Hes6.1 (Hes6), Egr1, and Mef2d act downstream of Fgf signaling, illustrating the important function of Fgf in the formation of the paraxial mesoderm as well as the primitive myotome (Murai et al., 2007; Nentwich et al., 2009; Della Gaspera et al., 2012b). Other transcription factors are also involved in the formation of the primitive myotome in *Xenopus*. Ebf2 and 3 play a role in muscle specification and in some aspects of differentiation (Green and Vetter, 2011). Sox5 enhances indirectly myogenic transcription through transrepression (Della Gaspera et al., 2018). Other factors involved in the RNA metabolism [Rbm24 (Seb4), Trab2, and Mir-206] and cell cycle decision [Cdknx (p27Xic-1)] also promote primitive myotome formation (Vernon and Philpott, 2003; Li et al., 2010; Dichmann et al., 2015; Vergara et al., 2018).

In zebrafish, the expression of Myf5 and Myod1 is initiated precociously, during gastrulation in the medial region of paraxial mesoderm (Weinberg et al., 1996; Coutelle et al., 2001). During the following segmentation period (there is not strict distinction between segmentation and neurulation in zebrafish), the lateral expression of Myf5 and Myod1 is also visible in the posterior region of each somite (Kimmel et al., 1995; Groves et al., 2005; Stellabotte et al., 2007). The primitive myotome in zebrafish is also made up of different muscle fiber populations (see *Muscle Fiber Population of the Primitive Myotome*) and is also the first somitic compartment to form before the dermomyotome (Stellabote et al., 2007).

### 2.2 The Second and Third Myogenic Waves Arise From Dermomyotome in *Xenopus*

The dermomyotome is defined as the part of a somite capable of generating both the dorsal dermis and the myotome (Christ and Ordahl, 1995). In amniotes, the four edges of the dermomyotome are the source of myoblasts, which will form the primary myotome, whereas the central region of the dermomyotome contains progenitor cells common to the dermis and the muscle (Gros et al., 2004; Ben-Yair and Kalcheim, 2005) (Figure 2A). These progenitors proliferate in the plane of the dermomyotome, and when they divide perpendicular to the plane of the dermomyotome, dermal and myogenic progenitors are generated dorsally and ventrally, respectively (Ben-Yair et al., 2011). Pax3 and Pax7, which play a role upstream of the Myod family of transcription factors, have been identified as dermomyotome marker in amniotes (Tajbakhsh et al., 1997; Relaix et al., 2005; Bajard et al., 2006). The Six family of transcription factors have also been identified as upstream factors during dermomyotome formation and skeletal myogenesis in mice (Grifone et al., 2005).

**FIGURE 3:**
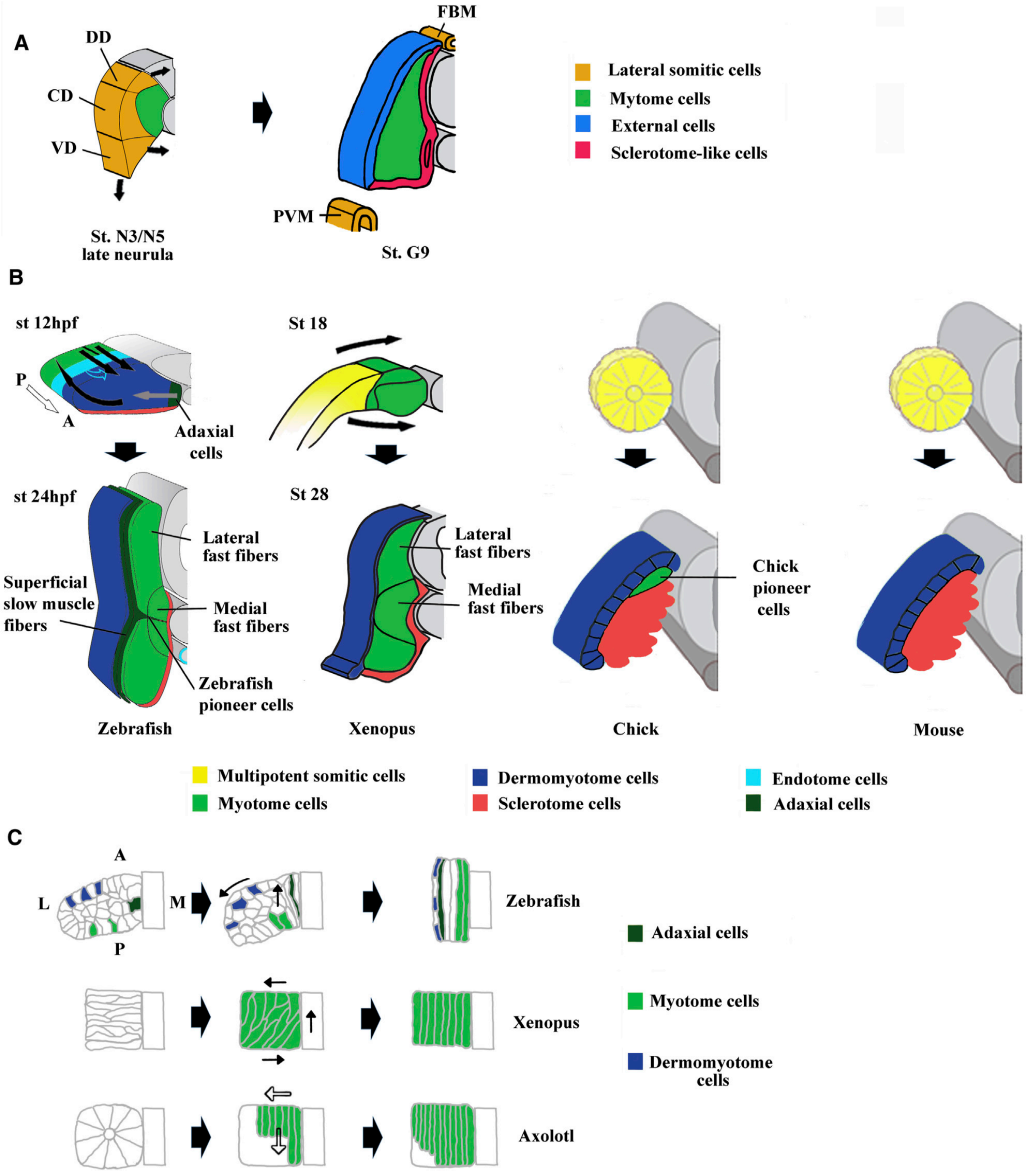
Comparison of compartmentalization modes between amphioxus, zebrafish, *Xenopus*, chick and mouse. **(A)** The lateral domain of amphioxus somite is already compartmentalized at mid neurula stage and possesses progenitors that give rise to the external cells layer, the sclerotome-like compartment but also the lateral plate mesoderm and the fin box mesothelium at stage G9. The lateral domain can be subdivided into three subdomains which express different set of genes. For example, Pax3/7 is expressed in the central domain (CD), Pax1/9 in the dorsal domain (DD) and Hand in the ventral domain (VD). Adapted from Yong et al. (2021). **(B)** The first phases of compartmentalization are both medio-lateral and antero-posterior in zebrafish, mainly medio-lateral in *Xenopus* and mainly dorso-ventral in chick and mouse. In zebrafish, an apparent movement of somite rotation relocated the different cell populations during the segmentation period. The posterior cells elongate toward the anterior region of the somites (straight black arrows) and give rise to fast fibers, the anterior cells were relocated in the surface outside of the somites (curved black arrows) and give rise to the dermomyotome. In addition, the medially adaxial cells migrate laterally toward the myotome periphery (grey arrows) and differentiate both into pioneer cells and superficial slow fibers. The endotome cells migrate toward the midline aorta (light blue hollow arrows). The appearance and location of MSCs are unknown and difficult to infer in zebrafish. Somites at 12 and 24 hpf (hour post fertilization). In *Xenopus*, lateral MSCs envelop the myotome ventrally and dorsally to give rise to the dermomyotome and the sclerotome (bended black arrows). Both in *Xenopus* and zebrafish, the lateral fast fibers are in dorsal and ventral position around the medial ones which are located close to the notochord. Somites at mid-neurulation (stage 18) and at tailbud stage (stage 28). In amniotes, chick, and mouse, the newly formed somites are naïve structures, made up of MSCs which subdivide into a dorso-lateral dermomyotome and a ventro-medial sclerotome. The dermomyotome cells remain epithelial whereas the sclerotome cells undergo EMT (epithelial mesenchymal transition). In chick, the pioneer cells begin to express Myf5 and Myod1 medially at epithelial somite stage, and become the first myocytes used as a scaffold for the construction of primary myotome. For zebrafish modified from Buckingham and Vincent, (2009) and Keenan and Currie, (2019). For chick and mouse, modified from Buckingham, (2001). **(C)** Comparison of muscle cell movements during somitogenesis between zebrafish, *Xenopus,* and axolotl. Zebrafish: Cell movements during apparent somite rotation. Lineage tracing of cells inside a somite makes it possible to follow their movements. Myogenic cells (curved arrow), Dermomyotome precursors (straight arrow). Explained in **(A)**. *Xenopus*: Myogenic cells are first oriented perpendicular to the antero-posterior axis, before becoming parallel to it during apparent somite rotation (black arrows). Axolotl: Differentiation of myogenic cells inside somites is characterized by cell elongation in antero-posterior direction progressing medio‐laterally (hollow arrows). Adaxial cells have been described in axolotl but are not represented here (Banfi et al., 2012). For zebrafish, modified from Stellabotte et al. (2007). For *Xenopus*, modified from Keller, (2000). For axolotl summarized from Neff et al. (1989), Radice et al. (1989), and Keller, (2000).

The existence of a dermomyotome in anamniote embryos has long been discussed, until Devoto et al. (2006) suggested that somites of all vertebrate embryos have a dermomyotome compartment. In cephalochordates, histological analyses show that the somitic dorso-lateral external cells are not equivalent to the vertebrate dermomyotome (Holland, 1996; Holland et al., 1999; Mansfield et al., 2015). However, pax3/7 has been detected in the dorso-lateral region of amphioxus somites indicating that these external cells possess some features of vertebrate dermomyotome (Yong et al., 2021). Moreover, the lateral somitic domain of amphioxus can be subdivided at mid/late neurula stage into three subdomains with specific expression of Pax3/7, Pax1/9, and Hand genes suggesting that somitic compartmentalization already exists in the ancestral chordates (Figure 3A). Pax3 and Pax7 orthologs are expressed in the dorso-lateral cells of somites in lampreys (Kusakabe and Kuratani, 2005), zebrafish (Groves et al., 2005), and sturgeons (Devoto et al., 2006), suggesting that the thin sheet of dorso-lateral cells of anamniotes is homologous with the amniote dermomyotome. Although dermal and myogenic bipotent progenitors have not been identified in anamniotes, the expression of collagen genes in the dorso-lateral cells of somites in teleosts and *Xenopus* argues that the dorsal dermal lineage originates from the dermomyotome (Grimaldi et al., 2004; Le Guellec et al., 2004; Rescan et al., 2005).

In *Xenopus*, a thin dorso-lateral tissue distinct from the underlying myotome has been first assimilated to a dermatome, at the origin of the dorsal dermis (Ryke, 1953; Hamilton, 1969). Moreover, Grimaldi et al. (2004) showed that this region is similar to the amniote dermomyotome since it expresses Pax3 and gives rise to myogenic cells at epaxial and hypaxial levels (Martin and Harland, 2001). The myogenesis at epaxial and hypaxial regions is in addition to that of the primitive myotome and has therefore been described as a second myogenic wave in *Xenopus* (Grimaldi et al., 2004; Della Gaspera et al., 2012a) (Figure 2B). Hence, it can be considered that the first wave of primitive myotome and the second wave of dermomyotome participate both to the formation of the primary myotome, which is initially made up of mononucleated fibers extending across a somite in an antero-posterior direction as is the case in amniote vertebrates (Gros et al., 2004) (Figure 2B). In *Xenopus*, there is a third myogenic wave at stage 36–37 characterized by the presence of Myf5-expressing cells within the myotome, which could participate in the growth of the myotome and the formation of multinucleated fibers from stage 45 onward (Grimaldi et al., 2004; Della Gaspera et al., 2012b) (Figure 2B). From stage 48, myogenin has also been identified in some isolated mononucleated cells located at the periphery of large larval myofibers (Nicolas et al., 1998; Chanoine and Hardy, 2003). This third wave undoubtedly corresponds with previously histologically identified mesenchymal cells that participate to the secondary myogenesis during the larval phase (Boudjelida and Muntz, 1987; Radice et al., 1989; Kiełbówna and Daczewska, 2005). Similarly, from stage 45–46 onward, satellite cells expressing Pax7 and positioned under the basal lamina are also observed within the myotome (Chen et al., 2006; Daughters et al., 2011). These satellite cells originate at the early neurula stage from the dorso-lateral region of the mesoderm, later identified as the LSF at the origin of both dermomyotome and sclerotome (Daughters et al., 2011; Della Gaspera et al., 2012b; Della Gaspera et al., 2019). Although this has not been directly demonstrated by lineage studies, these results suggest that satellite cells and the third-wave myoblasts are also derived from the dermomyotome in *Xenopus* like in amniotes and zebrafish (Gros et al., 2005; Relaix et al., 2005; Hollyway et al., 2007; Seger et al., 2011). On the other hand, it can be also noted that the dermomyotome (and/or sclerotome) contributes to the mesenchyme of dorsal fin in amphibians (Sobkow et al., 2006; Garriock and Krieg, 2007). In conclusion, the dermomyotome in anamniotes and amniotes possesses similar functions.

### 2.3 Muscle Fiber Population of the Primitive Myotome

#### 2.3.1 Comparison Between *Xenopus* and Zebrafish

In *Xenopus*, the myotome muscle fibers of the feeding tadpole must be fully functional from stage 45 onward. The surface of the myotome is constituted of a thin layer of slow-twitch muscle fibers, while the rest of the myotome is made up of fast-twitch fibers (Radice, 1995; Grimaldi et al., 2004). The spatially separated slow and fast-twitch muscle fiber organization is adapted to various types of ondulatory swimming of the larvae (Jayne and Lauder, 1994).

The development of these two types of fibers has been studied in zebrafish, where the slow fiber population forms first medially (Devoto et al., 1996; Stickney et al., 2000). The adaxial progenitors give rise to both slow pioneer cells that remain associated with the horizontal myoseptum and superficial slow fibers that migrate to the surface outside of the somites (Devoto et al., 1996; Jackson and Ingham, 2013) (Figures 3B, C). The slow-twitch muscle fiber differentiation is dependent on the hedgedog (Hh) signaling pathway (Coutelle et al., 2001). The fast fiber precursors appear in the posterior region of each somite, drawing a ray perpendicular to the rostro-caudal axis. They undergo an apparent 90°C rotational movement that positions the cells in the more medial part so that the fibers are oriented antero-posteriorly (Stellabotte et al., 2007; Hollway et al., 2007) (Figures 3B, C). There are two subpopulations of fast–twitch fibers, the medial one located around the notochord, whose formation is independent of Fgf8 and sensitive to Hh, and a more lateral one whose formation depends on Fgf8 (Devoto et al., 1996; Groves et al., 2005; Feng et al., 2006; Keenan and Currie, 2019).

In *Xenopus*, the same three muscle fiber subpopulations, slow-twitch, medial fast-twitch, and lateral fast-twitch, exist within the myotome. The population of slow-twitch fibers appears lately, at stage 31–32, with the development of the caudal part of the embryo (Radice, 1995; Grimaldi et al., 2004). The “adaxial” progenitors differentiate in an Hh-dependent way and migrate to the periphery as in zebrafish, but there are neither pioneer cells nor horizontal myoseptum (Janesick et al., 2017). In *Xenopus*, fast-twitch fibers are the first fibers to form and are composed of two cell subpopulations, a medial and a lateral one (Krneta-Stankic et al., 2010; Della Gaspera et al., 2012a). Specification of lateral fast fibers seems to be also more dependent on fgf8 than the medial ones (Della Gaspera et al., 2012b). The medial myotome is also sensitive to Hh (Martin et al., 2007). Such homologies between zebrafish and *Xenopus* suggest that the last common ancestor of the zebrafish and *Xenopus* (before the split between sarcopterygians and actinopterygians) had a similar organization with a medial population of fast fibers sensitive to Hh, a more lateral one dependent on Fgf and a population of slow fibers dependent on Hh, which migrated to the periphery (Grimaldi et al., 2004).

#### 2.3.2 Muscle Fibers and Somite Rotation in Anamniotes

During *Xenopus* somitogenesis, muscle fibers are first perpendicular to the rostro-caudal axis and then parallel to it (Hamilton, 1969; Youn and Malacinski, 1981a) (Figure 3C). This bending and elongation movement has also been initially described as a 90°C global rotational movement of the somite (Hamilton, 1969). The existence of the same rotational movement in zebrafish and some others but not all frogs pleads in favor of an ancestral feature (Kielbowna, 1981; Fan et al., 2001; Stellabotte et al., 2007; Hollway et al., 2007). However, the apparently synchronous rotation of somitic cells is not so homogenous and seems dependent on both cell location and differentiation state both in *Xenopus* and zebrafish (Youn and Malacinski, 1981a and b; Keller, 2000; Afonin et al., 2006; Yin et al., 2018). In both species, Cxcl12 (sdf-1α) is necessary to the apparent somite rotation, but in zebrafish, fast fiber elongation is also driven by slow fiber migration (Hollway et al., 2007; Leal et al., 2014; Yin et al., 2018). In *Xenopus*, depletion of miR-206, a key modulator of muscle development affects expression of adhesion proteins and somite rotation, suggesting that myogenic differentiation program could be coupled to somitic cell movements (Vergara et al., 2018). However, in the Urodela amphibian axolotl, somitic cells do not rotate, but are first organized around a central somitocel to constitute the rosette somite. Next, they elongate in antero-posterior direction progressing medio-laterally at the time of differentiation (Youn and Malacinski, 1981b; Neff et al., 1989) (Figure 3C). Although there are species-dependent variations in somitic movements, muscle differentiation program seems to be closely related to the orchestration of such movements.

#### 2.3.3 Evolutionary Origin of Distinct Muscle Fiber Populations

Little is known about the evolutionary emergence of different populations of muscle fibers in vertebrates. Nevertheless, slow-twitch fibers ontogenesis is known to be independently regulated during myotome formation. In zebrafish, the differentiation of slow-twitch fibers is independent of Myod1 and Myogenin contrary to the fast ones (Hinits et al., 2009 and 2011). The adaxial progenitors appear earlier than the fast ones, and slow-twitch fibers do not fuse (Stickney et al., 2000). Moreover, Prmd1, the key gene that initiates the slow-twitch program by repressing the fast one, is regulated by Hh, and it is still expressed in the double Myf5/Myod1 mutant devoid of muscle fibers (Hinits et al., 2009 and 2011). Hence, the question concerning the independent evolutionary origin of slow- and fast-twitch fiber populations naturally arises.

Although different muscle fiber populations have been described in the other groups of chordate, cephalochordates and tunicates, a phylogenetic link to slow and fast-twitch vertebrate fibers is not factually sustained (Flood, 1967). In adult amphioxus, superficial red fibers (slow) and deep white fibers (fast) have been described in the myotome but their ontogenesis is unknown (Holland, 1996; Lacalli and Kelly, 1999). Tunicates are the sister group of vertebrates within chordates. The adult tunicate is a sessile species even though larvae are motile. Tunicates have lost somites, but the larvae still possess myocytes in the tail. Most of the tail myocytes are specified very early during development by Macho-1 (Razy-Krajka and Stolfi, 2019). Tunicate genome has evolved very rapidly and it has lost key developmental genes contributing to their specific morphologies and motility in the chordate phylum (Onai et al., 2018). There are indeed five different genes encoding sarcomeric myosin heavy chains in tunicates, but they appeared after the vertebrate–tunicate split (Razy-Krajka and Stolfi, 2019). In the same way, comparison of ortholog genes for sarcomeric proteins between distinct groups of deuterostomes indicates that independent duplication events inside each group are at the origin of myofiber isoforms (Inoue and Satoh, 2018). These data suggest that fiber type similarities between different groups of chordates are homoplasies.

In the cyclostome vertebrate, the lamprey, no slow fibers were found in the surface outside of the myotome, but parietal slow fibers envelop central fast muscles inside multiple muscle units that constitute the adult myotome (Teräväinen, 1971; Vogel and Gemballa, 2000). However, the lamprey larval myotome is composed only of fast fibers and no slow-twitch progenitors have been observed despite the presence of an adaxial proto-program (Kusakabe and Kuratani, 2005; Hammond et al., 2009). In adult lamprey, slow-twitch fibers could arise from progenitors derived from dermomyotome since in *Xenopus* and zebrafish, a second wave of slow fibers can also be produced by adaxial-independent progenitors coming from the dermomyotome (Barresi et al., 2001; Grimaldi et al., 2004). Adaxial progenitors have been described in sturgeon, zebrafish, and *Xenopus* (Devoto et al., 1996; Grimaldi et al., 2004; Steinbacher et al., 2006). In addition, superficial slow-twitch fibers are present on the myotome surface of both cartilaginous and bony fishes (Bone, 1966; Bone, 1978). In this context, the evolutionary origin of adaxial cells probably dates back to the ancestor of all or gnathostome vertebrates.

### 2.4 Myogenic Programs

**FIGURE 4:**
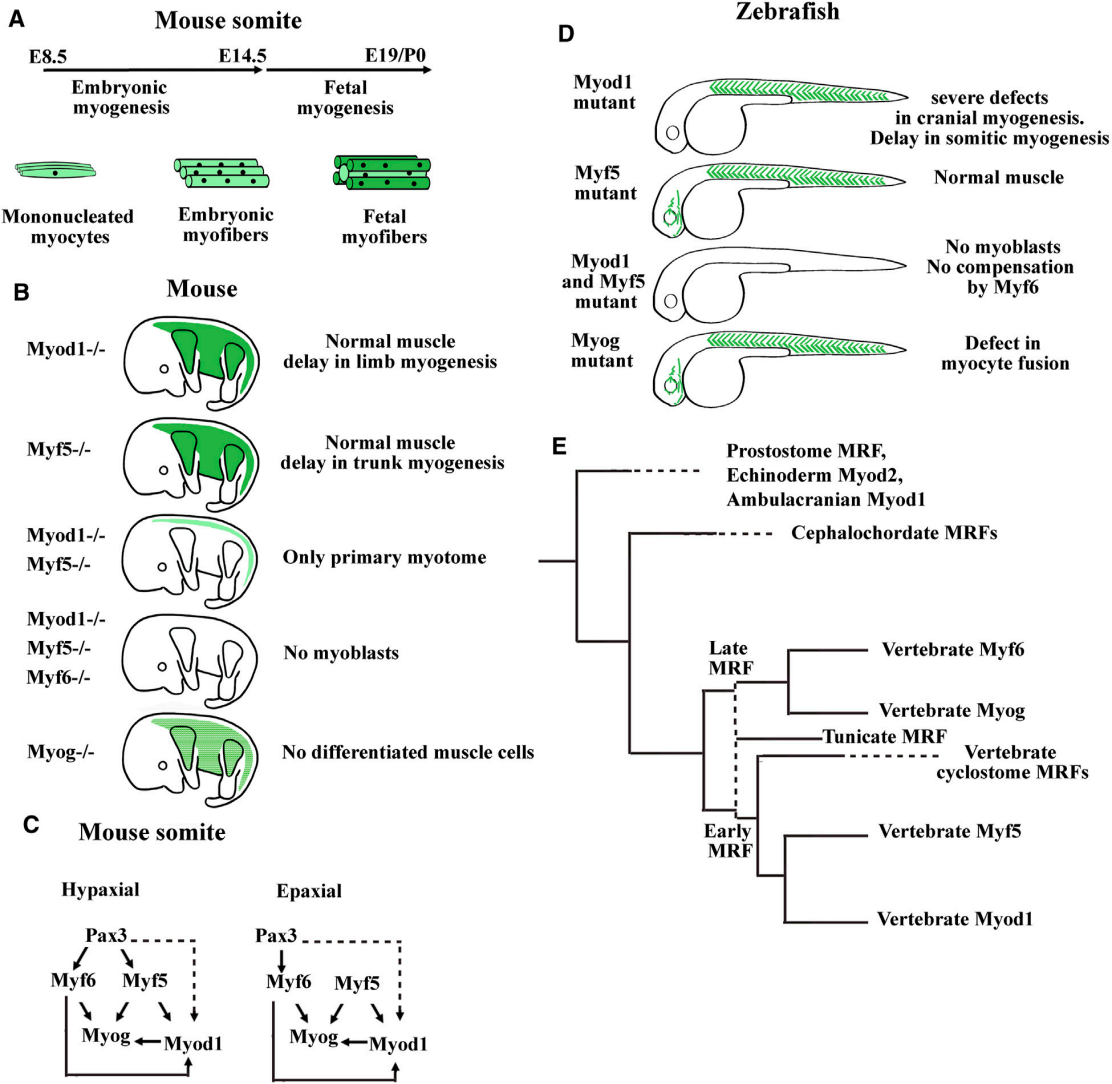
Summary of myogenic regulatory factor (MRF) functions in mouse and zebrafish models. **(A)** Timing of myofibers formation during embryonic and fetal myogenesis in mice. **(B)** Summary of the main phenotypes of single KO mice for Myod1, Myf5, or Myogenin, double KO mice for Myod and Myf5, and triple KO mice for Myod1, Myf5, and Myf6. Myoblasts formation requires either Myod1 or Myf5. Myog is necessary for muscle differentiation and Myf6 can compensate the absence of Myod1 and Myf5 only during embryonic myogenesis. Myf6 KO mice did not show muscle development defects. Modified from Hernández-Hernández et al. (2017). **(C)** Genetic hierarchy during embryonic myogenesis in epaxial and hypaxial domain of the somite. Mice invalidated for the three genes Pax3, Myf5, and Myf6 show an absence of all skeletal muscles in the trunk indicating that these factors act upstream of Myod1. While the myogenesis in the hypaxial domain is Pax3 dependent, a program initiated by Myf5, Pax3 independent exists in the epaxial domain. Dashed lines indicate that the regulation of Myod1 expression by Pax3 is probably indirect through Pitx2 and Six transcription factors. Head mouse myogenesis is not presented here. For a more detailed analysis of MRF KO mice, see Bismuth and Relaix (2010), Comai et al. (2014), and Buckingham (2017). **(D)** Main phenotypes of zebrafish single mutants for Myod1, Myf5, or Myog and double mutant for Myod1 and Myf5. The zebrafish mutant for Myf6 did not show abnormal muscle development. In zebrafish, Myod1 is necessary for normal cranial muscle development whereas Myf6 does not compensate the absence of Myod1 and Myf5. **(E)** Summary of the MRF phylogeny in bilaterians. Aase-Remedios et al. (2020) propose that the four vertebrate genes coding for MRFs do not result from two rounds of whole genome duplication (2R WGD) of a single ancestral gene, that would have taken place between ancestral chordates and vertebrates. Instead, a cluster of two MRF genes generated by tandem duplication predates the 2R WGD. One gene of this cluster generates *via* 2R WGD and gene losses the early vertebrate MRFs (Myf5 and Myod1), and the other generates the late vertebrate MRFs (Myf6 and Myogenin). The first vertical dashed line indicates that tunicate MRFs could be the orthologs of either the early or the late ancestral MRF gene preceding the 2R WGD. The two MRF genes present in cyclostome species could be the orthologs of the early MRF gene. Cyclostomes would have diverged from gnathostomes after the first R WGD and before the second R WGD (Nakatani et al., 2021). Horizontal dashed lines indicate that the next duplication events in the branch are not shown here. Modified from Aase-Remedios et al. (2020).

The function of the four myogenic regulatory factors (MRFs) of the Myod family (Myod1, Myf5, Myf6, and Myogenin) in the specification, determination, and differentiation of muscle fibers has been studied in numerous single, double, and triple knockout experiments in mice (Bismuth and Relaix, 2010; Yamamoto et al., 2018). Single Myf5 and Myod1 knockout mice survive whereas the double knockout mice die due to the absence of myoblasts (Rudnicki et al., 1993; Kassar-Duchossoy et al., 2004) (Figures 4A–C). Thus, Myf5 and Myod1 were defined as determination factors that could partly compensate each other. For instance, the existence of two myogenic lineages that could compensate each other has previously been proposed, one that is Myf5-dependent and the other being Myf5-independent, and probably driven by Myod1 (Haldar et al., 2008). Experiments based on the conditional expression of cell-killer gene, the diphtheria toxin, activated by the Cre recombinase under the control of the Myf5 promoter indicate that Myod1 is unable to compensate the lost Myf5 lineage suggesting that compensation between Myf5 and Myod1 is due to a functional redundancy of the two proteins (Comai et al., 2014). Surprisingly, the morphant and mutant studies in zebrafish have come to slightly different conclusions (Figure 4D). In zebrafish, double mutants for Myf5 and Myod1 have any myoblasts but Myod1 mutants also die since Myod1 drives lateral fast fibers myogenesis in somites and is essential for cranial myogenesis (Hinits et al., 2009; Hinits et al., 2011). Another study with morpholino oligos have found that both Myf5 and Myod1 are each necessary to the development of some muscle anlagen in the head (Lin et al., 2006). In *Xenopus*, analysis of the role of the three MRFs (Myod1, Myf5, and Myf6) during primitive myotome formation using gene knockdown experiments revealed that Myod1 is necessary for lateral myogenesis (Della gaspera et al., 2012b) as previously observed in zebrafish (Hinits et al., 2009). Hence, in anamniotes, Myf5 cannot fully compensate the absence of Myod1. During *Xenopus* head myogenesis, each of these two determination factors is weakly expressed or not expressed with the other one in some anlagen (Della Gaspera et al., 2012a). It is also the case for Myf5 in zebrafish that is absent from superior, medial, and lateral rectus muscles anlagen (Lin et al., 2006). Indeed, Myf5 and Myod1 have not the same abilities to initiate the differentiation. Myod1 is the better inducer, suggesting that variation of expression of each MRF inside the same anlage could have an effect on differentiation timing (Ishibashi et al., 2005; Della Gaspera et al., 2012a; Conerly et al., 2016).

Regarding the *in vivo* function of the differentiation factor Myogenin, the knockout mice die due to the absence of differentiated muscle fibers whereas in zebrafish, the Myogenin mutants survive with defects in myocyte fusion (Hasty et al., 1993; Ganassi et al., 2018). In mice, it was also shown that Myogenin was not necessary for the formation of the primary myotome (Venuti et al., 1995) (Figures 2A and 4A). In *Xenopus*, Myogenin is weakly expressed during the first myogenic wave and strongly expressed at the hypaxial and epaxial edges during the second myogenic wave at stage 32–34 suggesting that Myogenin may be necessary for the formation of the multinucleated fibers from stage 45 in *Xenopus* (Kielbowna and Dacszewska, 2005; Della Gaspera et al., 2012a) (Figure 2B).

Distinct functions have also been highlighted for Myf6 between mice and anamniotes. In mice, Myf6 acts as both a determination and a differentiation factor. Myf6 is able to initiate myogenesis in the absence of Myf5 and Myod1 during myotome formation but not during fetal myogenesis (Figures 4A–C). Furthermore, Myf6 acts upstream of Myod1 during extraocular myogenesis (Kassar-Duchossoy et al., 2004; Sambasivan et al., 2009). In contrast, in zebrafish, Myf6 is unable to initiate myogenesis in the double Myf5/Myod1 mutants, and in *Xenopus*, Myf6 is always the last MRF expressed in the head and in the myotome (Schnapp et al., 2009; Hinits et al., 2011; Della Gaspera et al., 2012a) (Figure 4D). Until now, the results obtained in anamniote species suggest that the role of Myf6 as a determination factor is not an ancestral function.

In any case, it seems that the transition from anamniotes to amniotes impinges on the MRF functions not just in somites but also in the head. The reorganization of MRF core networks could be an indication of the evolution of the compartmentalization process in somites as we describe it, and could mean that head myogenesis has also dramatically evolved. Recently, the phylogenic tree of Myod family has been redefined (Aase-Remedios et al., 2020) (Figure 4E). The ancestral vertebrate could possess only two clustered MRF genes, one acting early like a determination factor and the other acting late like a differentiation factor. The cyclostome vertebrate species, lamprey and hagfish, each have only two MRF genes, both derived from the same early MRF gene. The increase in gene number of the Myod family with four members derived from the early and late ancestral MRFs in gnathostome vertebrates has probably contributed to the higher complexity of muscle formation and composition.

### 2.5 Sclerotome

In vertebrates, the sclerotome is mainly at the origin of the vertebrae and ribs but vascular and tendon cells also derive from specialized sclerotome parts (Scaal, 2016; Tani et al., 2020). In amniotes, the newly formed somite subdivides into two easily discernable compartments, the sclerotome that represents about half of the somite size and the dermomyotome. The dermomyotome consists of cells that remain in epithelial state, whereas the sclerotome cells undergo EMT (epithelial–mesenchymal transition) and migrate around the notochord and neural tube. While the sclerotome is a relatively large compartment in amniotes, it is reduced to a thin sheet of cells on the ventro-medial side of the huge myotome in anamniotes (Figure 1A).

In *Xenopus*, initial morphological and histological works reported the identification of some polymorphic cells at the late tailbud stage in the ventro-medial edge of the somites that could constitute the sclerotome compartment (Ryke, 1953; Youn and Malacinski, 1981b). At the later stages, in both urodele and anuran species, sclerotomal cells migrate into the perinotochordal and perineural space and give rise to the axial skeleton (Mookerjee, 1930; Mookerjee, 1931). The late formation and migration of the sclerotome cells in amphibian is supposed to be due to delayed vertebral development at the end of the larval stage (Scaal and Wiegreffe, 2006). The ventro-medial location of sclerotome cells was confirmed more recently by *in situ* hybridization analysis with the main specific markers of the sclerotome which are not expressed in the dermomyotome and the myotome such as, Pax1 and Pax9 but also with other markers such as Twist1, Uncx, Foxc1, and Foxc2 mRNAs (El-Hodiri et al., 2001; Sánchez and Sánchez, 2013; Della Gaspera et al., 2019). Two or three domains in the sclerotome (dorsal, notochord-associated, and ventral) have been characterized in zebrafish and *Xenopus*, on the basis of differential expression of markers (Sánchez and Sánchez, 2013; Ma et al., 2018; Sánchez and Sánchez, 2021). The dorsal domain could also contribute to dorsal fin at least in fish (Freitas et al., 2006; Ma et al., 2018). Until now, little is known about the gene functions involved in the sclerotome formation in anamniotes. Knockdown experiments of Pax1, Pax9, and Twist1 in medaka have confirmed the essential function of these genes in sclerotome formation with a pronounced subfunctionalization for Pax1 and Pax9 (Yasutake et al., 2004; Mise et al., 2008). Any functional study of the sclerotome genes has yet to be done in *Xenopus*. Due to the late formation of sclerotome, long after the dermomyotome and myotome, its developmental origin in anamniotes has been questioned; it could derive from myotome cells or from a separate population still unidentified (Keller, 2000). Indeed, we recently identified Twist1 as a marker of migrating sclerotome progenitors in two amphibians, *Xenopus* and axolotl, and showed that both sclerotome and dermomyotome cells originate from a cell population located at LSF revealing the ancestral location of MSCs (Della Gaspera et al., 2012b; Della Gaspera et al., 2019).

### 2.6 Syndetome

In amniotes, the syndetome is the somitic compartment at the origin of the dorsal tendons (Brent et al., 2003) (Figure 1A). Syndetome is a dorsal compartment of the sclerotome, between two neighboring myotomes, induced by fgf8 secreted by muscle cells (Brent et al., 2003; Brent and Tabin, 2004). It could be noted that the acquisition of tendon fate is highly dependent of local cellular environment. Double knockout mice for Sox5 and Sox6, two transcription factors involved in chondrocyte differentiation saw an expansion of the syndetome at the expense of cartilage differentiation only in the somites, whereas limb tendons form normally (Brent et al., 2005). Similarly, muscle tissue and fgf signaling are necessary for tendon progenitor specification in the somites only, whereas muscle tissue remains still essential for tendon differentiation in the limb and head (Kardon, 1998; Bonnin et al., 2005; Brent et al., 2005; Grenier et al., 2009).

The earliest and specific marker of tendons and ligaments is Scleraxis, a bHLH transcription factor of the Twist family (Schweitzer et al., 2001) that regulates genes involved in tendon differentiation such as Tenomodulin and Col1a1 encoding two extracellular matrix proteins (Shukunami et al., 2006 and, 2018; Léjard et al., 2007). Scleraxis is also necessary for the recruitment of some tendon progenitors at the elongation sites of the longest tendons (Huang et al., 2019). However, Scleraxis is not the only master gene of tendinogenesis since the tendon progenitors appeared normal in Scleraxis knockout mice (Murchison et al., 2007). Nevertheless, in these mice, some of the tendons present a defect in differentiation with the extracellular matrix less organized, tenomodulin expression lost, and collagen I expression reduced. A somitic scleraxis-positive compartment, giving rise to tendons, was also identified in *Xenopus* (Della Gaspera et al., 2009) and in the following years in fish species (Bricard et al., 2014; Ma et al., 2018). In zebrafish and probably in *Xenopus*, tenocytes adopt a particular cellular morphology with cell bodies positioned at the sclerotome edge and cytoplasmic extensions of tree-like processes slip into intersomitic space at myotendinous junctions. In zebrafish, as well as in trout and *Xenopus* likely, tenocytes originate from sclerotome indicating that the generation of tenocytes from the sclerotome dates back at least to the last common ancestor of the sarcopterygians and the actinopterygians (Bricard et al., 2014; Ma et al., 2018). Tendon development studies in zebrafish have confirmed that muscle tissue is only necessary for the formation of dorsal tendons, but it is still important for the differentiation of the fin and the cranial tendons (Chen and Galloway, 2014). More recently, crispr/cas9 has permitted the generation of Scleraxis mutants where both genes coding for Scleraxis (a and b) are mutated, leading to tendon differentiation defects particularly in the head and to deficiencies in rib mineralization and muscle growth. These results illustrate the co-development of the musculoskeletal system (Kague et al., 2019).

**FIGURE 5:**
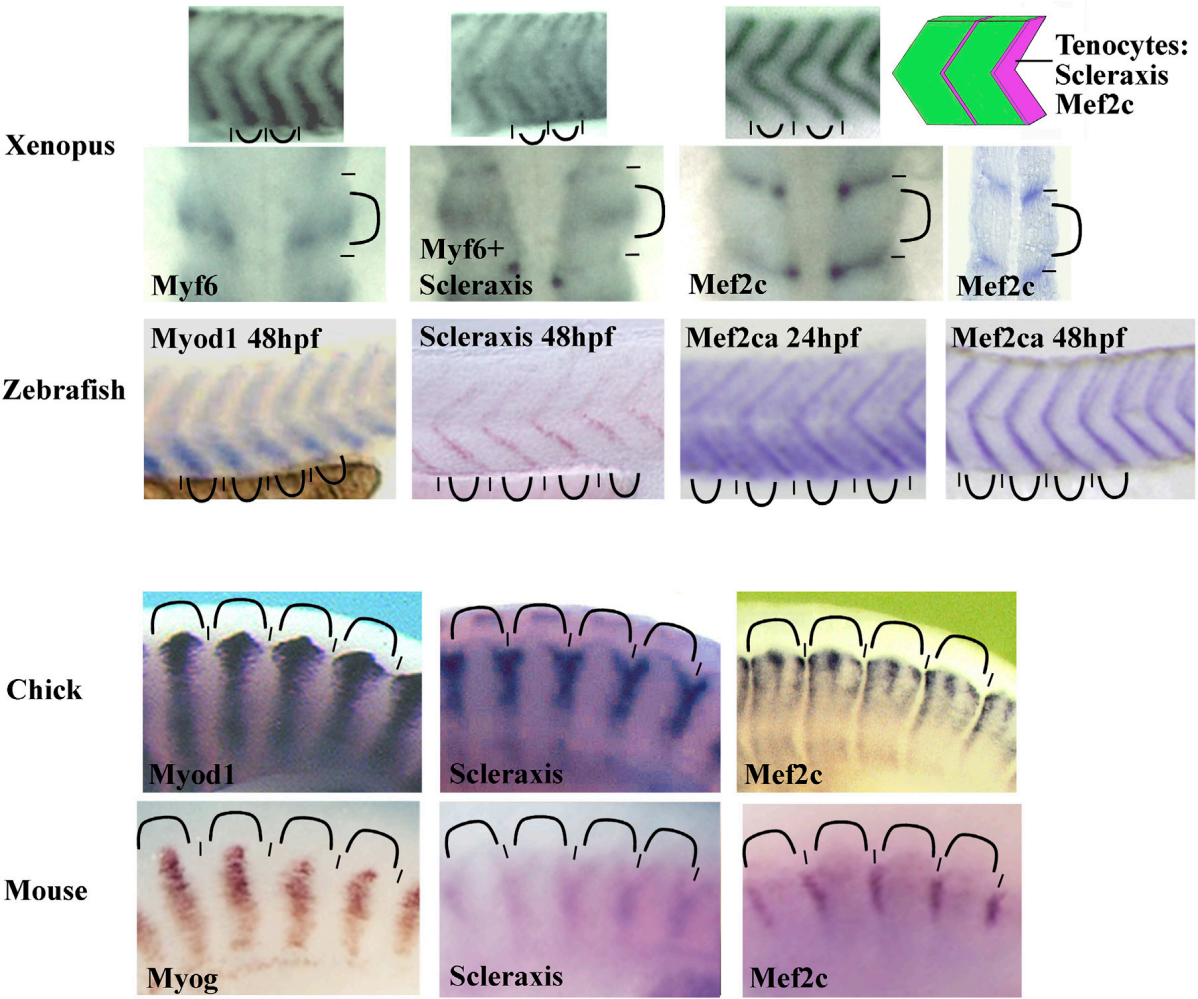
Comparison of the somitic Mef2c mRNA expression between zebrafish, *Xenopus*, chick, and mouse. The Mef2c expression is conserved in vertebrate somites. The staining is intense in intersomitic region corresponding to the syndetome where the tenocytes differentiate (schematic on the top right corner). In chick, the staining is reduced to muscle-associated tissue. The Mef2c expression is compared with both Scleraxis (syndetome marker) and Myod1, Myf6, or Myogenin (myotome markers). The somitic blocks are indicated by curved lines and the intersomitic regions by short lines. For zebrafish, the Mef2ca expression at 24 and 48 hpf (hours post fertilization). The probe is indicated in each image. All images are lateral views except the second row for *Xenopus* where the first three images at the left are dorsal views and the forth image at the right is a front view. Figure is composed of images from ISH database of ZFIN for zebrafish (zfin.org), Geisha for chick (geisha.arizona.edu) and Embrys for mouse (embrys.jp).

To date, Scleraxis is the earliest and most specific tendon marker. However, we have observed Mef2c expression in *Xenopus* syndetome before Scleraxis (Della Gaspera et al., 2009). Mef2c belongs to the MEF2 family of transcription factors with Mef2A, B, and D in gnathostome vertebrates. They are involved in the development of numerous mesoderm derivatives like smooth, cardiac, and skeletal muscles, and also in neuron differentiation (Potthoff and Olson, 2007). In *Xenopus*, Mef2c mRNA is detected in muscle-associated connective tissue not only in somites but also in the hypaxial and cranial muscles. In addition, Mef2c mRNA colocalizes with scleraxis mRNA at later stages (Della Gaspera et al., 2009). These results suggest that Mef2c could be involved precociously in larval tendon development. When *in situ* hybridization results are compared between various species, the Mef2c expression profile appears to be conserved in anamniote and amniote species (Figure 5). Mef2c has also been identified by RNA-seq screening in mouse tendon progenitors (Havis et al., 2014). Moreover, it appears in zebrafish that Mef2c expression is visible in intersomitic space at 24 hpf and Scleraxis at 36 hpf suggesting that Mef2c precedes Scleraxis expression in this region (Ganassi et al., 2014; Ma et al., 2018). Interestingly, Mef2c expression in chick and in mice remains closely associated with muscle tissue in a subdomain of Scleraxis-expressing cells suggesting a specific role in this part of connective tissue associated with muscle (Figure 5). Gain-of-function experiments in *Xenopus* have also shown a synergistic effect between the two transcription factors, Mef2c and Scleraxis, on the expression of two genes known to be expressed in tendon cells, Tgfβi, and Tenascin c (Della Gaspera et al., 2009). Nevertheless, to date, any experiment of loss of function has demonstrated the implication of Mef2c in muscle-associated connective tissue, tendon, or myotendinous junction development. Indeed, Mef2c knockout mice die at E10.5 as a result of a cardiac and vascular malformation making it difficult to study tendon formation. Nevertheless, it has been shown that Mef2c is involved in the formation of the heart and endothelial cells and has a role in cranial neural crest development and chondrocyte hypertrophy (Lin et al., 1997, 1998; De Val et al., 2004; Arnold et al., 2007; Verzi et al., 2007). However, when one of the two Mef2c genes in zebrafish, Mef2ca, is mutated, a minor defect in the formation of some head ligaments is observed (Nichols et al., 2016). Ideally, to study the function of Mef2c in muscle-associated connective tissue, loss-of-function experiments should be performed specifically in these cells. The cre/lox technique could be used to direct the Cre recombinase activity in the sclerotome with Pax1 promoter, but this could also affect chondrocyte development. In order to direct Cre activity in tendon progenitors, Scleraxis promoter could be used but Mef2c could act earlier. An alternative strategy could be to mutate a potential enhancer that could control specifically the expression of Mef2c in these particular cells of connective tissue.

### 2.7 Endotome, endothelial cells, and smooth muscle cells

**FIGURE 6:**
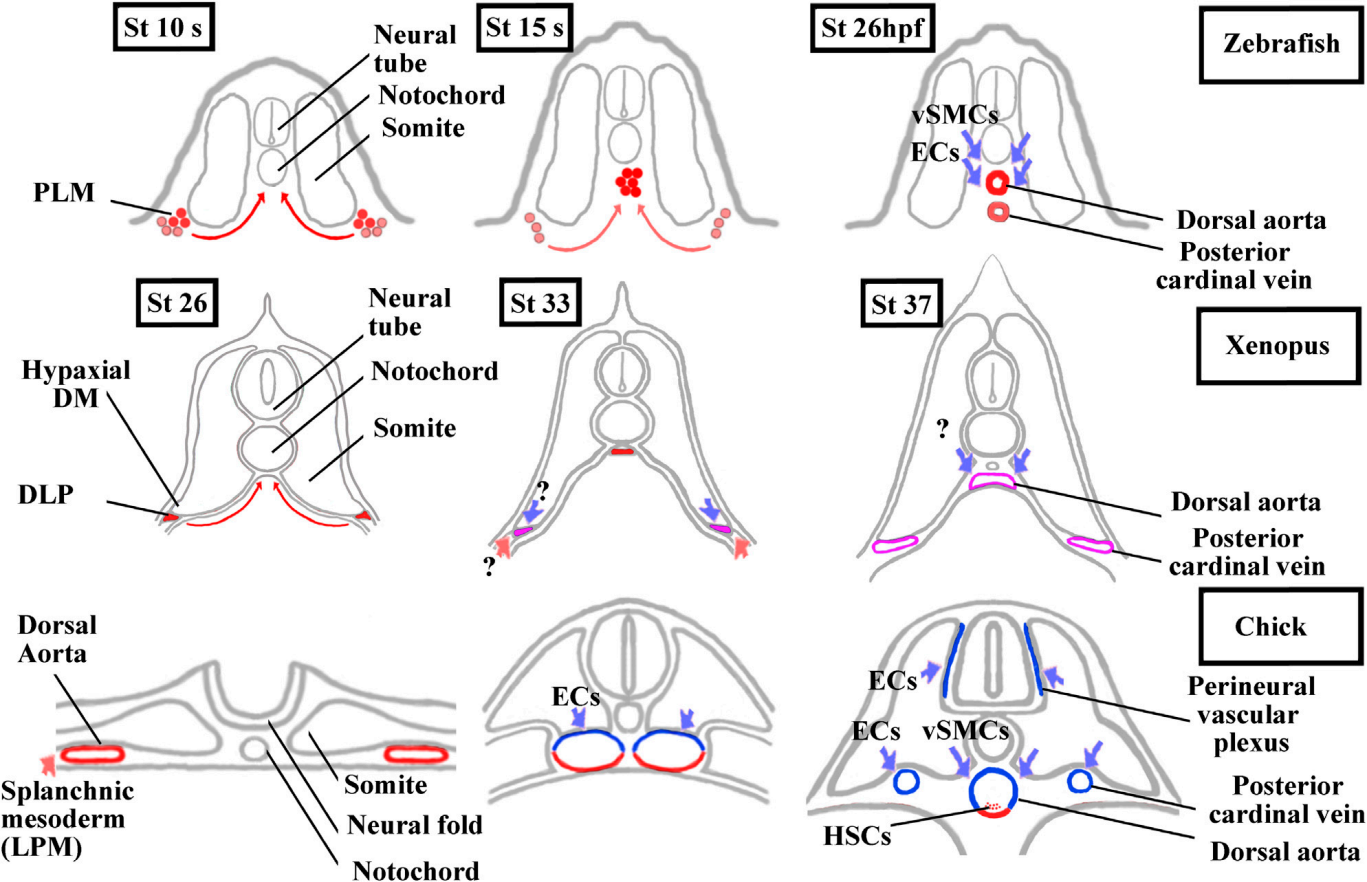
Comparison of aorta and posterior cardinal vein formation and their somitic contributions between zebrafish, *Xenopus,* and chick. The ECs and vSMCs that make up the blood vessels throughout the body have various origins. While ECs are exclusively from the splanchnic or the somitic mesoderm, vSMCs are derived from the neural crest and, from the splanchnic or the somitic mesoderm (Pardanaud et al., 1996; Pouget et al., 2006 and, 2008; Etchevers et al., 2001). The aorta formation has also been the focus of intense research in vertebrates as the adult hematopoietic stem cells are generated from the ventral aortic hemangioblasts. This bipotent progenitors can also differentiate into endothelial cells (Pardanaud et al., 1996; Pardanaud and Dieterlen-Lièvre, 1999; Ciau-Uitz et al., 2010; Ciau-Uitz and Patient, 2016). In zebrafish, the aorta hemangioblasts are the first to migrate from the PLM to the midline, coalesce, and form the single aorta. A distinct population of endothelial cells migrates later from the PLM to the midline to form the posterior cardinal vein. ECs and vSMCs from the somites contribute to the aorta and probably to the posterior cardinal vein maturation. Modified from Kohli et al. (2013) and Hogan and Schulte-Merker (2017). In *Xenopus*, a single aorta is also made up of migrating hemangioblasts from DLP, whereas a pair of bilateral cardinal veins appears at trunk level. Until now, the somitic contributions to the aorta and bilateral cardinal veins are unknown. Modified from Cleaver and Krieg, (1998), Ciau-Uitz et al. (2000), and Charpentier et al. (2015). In chick, a pair of bilateral aorta is first formed from the lateral plate mesoderm before fusing at the midline and receiving ECs and vSMCs from the somites. The bilateral posterior cardinal veins are formed of ECs from the somites. The endotome remains difficult to characterize in amniotes since it seems that ECs derive from several somitic regions (Wilting et al., 1995; Nimmagadda et al., 2005). Modified from Sato, (2013) and Jaffredo et al. (2013). In red, lateral plate mesoderm derived cells. In blue, somite-derived cells. In purple, unknown origin. DLP, dorsal lateral plate mesoderm; DM, dermomyotome; ECs endothelial cells; HSCs, hematopoietic stem cells; LMP, lateral plate mesoderm; PLM, posterior lateral plate mesoderm; vSMCs, vascular smooth muscle cells.

Somites also give rise to populations of endothelial cells (ECs) and vascular smooth muscle cells (vSMCs) that make up blood vessels (Pouget et al., 2006; Wilting and Becker, 2006; Ben-Yair and Kalcheim, 2008; Hirst and Marcelle, 2015). We should also add that somites give rise to another cell type, the adipocyte at least in mice (Sanchez-Gurmaches and Guertin, 2014; Sebo et al., 2018). In chick, somitic ECs give rise to trunk, abdominal wall, limb vessels, and also to lymphatic ones (Pardanaud et al., 1996; Ambler et al., 2001; Wilting and Becker, 2006; Sato, 2013). The contribution of somitic vSMCs is limited to the aorta in the trunk region and to the vessels in the limb and abdominal wall (Pouget et al., 2008; Wang et al., 2015). These results obtained in chick have only been partly extended to mice indicating particularly that some limb ECs emanate from somites (Mayeuf-Louchart et al., 2014). In mice and chick, the trunk aorta is first bilaterally paired before fusing at the midline. It is formed initially from the hemangioblastic splanchnic mesoderm, then two waves of somitic ECs from lateral epithelial somite/hypaxial region of the dermomyotome renew the aorta walls (Pouget et al., 2006; Jaffredo et al., 2013) (Figure 6). Next, somitic vSMCs cover the ECs layer of the aorta but it is not clear whether vSMCs originate from sclerotome or dermomyotome (Esner et al., 2006; Wiegreffe et al., 2007; Pouget et al., 2008; Ben-Yair and Kalcheim, 2008; Mayeuf-Louchart et al., 2014). Interestingly, Ben-Yair and Kalcheim, (2008) suggest that the lateral somite region is the source of multipotent progenitors which give rise to skeletal muscle, smooth muscle and endothelial cells.

In anamniotes, both in zebrafish and *Xenopus*, a single aorta is formed at the midline below the notochord (Figure 6). The hemangioblasts of the bilateral dorsal lateral plate mesoderm (posterior lateral plate mesoderm (PLM) in zebrafish and dorsal lateral plate mesoderm (DLP) in *Xenopus*) migrate to the ventral side of somites in medialward direction and coalesce into the aorta (Cleaver and Krieg, 1998; Zhong et al., 2001; Ciau-Uitz et al., 2010; Kohli et al., 2013; Ciau-Uitz and Patient, 2016). In addition, a new somite compartment, marked by cxcl12 and called the endotome, is at the origin of somitic ECs in zebrafish (Nguyen et al., 2014; Keenan and Currie, 2019). (Figures 3B and 6). In zebrafish and in chick, the origin of vSMCs and pericytes of the aortic wall has been identified in the sclerotome (Stratman et al., 2017; Rajan et al., 2020) (Figure 6).

In *Xenopus*, neither EC nor vSMCs originating from somites have yet been identified. Interestingly, the formation of the posterior cardinal vein (PCV) in *Xenopus* is closer to the amniotes than to the zebrafish (Figure 6). In zebrafish, the single cardinal vein results from migration and aggregation at the midline of ECs from the PLM, but it has been suggested that somitic ECs could also contribute to this (Isogai et al., 2001; Kohli et al., 2013; Nguyen et al., 2014; Hogan and Schulte-Merker, 2017). In *Xenopus* and amniotes, a pair of nascent cardinal vein appears bilaterally before fusing medially (Cleaver et al., 1997; Levine et al., 2003). In chicks, they are formed from somitic ECs, and in *Xenopus*, the question on whether somitic ECs could participate in the formation of the PCV and aorta is raised (Pardanaud et al., 1996; Pouget et al., 2006).

## 3 The signaling Pathways in Somitic Compartmentalization and Cell Fate

### 3.1 FGF Signaling

**FIGURE 7:**
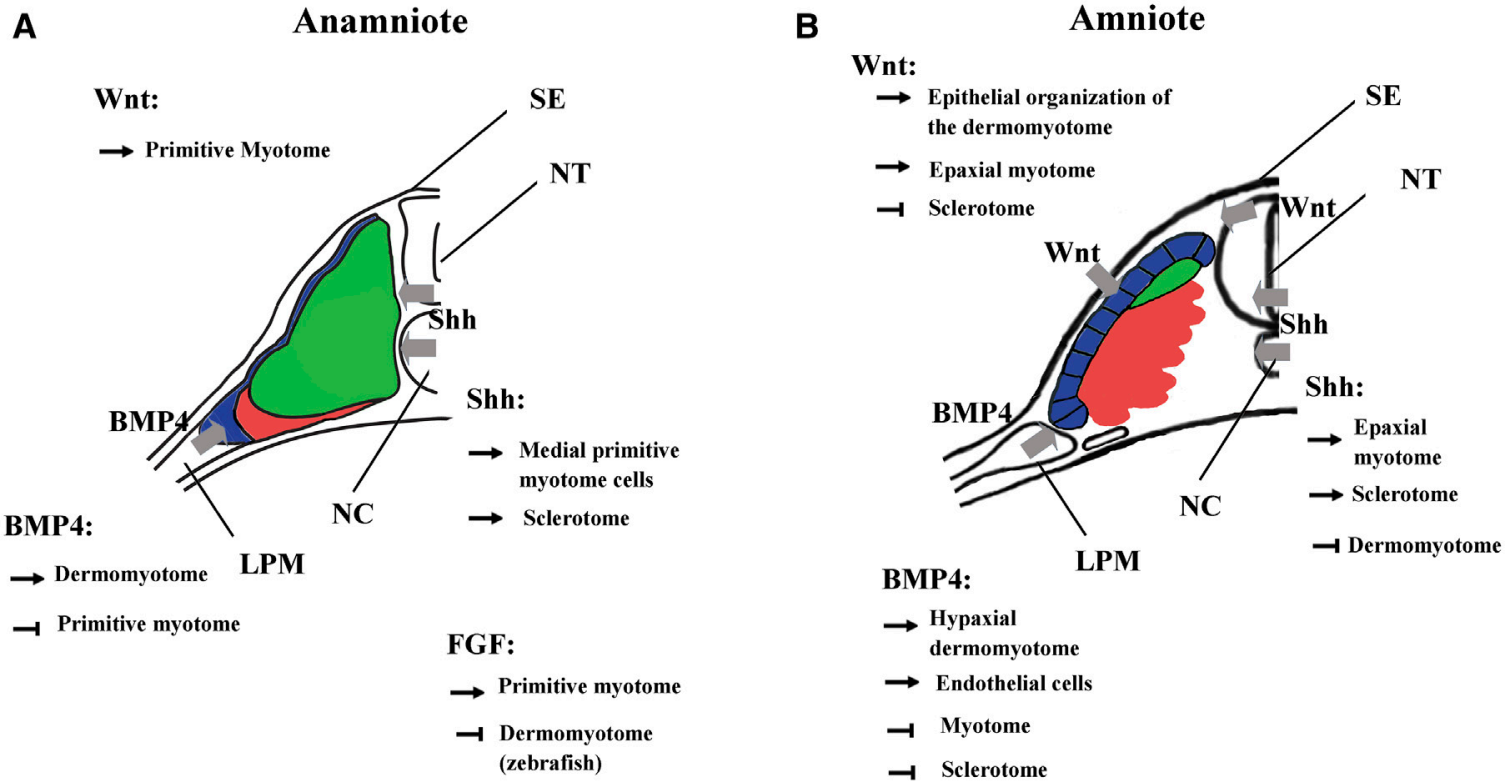
Main functions of BMP, FGF, Wnt, and Shh signalings during somite compartmentalization in anamniotes **(A)** and amniotes **(B)**. Somite compartmentalization depends on signals expressed by surrounding tissues. Shh is expressed by notochord (NC) and floor plate, Wnt by surface ectoderm (SE) and dorsal neural tube (NT) and BMP4 by lateral plate mesoderm (LPM). Most of the functions fulfilled by the signaling molecules seem to be conserved between anamniotes **(A)** and amniotes **(B)**. However, it seems that Fgf and to a lesser extent Wnt may play an early role in the formation of primitive myotome only in anamniotes **(A)**. Arrows, promoting effect; T-shaped line, inhibitory effect.

Fgf signaling acts at multiple levels in somite formation in anamniotes. In both zebrafish and *Xenopus*, Fgf favors the formation of dorsal structure like the paraxial mesoderm, the notochord, and the neural tube (Fürthauer et al., 2004; Fletcher and Harland, 2008). More precisely, in *Xenopus*, the use of an Fgf receptor antagonist (SU5402) has shown that Fgf signaling is necessary for the specification of the presumptive paraxial mesoderm and the maintenance of gene expression in the Spemann’s organizer, but not for the mesoderm induction (Fletcher and Harland, 2008). In animal cap assays, Fgf is able to induce Tbxt and Myod1 expression, two genes important for paraxial mesoderm specification and primitive myotome construction (Fisher et al., 2002; Fletcher et al., 2006; Fletcher and Harland, 2008) (Figure 7). Inversely, Fgf signaling inhibits ventral mesoderm specification (Kumano and Smith, 2000; Walmsley et al., 2008). Later, Fgf signaling counteracts BMP and favors the paraxial mesoderm fate at the expense of lateral mesoderm as shown in zebrafish (Row et al., 2018). Inside the paraxial mesoderm, Fgf8 also drives the lateral myogenesis and Myod1 expression in both *Xenopus* and zebrafish (Groves et al., 2005; Della Gaspera et al., 2012b). In addition, this is made at the expense of dermomyotome formation in zebrafish (Groves et al., 2005). The role of Fgf in somite formation can be traced back to the chordate ancestor as Fgf signaling plays a role in anterior somite formation in Amphioxus but seems to exert a more specific role in the vertebrate “clock and wavefront” system (Bertrand et al., 2011 and 2015).

Fgf signaling also acts at multiple levels on somite formation in mice. For instance, it acts early at the primitive streak and is necessary for morphogenetic movement and specification of paraxial mesoderm since without Fgf receptor 1, expression of Tbxt and Tbx6 is decreased (Ciruna and Rossant, 2001). Moreover, Fgf favors paraxial mesoderm fate at the expense of lateral plate mesoderm since SU5402 rescues the expansion of paraxial domain in mutant mice partially deficient in BMP signaling (Miura et al., 2006). The role of Fgf in paraxial mesoderm specification seems to be conserved between amniotes and anamniotes. However, Fgf cannot induce early myogenic program in mouse but plays a role later in pax3 progenitors of the dermomyotome where it favors the triggering of the myogenic program (Lagha et al., 2008b).

In anamniotes, Fgf plays a peculiar role in the early myogenic program and primitive myotome construction (Figure 7). It is necessary early in medial myogenesis and later in lateral myogenesis both in zebrafish and *Xenopus* (Fürthauer et al., 2004; Groves et al., 2005; Fletcher and Harland, 2008; Della Gaspera et al., 2012b; Osborn et al., 2020). These results suggest that these signaling pathways contribute toward coordinating the myogenic program to other cell behaviors that take place during embryogenesis. In zebrafish, Fgf and Hh signaling cooperate to couple cell movements to muscle differentiation during the apparent somite rotation (Yin et al., 2018). In *Xenopus*, Fgf4 is involved in a community effect that triggers the myogenic program only if several cells interact with each other (Standley et al., 2001).

### 3.2 Wnt Signaling

The Wnt signaling pathway also plays multiple roles in the dorso-mesoderm and the paraxial mesoderm specification in anamniotes. In *Xenopus*, stabilization of β-catenin in the dorsal region of the early embryos is a key event involved in the formation of Nieuwkoop center and Spemann organizer (Kimelman, 2006; Hikasa and Sokol, 2013). As such, β-catenin favors the formation of the entire dorsal region including the paraxial mesoderm. Next, after *Xenopus* midblastula transition, wnt8 has been identified as a ventralizing factor that inhibits the formation of dorsal mesoderm and favors the formation of the presumptive paraxial mesoderm (Christian and Moon, 1993; Hoppler et al., 1996). A similar two-step function has also been identified in the neuro-mesodermal progenitors of the caudal region in zebrafish. First, Wnt promotes the mesoderm fate at the expense of neural fate, and second, Wnt favors the paraxial mesoderm fate at the expense of lateral plate mesoderm (Martin and Kimelman, 2012). Like Fgf, Wnt is essential for the specification of the presumptive paraxial mesoderm in *Xenopus*, but its action on primitive myotome construction is more complex than Fgf (Hoppler et al., 1996; Leyns et al., 1997; Kazanskaya et al., 2004; Fletcher and Harland, 2008). The animal cap assay shows that Wnt is unable to induce mesoderm and muscle cells in these pluripotent cells; nevertheless, during gastrulation, β-catenin directly promotes early Myf5 expression (Christian et al., 1992; Guger and Gumbiner, 1995; Shi et al., 2002). It has also been shown in *Xenopus* embryos that the expression of Wnt target genes could be dependent on other signaling pathways (Christian and Moon, 1993; Nakamura et al., 2016; Kjolby et al., 2019). Therefore, these results suggest that Wnt could be a permissive signal for the instructive Fgf signaling during primitive myotome formation, first in the medial and probably later in the lateral paraxial mesoderm (Shi et al., 2002; Della Gaspera et al., 2012b) (Figure 7).

In amniotes, Wnt/β-catenin also acts early as an organizer inducer and next plays a role in the mesoderm and the paraxial mesoderm specification but without inducing precociously the myogenic program (Yoshikawa et al., 1997; Morkel et al., 2003; Engert et al., 2013; Houston, 2017). Next, during somite formation, the dorsal neural tube and the surface ectoderm express different Wnt genes. Thus, Wnt signaling induces the epithelial state of the somites (Capdevila et al., 1998; Schmidt et al., 2004; Geetha-Loganathan et al., 2006). Wnt signaling is also involved in the dorso-ventral patterning of the somites by promoting the dermomyotome at the expense of the sclerotome (Figure 7). It maintains the epithelial organization of the dermomyotome *via* the transcriptional activation of Tcf15 gene (Wagner et al., 2000; Linker et al., 2005). Moreover, Wnt in cooperation with Hh signaling favors myotome formation in the epaxial somite region (Ikeya and Takada, 1998; Borello et al., 2006). This later function of Wnt could correspond in *Xenopus* to the initiation of myogenesis from dermomyotome (stages 28–30) where the second myogenic wave is initiated at the epaxial and the hypaxial levels of the somite (Della Gaspera et al., 2012a). Although Wnt signaling has been less studied at these stages in *Xenopus*, Wnt11 is one of the Wnt ligands that seems to fulfill a function both in *Xenopus* and amniotes dermomyotome at the same period of development (Garriock and Krieg, 2007; Geetha-Loganathan et al., 2008).

### 3.3 Bmp Signaling

In vertebrates, Bmp4 is expressed in lateral plate mesoderm, in surface ectoderm and in the floor plate. In amniotes, Bmp4 acts on somitic fate at least in two distinct ways: In mice and chick, high Bmp4 concentration specifies the lateral plate mesoderm at the expense of somites (Tonegawa et al., 1997; Miura et al., 2006), at lower concentration, it leads to lateralization of the somites, increasing the expression of Sim1, a lateral marker of somites (Pourquié et al., 1995; Pourquié et al., 1996; Tonegawa et al., 1997; Wijgerde et al., 2005). Bmp4 extends also Pax3 expression of the hypaxial dermomyotome region at the expense of Myod1, keeping cells in an undifferentiated state and inhibiting the differentiation into skeletal striated muscle (Pourquié et al., 1996; Amthor et al., 1999; Kahane et al., 2007) (Figure 7). In the lateral/hypaxial region of somites, Bmp4 also favors the endothelial cell fate at the expense of skeletal striated muscle (Nimmagadda et al., 2005; Ben-Yair and Kalcheim, 2008). In ventro-medial somite region, Bmp4 inhibition by Noggin and Gremlin antagonists is necessary for sclerotome specification (Stafford et al., 2011). In addition, Bmp4 from the dorsal neural tube favors the dorsal sclerotome development or blood vessels cells formation in the medial sclerotome (Monsoro-Burq et al., 1996; Christ et al., 2004; Nimmagadda et al., 2005).

In anamniotes, Bmp from the roof plate and from the hypochord can also act on somites and limits the specification of muscle pioneer cells by Shh in zebrafish (Nguyen-Chi et al., 2012; Keenan and Currie, 2019). Bmp4 has been mainly identified as a lateralizing/ventralizing factor during embryogenesis (Kondo, 2007; Bier and De Robertis, 2015; Zinski et al., 2018). Indeed, in *Xenopus*, the morphants for Bmp4 antagonists (Chordin, Noggin, and Follistatin) are ventralized and the development of all the dorsal structures, i.e., the neural tube, the notochord, and somites is strongly affected (Khokha et al., 2005). In zebrafish, Bmp4 also favors the formation of lateral plate mesoderm at the expense of somites by inducing the expression of Id-HLH genes, which antagonize somitic bHLH such as Mesogenin or Myod1 (Row et al., 2018). Therefore, the main Bmp function in favor of lateral plate mesoderm appears to be conserved between amniotes and anamniotes. Bmp action on dermomyotome could also be an ancestral function since BMP favors dermomyotome development at the expense of the myotome both in urodela species, axolotl, and in zebrafish. (Epperlein et al., 2007; Patterson et al., 2010) (Figure 7).

### 3.4 Hedgehog Signaling

In vertebrates, sonic hedgehog (Shh) is expressed by the notochord and the floor plate and will favor the development of the medial part of somites (Kremnyov et al., 2018). Thus, in amniotes, KO mice for Shh have a major defect in the development of vertebrae, involving Shh in the formation and differentiation of the sclerotome. Pax1, a sclerotome marker, is only transiently expressed in KO mice, whereas the expression of Pax3, a dermomyotome marker, is expanded (Chiang et al., 1996). In chicks and mice, Shh is also necessary for the epaxial myogenesis and Myf5 expression (Teillet et al., 1998; Borycki et al., 1999; Applebaum and Kalcheim, 2015) (Figure 7). In chicks, the transition from the primary myotome formation to the growth phase appears to be related to the ability of the dermomyotome muscle progenitors to respond to hh. In the first phase, they are sensitive and differentiate into muscle fibers, and in the second one, they are insensitive and continue to proliferate (Kahane et al., 2013; Applebaum and Kalcheim, 2015).

In anamniotes, the myotome is the first medial compartment to be formed. In zebrafish, Hh allows the specification of at least three medial cell types in the myotome (Keenan and Currie, 2019). The slow fiber program is induced in adaxial cells by Hh, the pioneer cells, one of the two sub-types of slow fibers, are further specified by Hh, and later, the fast medial fiber fate depends also on Hh (Coutelle et al., 2001; Wolff et al., 2003; Hirsinger et al., 2004). Hh also downregulates the expression of Pax3 and Pax7 in the myogenic progenitors of the dermomyotome and induces myogenic differentiation as in chick (Feng et al., 2006). Moreover, Hh is also necessary for sclerotome development, particularly for the migration of sclerotome cells from the ventral part of somites around notochord, (Ma et al., 2018).

In *Xenopus*, at least two populations of myotome fibers are under the control of Hh. Hh is necessary for the formation of the “adaxial” cells, which give rise to the superficial slow fibers in the caudal region of embryos, and Hh also promotes the formation of the fast muscle fibers in the trunk region (Grimaldi et al., 2004; Martin et al., 2007). Hh also increases the size of the myotome at the expense of Pax3 expression in both epaxial and hypaxial regions of the dermomyotome at stage 28 in *Xenopus* (Martin et al., 2007). The role of Hh on myotome and sclerotome formation seems conserved between amniotes and anamniotes (Figure 7).

In amniotes, Hh is considered as a medializing factor generally opposed to the lateralizing factor Bmp4. In chick, Bmp4 favors the expression of Pax3 in the lateral region at the expense of myogenic differentiation, while Shh has the opposite effect (Pourquié et al., 1996; Amthor et al., 1999). It has been shown that Wnt and Shh pathways can activate Noggin in somites, a secreted protein that neutralizes Bmp4 (Hirsinger et al., 1997). Interestingly, in *Xenopus*, Hh effect begins before stage 20, i.e., before the dermomyotome formation when the somite compartmentalization in *Xenopus* is truly medio-lateral with myotome cells in medial position and MSCs in lateral position (Della Gaspera et al., 2019). Hence, at least two questions could be raised regarding the effect of Shh on medio-lateral patterning in *Xenopus*: 1) Could Shh inhibit the formation of the lateral MSCs population which gives rise to dermomyotome later? 2) Could Bmp4 counteract Shh activity on this cell population? In *Xenopus*, early inhibition of Bmp4 signaling by Noggin decreases satellite cell number at larval stage 45, suggesting that Bmp4 acts precociously on the satellite cell lineage. BMP4 could favor laterally the development of MSCs and/or the dermomyotome at the expense of the primitive myotome (Daughters et al., 2011).

### 3.5 Other Signaling Pathways

Among the other signaling pathways involved in somite compartmentalization and cell fate decisions, the Notch pathway appears as one of the main way to inhibit skeletal myogenesis or maintain myogenic cells in undifferentiated state. Premature somitic myoblasts differentiation is observed in KO mice for Notch ligand Delta1 that causes a deficit in myogenic progenitors and severe muscle hypotrophy (Schuster-Gossler et al., 2007). Furthermore, in chick and mouse somites, Notch pathway plays a role in the acquisition of smooth muscle and endothelial cell fate at the expense of skeletal muscle (Ben-Yair and Kalcheim, 2008; Mayeuf-Louchart et al., 2014). Interestingly, in *Xenopus*, Myod1 activates the Notch pathway during gastrulation, linking thus myogenesis to somitogenesis and/or somite compartmentalization through a potential feedback inhibitory loop (Wittenberger et al., 1999; Maguire et al., 2012).

Retinoic acid, another key signaling factor of somitogenesis, could also play a role in *Xenopus* somite compartmentalization. Indeed, one of its receptors, RARγ, promotes the formation of the primitive myotome during gastrulation, whereas another one, RARβ2 is necessary for the formation of the hypaxial region, which is derived from MSC territory (Janesick et al., 2017 and 2018). However, like other signaling pathways involved in primitive myotome formation, to what extent this function is retained in amniotes remains to be determined. Interestingly, another unidentified signal from the neural plate can extend the primitive myotome domain in *Xenopus* during neurulation, but it is not known if this signal is used to favor the primitive myotome at the expense of MSCs or to favor the paraxial mesoderm at the expense of the lateral plate mesoderm (Mariani et al., 2001).

## 4 Discussion

### 4.1 Developmental Features of Multipotent Somitic Cells

#### 4.1.1 What is the Developmental Origin of Multipotent Somitic Cells in Anamniotes?

In *Xenopus*, morphological techniques allowed to identify the first segmented somites, which appear at mid-neurulation, but failed to define lateral border of paraxial mesoderm at the beginning of neurulation (Hamilton, 1969; Youn et al., 1980; Keller, 2000). Expression studies of Dll4 (Delta-2) and Mesp, two somitogenesis markers, suggest that the paraxial mesoderm extends more laterally than expected (Jen et al., 1997; Hitachi et al., 2009). This lateral region located at LSF expresses somitic markers, Meox2 almost specifically, and Tcf15 highly (Della Gaspera et al., 2012b). Moreover, LSF cells envelop dorsally and ventrally the primitive myotome (Figure 1C) and give rise to both dermomyotome and sclerotome strongly suggesting that LSF is made up of MSCs (Della Gaspera et al., 2012b; Della Gaspera et al., 2019). More refined single-cell RNAseq analyses are yet required to ensure that MSCs are a homogeneous population. The medialward movement of lateral paraxial mesoderm around medial somitic cells has been initially interpreted as a whole tissue unfolding movement of paraxial mesoderm in *Xenopus.* Convergent extension movement of paraxial mesoderm has also been identified at the same time (Hamilton, 1969; Wilson et al., 1989; Harland, 2004). More work is needed to distinguish between cell type-specific migration suggested by MSCs movements and more general tissue movements. Moreover, any convergent extension movement that has been identified in the mesoderm of amphioxus, either during gastrulation or neurulation, suggests that the medialward movement of lateral somitic cells is cell-type-specific (Yasuoka, 2020). In amphioxus, lateral somitic cells also envelop the medial myotome, suggesting that the LSF is the ancestral location of MSCs (Mansfield et al., 2015) (Figure 1B).

**FIGURE 8:**
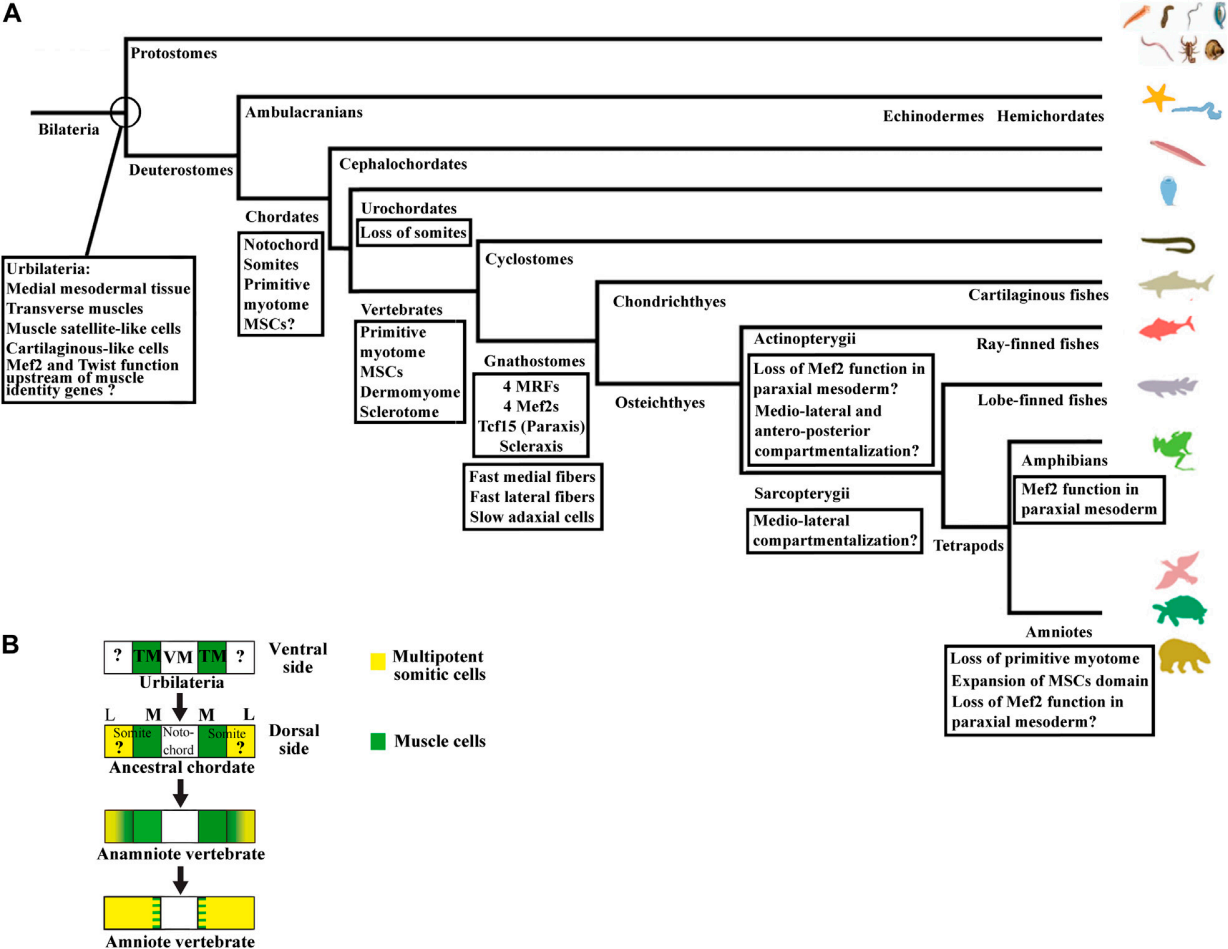
**(A)** Summary of the main changes during the evolutionary history of somites compartmentalization. The last common ancestor of bilaterians, Urbilateria, possesses neither somites nor notochord, but probably transverse muscles and a medial mesodermal tissue according to the axochord hypothesis (Brunnet al, 2015; Yasuoka, 2020). Satellite-like cells and cartilage-like cells are also probably already present in Urbilateria. Regarding the transcription factors expressed in vertebrate somites, results from *Drosophila* and *Xenopus* suggest that Mef2 and Twist could act upstream of muscle identity genes in Urbilateria. The notochord and the somites appear in chordates. The somite is made up of the primitive myotome and probably multipotent progenitors which give rise to satellite cells and muscle-associated tissues ventrally and dorsally. The existence of sclerotome-like cells in cephalochordates suggests that the somitic progenitors can already give rise to specialized connective tissue cells. In vertebrate, the somites compartmentalize mainly into the myotome, the dermomyotome, and the sclerotome. In gnathostome vertebrates, the three populations of slow, lateral fast, and medial fast muscle fibers has been characterized. The genome possesses both Scleraxis and Tcf15 genes, but also four MRFs and four Mef2 genes. The non-conservation of Mef2 function in the paraxial mesoderm and the changes in compartmentalization mode between zebrafish and *Xenopus* raise the question of the origin of these variations. **(B)** Evolution of somite compartmentalization based on axochord hypothesis (Brunet et al., 2015; Yasuoka, 2020). The axochord hypothesis (the axochord in annelids and the notochord in chordate are homologs) proposes that the notochord evolves from a medial mesodermal tissue present in Urbilateria, the last common ancestor of all bilaterians, and suggests that transverse muscles attached to it, could give rise to the primitive myotome in ancestral chordates. The origin of MSCs in Urbilateria is unknown. Proto-MSCs probably already exist in last chordate ancestor. The transition from ancestral chordates to vertebrates allowed MSCs to give rise to all new somite structures, i.e., the dermomyotome, its hypaxial region, and the sclerotome. The transition from anamniote to amniote vertebrates is characterized by expansion of the MSCs domain at the expense of the primitive myotome. The chordate dorso-ventral axis is inverted compared with Urbilateria. Anamniote vertebrate is used in Figure 8B as the somite organization of the extant anamniote vertebrates are considered to be closed to the primitive one. VM, ventro-medial mesodermal tissue; TM, transverse muscle; M, medial somite region; L, lateral somite region.

The compartmentalization mode of the zebrafish shows similarities and differences with the medio-lateral patterning observed in *Xenopus* and amphioxus (Keenan and Currie, 2019). Indeed, the adaxial cells are certainly positioned medially, but the lateral somitic domain patterns in an antero-posterior way before rotating (Figures 3B, C). The anterior somitic cells give rise to the dermomyotome and endotome, the posterior ones give rise to the fast muscle fibers. The zebrafish sclerotome is described as a ventro-medial compartment preferentially originated from the anterior cells (Morin-Kensicki and Eisen, 1997). The somite patterning in an antero-posterior dimension exists in other vertebrates as in the case of the resegmentation of the sclerotome but it is not the main patterning dimension of somite compartmentalization (Williams et al., 2019; Hughes et al., 2009). The initial patterning dimension is medio-lateral in *Xenopus* and dorso-ventral in amniotes. In this regard, the specialized antero-posterior patterning in zebrafish seems to be derived from the ancestral mode that could have appeared in actinopterygian or teleost species (Figure 8A). Highlighting the way in which somites are compartmentalized among chondrichtyans and basal sarcopterygians could inform about the true ancestral mode of compartmentalization. Whatever the case, zebrafish MSCs should exist at some location, probably at an earlier stage of development, and next give rise to both dermomyotome and sclerotome.

#### 4.1.2 What is the Molecular Identity of Multipotent Somitic Cells?

The zebrafish MSCs should also express the same markers as in *Xenopus*, i.e., Tcf15, Meox1 (and/or Meox2), as well as Foxc1 and c2. These genes have been studied in zebrafish, *Xenopus*, and mice. Meox2 is the only Meox genes found in *Xenopus*. The knockdown experiments of *Xenopus* Meox2 showed that Meox2 is necessary for dermomyotome formation, in agreement with mouse Meox2 knockout phenotype (Della Gaspera et al., 2012b). In mice, the double knockout for Meox genes, Meox1 and 2, displays drastic anomalies in all somitic derivatives, including the dermomyotome in which the expression of the dermomyotome marker, Pax3, is severely reduced (Mankoo et al., 2003).

Tcf15 knockdown in *Xenopus* affects dermomyotome formation particularly in the hypaxial domain, as already shown in mice (Della Gaspera et al., 2012b). Indeed, in mice, the Tcf15 knockout shows that Tcf15 is necessary for the development of the hypaxial region of somites and can directly regulate Pax3 and Pax1 expression (Wilson-Rawls et al., 1999; Wilson-Rawls et al., 2004; Takahashi et al., 2007). Moreover, Tcf15 mutant embryos fail to form epithelial somites and fail to maintain the antero-posterior somites polarity (Burgess et al., 1996; Johnson et al., 2001).

Concerning Foxc1 and Foxc2, double knockout mice show that these genes are essential for somite formation (Wilm et al., 2004). Moreover, Foxc2 later promotes somitic endothetial fate at the expense of myogenic fate (Lagha et al., 2009). It can also be noted that in *Xenopus*, animal cap assays indicate that these two Foxc genes inhibit myogenic differentiation (Della Gaspera et al., 2018). Most of these genes expressed in MSCs seem necessary both to establish somite compartmentalization and to oppose myogenic specification or differentiation. So, in anamniotes, these genes could maintain MSCs cell potency at the expense of primitive myotome formation before committing MSCs to a more restricted cell fates in the dermomyotome and the sclerotome.

**FIGURE 9:**
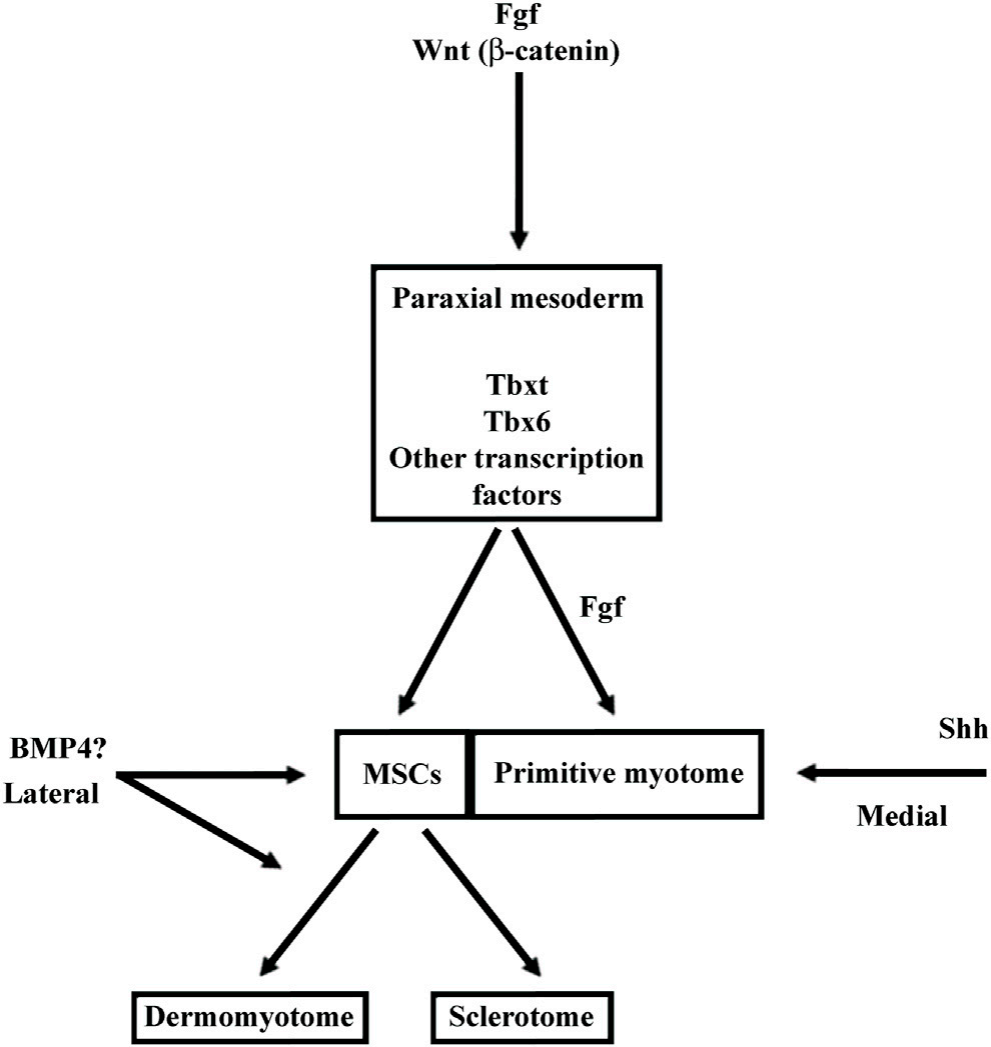
The signaling pathways involved in MSCs and primitive myotome formation in *Xenopus*. In anamniotes and particularly in *Xenopus*, the somite development is characterized by the early and massive myotome formation, and the delayed sclerotome development. The construction of primitive myotome is so early specified that it is interconnected to the dorso-mesoderm induction and the paraxial mesoderm specification. The signaling pathways like Fgf, Wnt and Nodal, involved in the dorso-mesoderm induction and in the paraxial mesoderm specification, also quickly trigger myogenic program leading to the primitive myotome formation (Jones et al., 1995; Wylie et al., 1996; Joseph and Melton, 1997; Fisher et al., 2002; Dorey and Amaya, 2010). In contrary, the amniote myotome formation takes place later after somitogenesis and the same signaling pathways involved earlier in the mesoderm induction and in the paraxial mesoderm specification did not induced myogenic program at the same time (Alev et al., 2013; Kiecker et al., 2016). In *Xenopus*, Fgf and Wnt play a key role in gene expression of the dorso-lateral marginal zone. This region can be considered as the presumptive paraxial mesoderm since it will give rise to somites later. Both Fgf and Wnt also contribute to the expression of Myf5 and Myod1 during the medial myogenic wave in *Xenopus*. Fgf has also been identified as the main inducer of the lateral myogenic wave which occurs later. In the beginning of neurulation, while the MSCs appear at the LSF, sonic hedgehog (Shh) secreted from notochord favors the myotome formation. BMP4 acts during neurulation to favor satellite cells lineage. Since the satellite cells are not already present at this stage, BMP4 would rather promote the MSCs and/or dermomyotome formation (Daughters et al., 2011).

The bipartite subdivision of the somites in *Xenopus* also implies that presomitic progenitors must express a network of transcription factors that makes them competent to engage in any of the two cellular fates, MSCs or primitive myotome. Tbx6 and Tbxt are expressed in these progenitors and are necessary for the development of somites. In Tbx6 knockout mice, only a few anterior somites are formed but show defects in differentiation (Chapman and Papaioannou, 1998). Since Tbxt plays a major role in the development of the notochord, the phenotype of mice mutated for Tbxt is more complex, but shows important defects in posterior somite formation (Chesley, 1935; Wilson et al., 1993). Tbx6 and Tbxt act upstream of the genes expressed by MSCs (Tcf15, Meox1, Meox2, Foxc1, and Foxc2) in mice; Tbx6 and Tbxt are also able to activate myogenic factors in *Xenopus* and zebrafish and contribute to the formation of the primitive myotome and somites (Li et al., 2006; Lou et al., 2006; et Morley et al., 2009; Windner et al., 2012: Gentsch et al., 2013; Osborn et al., 2020). Moreover, Tbx6 already participates in the myogenic program in another group of chordates, the tunicates, which have lost somites during their evolution (Mitani et el, 1999; Yagi et al., 2005). Therefore, Tbx6 and Tbxt, and also other T box genes in anamniotes, play probably a major role in the transcription factors network that acts upstream of MSCs and primitive myotome cells (Gentsch et al., 2013; Amacher et al., 2002) (Figure 9). However, in mice, lineage studies have shown that cells expressing Tbx6 contribute to other mesodermal lineage as in the case of Tbxt (Concepcion et al., 2017; Sadahiro et al., 2018). Neither of these two genes on their own is, therefore, probably sufficient to restrict somitic identity and cell potency to MSCs. In other words, genes conferring somitic identity and cell potency are probably those that expressed more specifically in MSCs like Meox and Tcf15. Mesogenin 1 which acts upstream of Meox and Tcf15 could also play an important role since it is involved in acquisition of presomitic identity in mice (Yoon and Wold, 2000; Chalamalasetty et al., 2014).

### 4.2 Evolution of Multipotent Somitic Cells

#### 4.2.1 Which Cell Type Could be at the Evolutionary Origin of Multipotent Somitic Cells?

In *Xenopus*, MSCs give rise to all the new structures that emerged in vertebrates, the dermomyotome as such, its hypaxial region, and the sclerotome. In amphioxus, the lateral somitic cells give rise to the dorsally external cells and the ventrally sclerotome-like cells suggesting that proto-MSCs already exist in cephalochordates and increase their cell potency capacities at the transition from ancestral chordates to vertebrates (Mansfield et al., 2015; Della Gaspera et al., 2019). More is known about the evolutionary origin of somites and axial mesoderm: it has been proposed that the notochord of chordates, the medial structure that separates the two bilateral rows of somites, could have functional homologies with a medial muscle present in annelids, the axochord (Lauri et al., 2014; Brunet et al., 2015) (Figure 8B). Axochord-like muscle is also found in various groups of protostomes, but the presence of its homolog in deuterostomes is contested (Annona et al., 2015; Inoue and Satoh, 2018; Yasuoka, 2020). Therefore, in the axochord hypothesis, the vertebrate notochord could derive from a medial mesodermal tissue already present in the last common ancestor of all bilaterians, Urbilateria, but its muscular origin is doubtful (Brunet et al., 2015; Yasuoka, 2020). Moreover, Brunet et al. (2015) also suggested that a pair of bilateral transverse muscles, often repeated along the anteroposterior axis in many protostomes, could be at the origin of the primitive myotome due to homologies in location and rotational movements during their formation.

The evolutionary history of muscle tissue is a long one and can be traced back before the ancestor of bilaterians (Steinmetz et al., 2012). Striated muscle types already exist in protostomes and deuterostomes. With the appearance of the rigid notochord in chordates, it has been proposed in the “bottleneck hypothesis” that only few longitudinal striated muscles have been maintained with the development of the axial locomotor system (Thor and Thomas, 2002). Thus, the hypothesis that can be proposed is that Urbilateria has transmitted to chordate somites, both muscle fibers, which have evolved and gave rise to primitive myotome, and some type of progenitors, which have evolved and gave rise to MSCs (Figure 8B). This situation is reminiscent of that encountered during myogenesis in the protostome *Drosophila* (Baylies et al., 1998; Laurichesse and Soler, 2020). Myogenesis takes place in two myogenic waves in this species. During the embryonic phase, the first muscle fibers differentiate, and some progenitors, the adult muscle progenitors (AMPs), remain in an undifferentiated state. Following metamorphosis, embryonic muscles degenerate and AMPs give rise to both adult muscle fibers and muscle satellite cells (Chaturvedi et al., 2017; Boukhatmi and Bray, 2018). Satellite cells have also been described in another protostome, the crustacean species *Parhyale hawaiensis* (Konstantinides and Averof, 2014) and in the mesothelium of amphioxus somites, a muscle-associated tissue, which potentially derived from lateral somitic domain (Somorjai et al., 2012; Mansfield et al., 2015; Yong et al., 2021). Hence, AMP-like cells are probably already present in Urbilateria (Figure 8A). The MSCs have had to acquire the cell potency to give rise to both myogenic and cartilage-like cells, the main derivatives of dermomyotome and sclerotome, and to migrate from a lateral location to a medial one. One possible scenario is that the lineage of AMP-like cells could have evolved and increased their cell potency capacities to also give rise to cartilage-like cells and become MSCs. Yong et al. (2021) suggested that the lateral somitic domain in amphioxus could be first a connective tissue probably associated with muscle and then co-opted genes involved in cartilage and bone development during vertebrate evolution. Alternatively, it has been proposed that sclerotome cells could have evolutionarily derived from the cartilage-like cells of the medial and ventral mesentery that expressed SoxD, SoxE, and Collagen A. These cells have been found in some protostomes and deuterostomes and are probably present in Urbilateria (Brunet and Arendt, 2016; Tarazona et al., 2016) (Figure 8A). Evolutionary origins of MSCs could be the progenitors of cartilage-like cells that have gained the cell potency to give rise to satellite and muscle cells and become MSCs. Cell lineage studies could be used not only in deuterostomes but also in protostomes to identify when a potential lateral mesodermal progenitor that is common to both cell types could be found and, thus, support the evolutionary history of MSCs.

#### 4.2.2 What Evolutionary Processes Could Contribute to Change in the Cell Potency of Multipotent Somitic Cells in Vertebrates?

Among genetic processes that could participate to the evolution of MSCs, gene and genome duplication must be taken into account since it is considered as one of the main forces that had contributed to the evolution of vertebrates (Ohno, 1970; Holland, 1999). The 2R hypothesis states that two rounds (2R) of whole genome duplication have taken place between ancestral chordates and vertebrates (Holland et al., 1999). The first round happens in basal vertebrates and the second round probably after the split between cyclostomes and gnathostomes (Ermakova et al., 2020; Simakov et al., 2020; Nakatani et al., 2021). Genome duplication can increase the number of genes, which evolve next toward neo- or sub-functionalization (Innan and Kondrashov, 2010). Thus, gene and genome duplication could contribute to increase the cell potency capacities of MSCs or MSC-daughter cells, which give rise to multiple somitic lineages in vertebrates (Shimeld and Holland, 2000).

In *Xenopus*, we obtained some results suggesting the functional divergence of paralog genes following gene duplication concerning two gene families involved in somite patterning, the Mef2, and the Twist families of transcription factor. Three events of duplication are hypothesized from the only MEF2 found in protostomes to the four genes found in gnathostomes (Wu et al., 2011). In the Twist family of bHLH transcription factors, Tcf15 and Scleraxis have been involved in the somite patterning. The ancestral Parascleraxis gene is the only gene identified in the cyclostome lamprey whereas the Tcf15 and Scleraxis genes are present in gnathostomes (Freitas et al., 2006). The ancestral function of these genes could be close to the function of the only Mef2 gene in *Drosophila* that acts upstream of muscle identity genes, with some target genes common with Twist, the *Drosophila* ortholog of the vertebrate Twist bHLH family (Sandmann et al., 2006; Sandmann et al., 2007). We and others showed that Mef2d plays a role in paraxial mesoderm formation in *Xenopus* upstream of muscle identity genes (Della Gaspera et al., 2012b; Kolpakova et al., 2013) and that Mef2c marks larval tendon in a later step of somite specification as detailed above (Della Gaspera et al., 2009). Tcf15 and Scleraxis are involved in the same two steps in *Xenopus*, i.e., paraxial mesoderm formation for Tcf15 and larval tendon development for Scleraxis in accordance with their well-established roles in mice somite development (Burgess et al., 1996; Wilson-Rawls et al., 1999; Murchison et al., 2007). Moreover, by gain of function experiments in *Xenopus*, we demonstrated a synergistic effect for Mef2d and Tcf15 on the expression of Pax3, a dermomyotome marker, and for Mef2c and Scleraxis on the expression of Tgfβi and Tenascin c, two components of the tenocyte extracellular matrix (Della Gaspera et al., 2009; Della Gaspera et al., 2012b). From these results, we hypothesize that the ancestral function of Mef2 and parascleraxis has functionally diverged after duplication events: Mef2d and Tcf15 are involved in paraxial mesoderm formation, and Mef2c and Scleraxis in muscle-associated connective tissue formation. However, the function of *Xenopus* Mef2d upstream of muscle identity genes is not conserved in other vertebrate species, zebrafish, and mouse, and could have been lost two times, in actinopterygians or teleosts, and in amniotes (Figure 8A). Alternatively, we cannot exclude that the function of Mef2d in paraxial mesoderm formation could be a specialized function in amphibians, anurans, or the *Xenopus* genus. The identification of target genes common to both transcription factor families in different species could be a way to further highlight the ancestral origin of these functions.

#### 4.2.3 What Evolutionary Processes Could Contribute to the Enlargement of Multipotent Somitic Cells Territory in Amniotes?

During the transition from anamniote to amniote vertebrates, it is the location of MSCs territory that seems to change. In amniotes, the MSCs territory seems to be expanded to the whole somite at the expense of the primitive myotome (Figures 3B and 8B). In line with this view, the markers of the lateral MSCs cells in *Xenopus* (Tcf15, Meox1, and/or Meox2, Foxc1, and/or Foxc2) are expressed in the whole somite in amniotes, whose differentiation into dermomyotome and sclerotome depends on signals emitted by the surrounding tissues. Indeed, the initiation of myogenesis at the epaxial and hypaxial levels of the amniote dermomyotome displays strong homologies with the second myogenic wave observed both in *Xenopus* and axolotl, suggesting that the primitive myotome generated by the first myogenic wave in *Xenopus* has been lost *per se* in amniote (Martin and Harland, 2001; Della Gaspera et al., 2012a; Banfi et al., 2012). However, remnants of the first primitive myogenic wave seem to be present in amniotes. The pioneer cells present in chicks, which appear early at the epithelial somite stage in the medial somite, could be a remnant of the first medial myogenesis of *Xenopus* (Applebaum and Kalcheim, 2015) (Figure 3B). Similarly, the residual myogenesis observed in somites in double KO mice for Pax3 and Pax7, and the existence of the Myf5-dependent and Pax3-independent epaxial myogenesis suggest that the genetic program of the first primitive wave has been partly conserved (Tajbakhsh et al., 1997; Relaix et al., 2005).

To confirm this scenario, it is necessary to identify the mechanisms underlying the expansion of MSCs territory to the whole somite. This expansion could be due to the inhibition of primitive myogenesis. As such, in anamniotes only, early Fgf signaling, Tbxt, and Tbx6 seem to play an important role in the induction of Myod and/or Myf5. This suggests that these processes have been changed in amniotes (Li et al., 2006; Gentsch et al., 2013; Osborn et al., 2020). The expansion of lateral MSCs territory in amniotes could also be facilitated by increasing MSC inductive signals at the expense of the primitive myotome. In this respect, it would be interesting to futher explore the role of the balance between BMP4 and Shh in *Xenopus* as discussed above, since the modification of this balance in favor of BMP4 could lead to the medial expansion of the MSCs territory in amniotes (Figure 9).

### 4.3 Conclusions and Perspectives

It appears that the gain and/or redeployment of genetic programs in MSCs and/or MSC-daughter cells seem to be a key mechanism involved in changes of MSCs cell potency and so, in the somite evolution. The essential role of the LSF as the source of multipotent somitic cells giving rise to different somitic lineages, necessary for the formation of the dorsal musculoskeletal system, has been highlighted in *Xenopus*. The LSF has been defined by Burke and Nowicki (2003) as the changing interface between somites and lateral plate mesoderm which separates the primaxial domain (the musculoskeletal structures comprising somitic cells only) and the abaxial domain (containing muscle or bone of somitic origin associated with connective tissue derived from lateral plate mesoderm). This boundary zone changes during development and has long been recognized as a region where critical signals are exchanged during somite patterning (Nowicki et al., 2003; Durland et al., 2008; Shearman and Burke, 2009). We identified an early developmental and evolutionary event, taking place at the LSF, with the appearance of MSCs. The evolutionary origin of the lateral plate mesoderm can be also traced back to the appearance of chordates as it could be the case for MSCs (Prummel et al., 2019). Moreover, lateral somitic domain in amphioxus also gives rise to the lateral plate mesoderm, raising the question about the causal evolutionary link between the advent of both LSF and MSCs in vertebrates (Mansfield et al., 2015; Yong et al., 2021). In addition, any changes in MSCs cell potency and lineage that occur at the LSF during vertebrate evolution could potentially affect the development of abaxial region. Recently, it has been shown that endothelial cells of the somitic hypaxial region are necessary for the migration of myogenic progenitors into the limb abaxial domain in mice (Yvernogeauet et al, 2012; Mayeuf-Louchart et al., 2016). Colonization by myogenic cells of the vertebrate appendages is observed in gnathostomes but the colonization mode differs between species. In basal gnathostomes, extension of epithelial somites is supposed to be the primitive mode, whereas migration of myogenic progenitors is adopted before the sarcoptegyrian radiation (Neyt et al., 2000; Wotton et al., 2015). So, endothelial cells lineage formation inside somites during vertebrate evolution should be analyzed in parallel to their function in the migration of myogenic progenitors to the abaxial region, in order to explore the potential relationship between MSCs lineage at LSF and the development of the abaxial region.

## Data Availability

The original contributions presented in the study are included in the article/Supplementary Material. Further inquiries can be directed to the corresponding authors.

## References

[B1] Aase-Remedios M. E. Coll-Lladó C. Ferrier D. E. K. (2020). More Than One-To-Four via 2R: Evidence of an Independent Amphioxus Expansion and Two-Gene Ancestral Vertebrate State for MyoD-Related Myogenic Regulatory Factors (MRFs). Mol. Biol. Evol. 37, 2966–2982. 10.1093/molbev/msaa147 32520990 PMC7530620

[B2] Afonin B. Ho M. Gustin J. K. Meloty-Kapella C. Domingo C. R. (2006). Cell Behaviors Associated with Somite Segmentation and Rotation inXenopus Laevis. Dev. Dyn. 235, 3268–3279. 10.1002/dvdy.20979 17048252

[B3] Alev C. Wu Y. Nakaya Y. Sheng G. (2013). Decoupling of Amniote Gastrulation and Streak Formation Reveals a Morphogenetic unity in Vertebrate Mesoderm Induction. Development 140, 2691–2696. 10.1242/dev.094318 23698348

[B4] Amacher S. L. Draper B. W. Summers B. R. Kimmel C. B. (2002). The Zebrafish T-Box Genesno Tailandspadetailare Required for Development of Trunk and Tail Mesoderm and Medial Floor Plate. Development 129, 3311–3323. 10.1242/dev.129.14.3311 12091302

[B5] Ambler C. A. Nowicki J. L. Burke A. C. Bautch V. L. (2001). Assembly of Trunk and Limb Blood Vessels Involves Extensive Migration and Vasculogenesis of Somite-Derived Angioblasts. Dev. Biol. 234, 352–364. 10.1006/dbio.2001.0267 11397005

[B6] Amthor H. Christ B. Patel K. (1999). A Molecular Mechanism Enabling Continuous Embryonic Muscle Growth - A Balance between Proliferation and Differentiation. Development 126, 1041–1053. 10.1242/dev.126.5.1041 9927604

[B7] Annona G. Holland N. D. D’Aniello S. (2015). Evolution of the Notochord. EvoDevo 6, 30. 10.1186/s13227-015-0025-3 26446368 PMC4595329

[B8] Applebaum M. Kalcheim C. (2015). Mechanisms of Myogenic Specification and Patterning. Results Probl. Cel Differ. 56, 77–98. 10.1007/978-3-662-44608-9_4 25344667

[B9] Arnold M. A. Kim Y. Czubryt M. P. Phan D. McAnally J. Qi X. (2007). MEF2C Transcription Factor Controls Chondrocyte Hypertrophy and Bone Development. Dev. Cel. 12, 377–389. 10.1016/j.devcel.2007.02.004 17336904

[B10] Bajard L. Relaix F. Lagha M. Rocancourt D. Daubas P. Buckingham M. E. (2006). A Novel Genetic Hierarchy Functions during Hypaxial Myogenesis: Pax3 Directly Activates Myf5 in Muscle Progenitor Cells in the Limb. Genes Dev. 20, 2450–2464. 10.1101/gad.382806 16951257 PMC1560418

[B11] Banfi S. Monti L. Acquati F. Tettamanti G. Eguileor M. Grimaldi A. (2012). Muscle Development and Differentiation in the Urodele *Ambystoma mexicanum* . Develop. Growth Differ. 54, 489–502. 10.1111/j.1440-169X.2012.01338.x 22519643

[B12] Barresi M. J. F. D'Angelo J. A. Hernández L. P. Devoto S. H. (2001). Distinct Mechanisms Regulate Slow-Muscle Development. Curr. Biol. 11, 1432–1438. 10.1016/S0960-9822(01)00428-6 11566102

[B13] Baylies M. K. Bate M. Gomez M. R. (1998). Myogenesis: A View from Drosophila. Cell 93, 921–927. 10.1016/S0092-8674(00)81198-8 9635422

[B14] Ben-Yair R. Kalcheim C. (2005). Lineage Analysis of the Avian Dermomyotome Sheet Reveals the Existence of Single Cells with Both Dermal and Muscle Progenitor Fates. Development 132, 689–701. 10.1242/dev.01617 15659485

[B15] Ben-Yair R. Kalcheim C. (2008). Notch and Bone Morphogenetic Protein Differentially Act on Dermomyotome Cells to Generate Endothelium, Smooth, and Striated Muscle. J. Cel Biol. 180, 607–618. 10.1083/jcb.200707206 PMC2234248 18268106

[B16] Ben-Yair R. Kahane N. Kalcheim C. (2011). LGN-Dependent Orientation of Cell Divisions in the Dermomyotome Controls Lineage Segregation into Muscle and Dermis. Development 138, 4155–4166. 10.1242/dev.065169 21852400

[B17] Bertrand S. Camasses A. Somorjai I. Belgacem M. R. Chabrol O. Escande M.-L. (2011). Amphioxus FGF Signaling Predicts the Acquisition of Vertebrate Morphological Traits. Proc. Natl. Acad. Sci. 108, 9160–9165. 10.1073/pnas.1014235108 21571634 PMC3107284

[B18] Bertrand S. Aldea D. Oulion S. Subirana L. de Lera A. R. Somorjai I. (2015). Evolution of the Role of RA and FGF Signals in the Control of Somitogenesis in Chordates. PLoS One 10, e0136587. 10.1371/journal.pone.0136587 26371756 PMC4570818

[B19] Bier E. De Robertis E. M. (2015). BMP Gradients: A Paradigm for Morphogen-Mediated Developmental Patterning. Science 348, aaa5838. 10.1126/science.aaa5838 26113727

[B20] Bismuth K. Relaix F. (2010). Genetic Regulation of Skeletal Muscle Development. Exp. Cel Res. 316, 3081–3086. 10.1016/j.yexcr.2010.08.018 20828559

[B21] Bone Q. (1966). On the Function of the Two Types of Myotomal Muscle Fibre in Elasmobranch Fish. J. Mar. Biol. Ass. 46, 321–349. 10.1017/S0025315400027168

[B22] Bone Q. (1978). Locomotor Muscle. Fish. Physiol. 7, 361–424. 10.1016/S1546-5098(08)60168-5

[B23] Bonnin M.-A. Laclef C. Blaise R. Eloy-Trinquet S. Relaix F. Maire P. (2005). Six1 Is Not Involved in Limb Tendon Development, but Is Expressed in Limb Connective Tissue under Shh Regulation. Mech. Dev. 122, 573–585. 10.1016/j.mod.2004.11.005 15804569

[B24] Borello U. Berarducci B. Murphy P. Bajard L. Buffa V. Piccolo S. (2006). The Wnt/β-Catenin Pathway Regulates Gli-mediatedMyf5expression during Somitogenesis. Development 133, 3723–3732. 10.1242/dev.02517 16936075

[B25] Borycki A. G. Brunk B. Tajbakhsh S. Buckingham M. Chiang C. Emerson C. P. (1999). Sonic Hedgehog Controls Epaxial Muscle Determination through Myf5 Activation. Development 126, 4053–4063. 10.1242/dev.126.18.4053 10457014

[B26] Boudjelida H. Muntz L. (1987). Multinucleation during Myogenesis of the Myotome of *Xenopus laevis*: A Qualitative Study. Development 101, 583–590. 10.1242/dev.101.3.583 3502996

[B27] Boukhatmi H. Bray S. (2018). A Population of Adult Satellite-like Cells in Drosophila Is Maintained through a Switch in RNA-Isoforms. Elife 7, e35954. 10.7554/eLife.35954 29629869 PMC5919756

[B28] Brand-Saberi B. Christ B. (2000). Evolution and Development of Distinct Cell Lineages Derived from Somites. Curr. Top. Dev. Biol. 48, 1–42. 10.1016/s0070-2153(08)60753-x 10635456

[B29] Braun T. Buschhausen-Denker G. Bober E. Tannich E. Arnold H. H. (1989). A Novel Human Muscle Factor Related to but Distinct from MyoD1 Induces Myogenic Conversion in 10T1/2 Fibroblasts. EMBO J. 8, 701–709. 10.1002/j.1460-2075.1989.tb03429.x 2721498 PMC400865

[B30] Brent A. E. Tabin C. J. (2004). FGF Acts Directly on the Somitic Tendon Progenitors through the Ets Transcription Factors Pea3 and Erm to Regulate Scleraxis Expression. Development 131, 3885–3896. 10.1242/dev.01275 15253939

[B31] Brent A. E. Schweitzer R. Tabin C. J. (2003). A Somitic Compartment of Tendon Progenitors. Cell 113, 235–248. 10.1016/S0092-8674(03)00268-X 12705871

[B32] Brent A. E. Braun T. Tabin C. J. (2005). Genetic Analysis of Interactions between the Somitic Muscle, Cartilage and Tendon Cell Lineages during Mouse Development. Development 132, 515–528. 10.1242/dev.01605 15634692

[B33] Bricard Y. Rallière C. Lebret V. Lefevre F. Rescan P.-Y. (2014). Early Fish Myoseptal Cells: Insights from the trout and Relationships with Amniote Axial Tenocytes. PLoS One 9, e91876. 10.1371/journal.pone.0091876 24622730 PMC3951490

[B34] Brunet T. Arendt D. (2016). Animal Evolution: The Hard Problem of Cartilage Origins. Curr. Biol. 26, R685–R688. 10.1016/j.cub.2016.05.062 27458918

[B35] Brunet T. Lauri A. Arendt D. (2015). Did the Notochord Evolve from an Ancient Axial Muscle? the Axochord Hypothesis. BioEssays 37, 836–850. 10.1002/bies.201500027 26172338 PMC5054868

[B36] Buckingham M. Vincent S. D. (2009). Distinct and Dynamic Myogenic Populations in the Vertebrate Embryo. Curr. Opin. Genet. Dev. 19, 444–453. 10.1016/j.gde.2009.08.001 19762225

[B37] Buckingham M. (2001). Skeletal Muscle Formation in Vertebrates. Curr. Opin. Genet. Dev. 11, 440–448. 10.1016/S0959-437X(00)00215-X 11448631

[B38] Buckingham M. (2017). Gene Regulatory Networks and Cell Lineages that Underlie the Formation of Skeletal Muscle. Proc. Natl. Acad. Sci. USA 114, 5830–5837. PMID: 28584083; PMCID: PMC5468682. 10.1073/pnas.1610605114 28584083 PMC5468682

[B39] Burgess R. Rawls A. Brown D. Bradley A. Olson E. N. (1996). Requirement of the Paraxis Gene for Somite Formation and Musculoskeletal Patterning. Nature 384, 570–573. 10.1038/384570a0 8955271

[B40] Burke A. C. Nowicki J. L. (2003). A New View of Patterning Domains in the Vertebrate Mesoderm. Dev. Cel. 4, 159–165. 10.1016/S1534-5807(03)00033-9 12586060

[B41] Capdevila J. Tabin C. Johnson R. L. (1998). Control of Dorsoventral Somite Patterning by Wnt-1 and β-Catenin. Dev. Biol. 193, 182–194. 10.1006/dbio.1997.8806 9473323

[B42] Chal J. Pourquié O. (2017). Making Muscle: Skeletal Myogenesis *In Vivo* and *In Vitro* . Development 144, 2104–2122. 10.1242/dev.151035 28634270

[B43] Chalamalasetty R. B. Garriock R. J. Dunty W. C. Kennedy M. W. Jailwala P. Si H. (2014). Mesogenin 1 Is a Master Regulator of Paraxial Presomitic Mesoderm Differentiation. Development 141, 4285–4297. 10.1242/dev.110908 25371364 PMC4302905

[B44] Chanoine C. Hardy S. (2003). Xenopus Muscle Development: From Primary to Secondary Myogenesis. Dev. Dyn. 226, 12–23. 10.1002/dvdy.10206 12508220

[B45] Chapman D. L. Papaioannou V. E. (1998). Three Neural Tubes in Mouse Embryos with Mutations in the T-Box Gene Tbx6. Nature 391, 695–697. 10.1038/35624 9490412

[B46] Charpentier M. S. Tandon P. Trincot C. E. Koutleva E. K. Conlon F. L. (2015). A Distinct Mechanism of Vascular Lumen Formation in Xenopus Requires EGFL7. PLoS One 10, e0116086–23. 10.1371/journal.pone.0116086 25705891 PMC4338030

[B47] Chaturvedi D. Reichert H. Gunage R. D. VijayRaghavan K. (2017). Identification and Functional Characterization of Muscle Satellite Cells in Drosophila. Elife 6, 1–25. 10.7554/eLife.30107 PMC5681227 29072161

[B48] Chen J. W. Galloway J. L. (2014). The Development of Zebrafish Tendon and Ligament Progenitors. Development 141, 2035–2045. 10.1242/dev.104067 24803652 PMC4011085

[B49] Chen Y. Lin G. Slack J. M. W. (2006). Control of Muscle Regeneration in the Xenopus Tadpole Tail by Pax7. Development 133, 2303–2313. 10.1242/dev.02397 16687446

[B50] Chesley P. (1935). Development of the Short-Tailed Mutant in the House Mouse. J. Exp. Zool. 70, 429–459. 10.1002/jez.1400700306

[B51] Chiang C. Litingtung Y. Lee E. Young K. E. Corden J. L. Westphal H. (1996). Cyclopia and Defective Axial Patterning in Mice Lacking Sonic Hedgehog Gene Function. Nature 383, 407–413. 10.1038/383407a0 8837770

[B52] Christ B. Ordahl C. P. (1995). Early Stages of Chick Somite Development. Anat. Embryol. 191, 381–396. 10.1007/BF00304424 7625610

[B53] Christ B. Huang R. Scaal M. (2004). Formation and Differentiation of the Avian Sclerotome. Anat. Embryol. 208, 333–350. 10.1007/s00429-004-0408-z 15309628

[B54] Christ B. Huang R. Scaal M. (2007). Amniote Somite Derivatives. Dev. Dyn. 236, 2382–2396. 10.1002/dvdy.21189 17557304

[B55] Christian J. L. Moon R. T. (1993). Interactions between Xwnt-8 and Spemann Organizer Signaling Pathways Generate Dorsoventral Pattern in the Embryonic Mesoderm of Xenopus. Genes Dev. 7, 13–28. 10.1101/gad.7.1.13 8422982

[B56] Christian J. L. Olson D. J. Moon R. T. (1992). Xwnt-8 Modifies the Character of Mesoderm Induced by bFGF in Isolated Xenopus Ectoderm. EMBO J. 11, 33–41. 10.1002/j.1460-2075.1992.tb05024.x 1740111 PMC556422

[B57] Chung H.-M. Neff A. W. Malacinski G. M. (1989). Autonomous Death of Amphibian (*Xenopus laevis*) Cranial Myotomes. J. Exp. Zool. 251, 290–299. 10.1002/jez.1402510305 2769206

[B58] Ciau-Uitz A. Patient R. (2016). The Embryonic Origins and Genetic Programming of Emerging Haematopoietic Stem Cells. FEBS Lett. 590, 4002–4015. 10.1002/1873-3468.12363 27531714

[B59] Ciau-Uitz A. Walmsley M. Patient R. (2000). Distinct Origins of Adult and Embryonic Blood in Xenopus. Cell 102, 787–796. 10.1016/S0092-8674(00)00067-2 11030622

[B60] Ciau-Uitz A. Liu F. Patient R. (2010). Genetic Control of Hematopoietic Development in Xenopus and Zebrafish. Int. J. Dev. Biol. 54, 1139–1149. 10.1387/ijdb.093055ac 20711991

[B61] Ciruna B. Rossant J. (2001). FGF Signaling Regulates Mesoderm Cell Fate Specification and Morphogenetic Movement at the Primitive Streak. Dev. Cel. 1, 37–49. 10.1016/S1534-5807(01)00017-X 11703922

[B62] Cleaver O. Krieg P. A. (1998). VEGF Mediates Angioblast Migration during Development of the Dorsal Aorta in Xenopus. Development 125, 3905–3914. 10.1242/dev.125.19.3905 9729498

[B63] Cleaver O. Tonissen K. F. Saha M. S. Krieg P. A. (1997). Neovascularization of theXenopus Embryo. Dev. Dyn. 210, 66–77. 10.1002/(sici)1097-0177(199709)210:1<66:aid-aja7>3.0.co;2-# 9286596

[B64] Comai G. Sambasivan R. Gopalakrishnan S. Tajbakhsh S. (2014). Variations in the Efficiency of Lineage Marking and Ablation Confound Distinctions between Myogenic Cell Populations. Dev. Cel. 31, 654–667. 10.1016/j.devcel.2014.11.005 25490270

[B331] Concepcion D. Washkowitz A. J. DeSantis A. Ogea P. Yang J. I. Douglas N. C. (2017). Cell Lineage Of Timed Cohorts Of Tbx6-Expressing Cells In Wild-Type And Tbx6 Mutant Embryos. Biol. Open. 6 (7), 1065–1073. 10.1242/bio.026203 28606934 PMC5550921

[B65] Conerly M. L. Yao Z. Zhong J. W. Groudine M. Tapscott S. J. (2016). Distinct Activities of Myf5 and MyoD Indicate Separate Roles in Skeletal Muscle Lineage Specification and Differentiation. Dev. Cel. 36, 375–385. 10.1016/j.devcel.2016.01.021 PMC4769793 26906734

[B66] Coutelle O. Blagden C. S. Hampson R. Halai C. Rigby P. W. J. Hughes S. M. (2001). Hedgehog Signalling Is Required for Maintenance of Myf5 and myoD Expression and Timely Terminal Differentiation in Zebrafish Adaxial Myogenesis. Dev. Biol. 236, 136–150. 10.1006/dbio.2001.0193 11456450

[B67] Daughters R. S. Chen Y. Slack J. M. W. (2011). Origin of Muscle Satellite Cells in the Xenopus Embryo. Development 138, 821–830. 10.1242/dev.056481 21270051 PMC3035087

[B68] Davis R. L. Weintraub H. Lassar A. B. (1987). Expression of a Single Transfected cDNA Converts Fibroblasts to Myoblasts. Cell 51, 987–1000. 10.1016/0092-8674(87)90585-X 3690668

[B69] De Val S. Anderson J. P. Heidt A. B. Khiem D. Xu S.-M. Black B. L. (2004). Mef2c Is Activated Directly by Ets Transcription Factors through an Evolutionarily Conserved Endothelial Cell-specific Enhancer. Dev. Biol. 275, 424–434. 10.1016/j.ydbio.2004.08.016 15501228

[B70] Della Gaspera B. Armand A.-S. Sequeira I. Lecolle S. Gallien C. L. Charbonnier F. (2009). The Xenopus MEF2 Gene Family: Evidence of a Role for XMEF2C in Larval Tendon Development. Dev. Biol. 328, 392–402. 10.1016/j.ydbio.2009.01.039 19389348

[B71] Della Gaspera B. Armand A.-S. Sequeira I. Chesneau A. Mazabraud A. Lécolle S. (2012a). Myogenic Waves and Myogenic Programs during Xenopus Embryonic Myogenesis. Dev. Dyn. 241, 995–1007. 10.1002/dvdy.23780 22434732

[B72] Della Gaspera B. Armand A.-S. Lecolle S. Charbonnier F. Chanoine C. (2012b). Mef2d Acts Upstream of Muscle Identity Genes and Couples Lateral Myogenesis to Dermomyotome Formation in *Xenopus laevis* . PLoS One 7, e52359. 10.1371/journal.pone.0052359 23300648 PMC3534117

[B73] Della Gaspera B. Chesneau A. Weill L. Charbonnier F. Chanoine C. (2018). Xenopus SOX5 Enhances Myogenic Transcription Indirectly through Transrepression. Dev. Biol. 442, 262–275. 10.1016/j.ydbio.2018.07.025 30071218

[B74] Della Gaspera B. Mateus A. Andéol Y. Weill L. Charbonnier F. Chanoine C. (2019). Lineage Tracing of Sclerotome Cells in Amphibian Reveals that Multipotent Somitic Cells Originate from Lateral Somitic Frontier. Dev. Biol. 453, 11–18. 10.1016/j.ydbio.2019.05.009 31128088

[B75] Devoto S. H. Melançon E. Eisen J. S. Westerfield M. (1996). Identification of Separate Slow and Fast Muscle Precursor Cells *In Vivo*, Prior to Somite Formation. Development 122, 3371–3380. 10.1242/dev.122.11.3371 8951054

[B76] Devoto S. H. Stoiber W. Hammond C. L. Steinbacher P. Haslett J. R. Barresi M. J. F. (2006). Erratum: Generality of Vertebrate Development Patterns: Evidence for a Dermomyotome in Fish. Evol. Dev. 8, 239. 10.1111/j.1525-142X.2006.00802001.x PMC3360970 16409387

[B77] Dichmann D. S. Walentek P. Harland R. M. (2015). The Alternative Splicing Regulator Tra2b Is Required for Somitogenesis and Regulates Splicing of an Inhibitory Wnt11b Isoform. Cel. Rep. 10, 527–536. 10.1016/j.celrep.2014.12.046 PMC4351874 25620705

[B78] Dorey K. Amaya E. (2010). FGF Signalling: Diverse Roles during Early Vertebrate Embryogenesis. Development 137, 3731–3742. 10.1242/dev.037689 20978071 PMC3747497

[B317] Dequéant M. L. Pourquié O. (2008). Segmental Patterning Of The Vertebrate Embryonic Axis. Nat. Rev. Genet. 9 (5), 370–382. 10.1038/nrg2320 18414404

[B79] Durland J. L. Sferlazzo M. Logan M. Burke A. C. (2008). Visualizing the Lateral Somitic Frontier in the Prx1Cre Transgenic Mouse. J. Anat. 212, 590–602. 10.1111/j.1469-7580.2008.00879.x 18430087 PMC2409079

[B80] El-Hodiri H. Bhatia-Dey N. Kenyon K. Ault K. Dirksen M. Jamrich M. (2001). Fox (Forkhead) Genes Are Involved in the Dorso-Ventral Patterning of the Xenopus Mesoderm. Int. J. Dev. Biol. 45, 265–271. 10.1387/ijdb.11291856 11291856

[B81] Engert S. Burtscher I. Liao W. P. Dulev S. Schotta G. Lickert H. (2013). Wnt/β-Catenin Signalling Regulates Sox17 Expression and Is Essential for Organizer and Endoderm Formation in the Mouse. Development 140, 3128–3138. 10.1242/dev.088765 23824574

[B82] Epperlein H. H. Vichev K. Heidrich F. M. Kurth T. (2007). BMP-4 and Noggin Signaling Modulate Dorsal Fin and Somite Development in the Axolotl Trunk. Dev. Dyn. 236, 2464–2474. 10.1002/dvdy.21247 17654602

[B83] Ermakova G. V. Kucheryavyy A. V. Zaraisky A. G. Bayramov A. V. (2020). Discovery of Four Noggin Genes in Lampreys Suggests Two Rounds of Ancient Genome Duplication. Commun. Biol. 3, 1–13. 10.1038/s42003-020-01234-3 32913324 PMC7483449

[B325] Esner M. Meilhac S. M. Relaix F. Nicolas J. F. Cossu G. Buckingham M. E. (2006). Smooth Muscle Of The Dorsal Aorta Shares A Common Clonal Origin With Skeletal Muscle Of The Myotome. Development 133 (4), 737–749. 10.1242/dev.02226 16436625

[B84] Etchevers H. C. Vincent C. Le Douarin N. M. Couly G. F. (2001). The Cephalic Neural Crest Provides Pericytes and Smooth Muscle Cells to All Blood Vessels of the Face and Forebrain. Development 128, 1059–1068. 10.1242/dev.128.7.1059 11245571

[B85] Fan S.-Y. de Sá R. O. Radice G. P. de Sa R. O. (2001). A Common Pattern of Somite Cell Rotation in Three Species of Pipidae. J. Herpetol. 35, 114–116. 10.2307/1566031

[B86] Feng X. Adiarte E. G. Devoto S. H. (2006). Hedgehog Acts Directly on the Zebrafish Dermomyotome to Promote Myogenic Differentiation. Dev. Biol. 300, 736–746. 10.1016/j.ydbio.2006.08.056 17046741

[B87] Fisher M. E. Isaacs H. V. Pownall M. E. (2002). eFGF Is Required for Activation of XmyoD Expression in the Myogenic Cell Lineage of *Xenopus laevis* . Development 129, 1307–1315. 10.1242/dev.129.6.1307 11880340

[B326] Fletcher R. B. Baker J. C. Harland R. M. (2006). FGF8 Spliceforms Mediate Early Mesoderm And Posterior Neural Tissue Formation In Xenopus. Development 133 (9), 1703–1714. 10.1242/dev.02342 16554360

[B88] Fletcher R. B. Harland R. M. (2008). The Role of FGF Signaling in the Establishment and Maintenance of Mesodermal Gene Expression inXenopus. Dev. Dyn. 237, 1243–1254. 10.1002/dvdy.21517 18386826 PMC3000043

[B89] Flood P. R. (1967). Structure of the Segmental Trunk Muscle in Amphioxus. Z. Zellforsch. 84, 389–416. 10.1007/bf00334754 4881202

[B90] Frank D. Harland R. M. (1991). Transient Expression of XMyoD in Non-Somitic Mesoderm of Xenopus Gastrulae. Development 113, 1387–1393. 10.1242/dev.113.4.1387 1667381

[B91] Freitas R. Zhang G. Cohn M. J. (2006). Evidence that Mechanisms of Fin Development Evolved in the Midline of Early Vertebrates. Nature 442, 1033–1037. 10.1038/nature04984 16878142

[B92] Fürthauer M. Van Celst J. Thisse C. Thisse B. (2004). Fgf Signalling Controls the Dorsoventral Patterning of the Zebrafish Embryo. Development 131, 2853–2864. 10.1242/dev.01156 15151985

[B93] Ganassi M. Badodi S. Polacchini A. Baruffaldi F. Battini R. Hughes S. M. (2014). Distinct Functions of Alternatively Spliced Isoforms Encoded by Zebrafish Mef2ca and Mef2cb. Biochim. Biophys. Acta (Bba) - Gene Regul. Mech. 1839, 559–570. 10.1016/j.bbagrm.2014.05.003 PMC4064114 24844180

[B94] Ganassi M. Badodi S. Ortuste Quiroga H. P. Zammit P. S. Hinits Y. Hughes S. M. (2018). Myogenin Promotes Myocyte Fusion to Balance Fibre Number and Size. Nat. Commun. 9, 4232. 10.1038/s41467-018-06583-6 30315160 PMC6185967

[B95] Garriock R. J. Krieg P. A. (2007). Wnt11-R Signaling Regulates a Calcium Sensitive EMT Event Essential for Dorsal Fin Development of Xenopus. Dev. Biol. 304, 127–140. 10.1016/j.ydbio.2006.12.020 17240368 PMC1905145

[B327] Geetha-Loganathan P. Nimmagadda S. Scaal M. Huang R. Christ B. (2008). Wnt Signaling In Somite Development. Ann Anat. 190 (3), 208–222. 10.1016/j.aanat.2007.12.003 18417332

[B96] Geetha-Loganathan P. Nimmagadda S. Huang R. Christ B. Scaal M. (2006). Regulation of Ectodermal Wnt6 Expression by the Neural Tube Is Transduced by Dermomyotomal Wnt11: A Mechanism of Dermomyotomal Lip Sustainment. Development 133, 2897–2904. 10.1242/dev.02464 16818447

[B97] Gentsch G. E. Owens N. D. L. Martin S. R. Piccinelli P. Faial T. Trotter M. W. B. (2013). *In Vivo* T-Box Transcription Factor Profiling Reveals Joint Regulation of Embryonic Neuromesodermal Bipotency. Cel Rep. 4, 1185–1196. 10.1016/j.celrep.2013.08.012 PMC3791401 24055059

[B98] Green Y. S. Vetter M. L. (2011). EBF Proteins Participate in Transcriptional Regulation of Xenopus Muscle Development. Dev. Biol. 358, 240–250. 10.1016/j.ydbio.2011.07.034 21839736 PMC3352962

[B99] Grenier J. Teillet M.-A. Grifone R. Kelly R. G. Duprez D. (2009). Relationship between Neural Crest Cells and Cranial Mesoderm during Head Muscle Development. PLoS One 4, e4381. 10.1371/journal.pone.0004381 19198652 PMC2634972

[B100] Grifone R. Demignon J. Houbron C. Souil E. Niro C. Seller M. J. (2005). Six1 and Six4 Homeoproteins Are Required for Pax3 and Mrf Expression during Myogenesis in the Mouse Embryo. Development 132, 2235–2249. 10.1242/dev.01773 15788460

[B101] Grimaldi A. Tettamanti G. Martin B. L. Gaffield W. Pownall M. E. Hughes S. M. (2004). Hedgehog Regulation of Superficial Slow Muscle Fibres inXenopusand the Evolution of Tetrapod Trunk Myogenesis. Development 131, 3249–3262. 10.1242/dev.01194 15201218

[B102] Gros J. Scaal M. Marcelle C. (2004). A Two-Step Mechanism for Myotome Formation in Chick. Dev. Cel. 6, 875–882. 10.1016/j.devcel.2004.05.006 15177035

[B103] Gros J. Manceau M. Thomé V. Marcelle C. (2005). A Common Somitic Origin for Embryonic Muscle Progenitors and Satellite Cells. Nature 435, 954–958. 10.1038/nature03572 15843802

[B104] Groves J. A. Hammond C. L. Hughes S. M. (2005). Fgf8 Drives Myogenic Progression of a Novel Lateral Fast Muscle Fibre Population in Zebrafish. Development 132, 4211–4222. 10.1242/dev.01958 16120642

[B105] Guger K. A. Gumbiner B. M. (1995). β-Catenin Has Wnt-like Activity and Mimics the Nieuwkoop Signaling Center inXenopusDorsal-Ventral Patterning. Dev. Biol. 172, 115–125. 10.1006/dbio.1995.0009 7589792

[B106] Gurdon J. B. Brennan S. Fairman S. Mohun T. J. (1984). Transcription of Muscle-specific Actin Genes in Early xenopus Development: Nuclear Transplantation and Cell Dissociation. Cell 38, 691–700. 10.1016/0092-8674(84)90264-2 6488316

[B107] Haldar M. Karan G. Tvrdik P. Capecchi M. R. (2008). Two Cell Lineages, Myf5 and Myf5-independent, Participate in Mouse Skeletal Myogenesis. Dev. Cel 14, 437–445. 10.1016/j.devcel.2008.01.002 PMC2917991 18331721

[B108] Hamilton L. (1969). The Formation of Somites in Xenopus. J. Embryol. Exp. Morphol. 22, 253–264. 10.1242/dev.22.2.253 5361556

[B109] Hammond K. L. Baxendale S. McCauley D. W. Ingham P. W. Whitfield T. T. (2009). Expression Ofpatched, Prdm1andengrailedin the Lamprey Somite Reveals Conserved Responses to Hedgehog Signaling. Evol. Dev. 11, 27–40. 10.1111/j.1525-142X.2008.00300.x 19196331

[B110] Harland R. M. (2004). “Dorsoventral Patterning of the Mesoderm,” in Gastrulation from Cells to Embryo. Editor Stern C. (New York: Cold Spring Harbor Laboratory Press), 373–388.

[B111] Hasty P. Bradley A. Morris J. H. Edmondson D. G. Venuti J. M. Olson E. N. (1993). Muscle Deficiency and Neonatal Death in Mice with a Targeted Mutation in the Myogenin Gene. Nature 364, 501–506. 10.1038/364501a0 8393145

[B112] Havis E. Bonnin M.-A. Olivera-Martinez I. Nazaret N. Ruggiu M. Weibel J. (2014). Transcriptomic Analysis of Mouse Limb Tendon Cells during Development. Development 141, 3683–3696. 10.1242/dev.108654 25249460

[B114] Hernández-Hernández J. M. García-González E. G. Brun C. E. Rudnicki M. A. (2017). The Myogenic Regulatory Factors, Determinants of Muscle Development, Cell Identity and Regeneration. Semin. Cel Dev. Biol. 72, 10–18. 10.1016/j.semcdb.2017.11.010 PMC5723221 29127045

[B115] Hikasa H. Sokol S. Y. (2013). Wnt Signaling in Vertebrate axis Specification. Cold Spring Harbor Perspect. Biol. 5, a007955. 10.1101/cshperspect.a007955 PMC3579404 22914799

[B116] Hinits Y. Osborn D. P. S. Hughes S. M. (2009). Differential Requirements for Myogenic Regulatory Factors Distinguish Medial and Lateral Somitic, Cranial and Fin Muscle Fibre Populations. Development 136, 403–414. 10.1242/dev.028019 19141670 PMC2687589

[B117] Hinits Y. Williams V. C. Sweetman D. Donn T. M. Ma T. P. Moens C. B. (2011). Defective Cranial Skeletal Development, Larval Lethality and Haploinsufficiency in Myod Mutant Zebrafish. Dev. Biol. 358, 102–112. 10.1016/j.ydbio.2011.07.015 21798255 PMC3360969

[B118] Hirsinger E. Duprez D. Jouve C. Malapert P. Cooke J. Pourquié O. (1997). Noggin Acts Downstream of Wnt and Sonic Hedgehog to Antagonize BMP4 in Avian Somite Patterning. Development 124, 4605–4614. 10.1242/dev.124.22.4605 9409677

[B119] Hirsinger E. Stellabotte F. Devoto S. H. Westerfield M. (2004). Hedgehog Signaling Is Required for Commitment but Not Initial Induction of Slow Muscle Precursors. Dev. Biol. 275, 143–157. 10.1016/j.ydbio.2004.07.030 15464578

[B120] Hirst C. E. Marcelle C. (2015). The Avian Embryo as a Model System for Skeletal Myogenesis. Results Probl. Cel Differ. 56, 99–122. 10.1007/978-3-662-44608-9_5 25344668

[B121] Hitachi K. Kondow A. Danno H. Nishimura Y. Okabayashi K. Asashima M. (2009). Molecular Analyses of *Xenopus laevis* Mesp-Related Genes. Integr. Zool. 4, 387–394. 10.1111/j.1749-4877.2009.00110.x 21392310

[B122] Hogan B. M. Schulte-Merker S. (2017). How to Plumb a Pisces: Understanding Vascular Development and Disease Using Zebrafish Embryos. Dev. Cel 42, 567–583. 10.1016/j.devcel.2017.08.015 28950100

[B123] Holland L. Z. Schubert M. Kozmik Z. Holland N. D. (1999). AmphiPax3/7, an Amphioxus Paired Box Gene: Insights into Chordate Myogenesis, Neurogenesis, and the Possible Evolutionary Precursor of Definitive Vertebrate Neural Crest. Evol. Dev. 1, 153–165. 10.1046/j.1525-142x.1999.99019.x 11324100

[B124] Holland L. Z. (1996). Muscle Development in Amphioxus: Morphology, Biochemistry, and Molecular Biology. Isr. J. Zool. 42, 37–41. 10.1080/00212210.1996.10688883

[B125] Holland P. W. H. (1999). Gene Duplication: Past, Present and Future. Semin. Cel Dev. Biol. 10, 541–547. 10.1006/scdb.1999.0335 10597638

[B126] Hollway G. E. Bryson-Richardson R. J. Berger S. Cole N. J. Hall T. E. Currie P. D. (2007). Whole-somite Rotation Generates Muscle Progenitor Cell Compartments in the Developing Zebrafish Embryo. Dev. Cel. 12, 207–219. 10.1016/j.devcel.2007.01.001 17276339

[B127] Hoppler S. Brown J. D. Moon R. T. (1996). Expression of a Dominant-Negative Wnt Blocks Induction of MyoD in Xenopus Embryos. Genes Dev. 10, 2805–2817. 10.1101/gad.10.21.2805 8946920

[B128] Hopwood N. D. Pluck A. Gurdon J. B. (1989). MyoD Expression in the Forming Somites Is an Early Response to Mesoderm Induction in Xenopus Embryos. Trends Genet. 5, 363. 10.1016/0168-9525(89)90157-1 PMC401488 2555164

[B129] Hopwood N. D. Pluck A. Gurdon J. B. (1991). Xenopus Myf-5 marks Early Muscle Cells and Can Activate Muscle Genes Ectopically in Early Embryos. Development 111, 551–560. 10.1242/dev.111.2.551 1716555

[B130] Hopwood N. D. Pluck A. Gurdon J. B. Dilworth S. M. (1992). Expression of XMyoD Protein in Early *Xenopus laevis* Embryos. Development 114, 31–38. 10.1242/dev.114.1.31 1315678

[B131] Houston D. W. (2017). Vertebrate Axial Patterning: From Egg to Asymmetry. Adv. Exp. Med. Biol. 953, 209–306. 10.1007/978-3-319-46095-6_6 27975274 PMC6550305

[B132] Huang A. H. Watson S. S. Wang L. Baker B. Akiyama H. Brigande J. V. (2019). Requirement for Scleraxis in the Recruitment of Mesenchymal Progenitors during Embryonic Tendon Elongation. Development 146, 1–8. 10.1242/dev.182782 PMC6826031 31540914

[B133] Hubaud A. Pourquié O. (2014). Signalling Dynamics in Vertebrate Segmentation. Nat. Rev. Mol. Cel Biol. 15, 709–721. 10.1038/nrm3891 25335437

[B329] Hughes D. S. Keynes R. J. Tannahill D. (2009). Extensive Molecular Differences Between Anterior- And Posterior-Half-Sclerotomes Underlie Somite Polarity And Spinal Nerve Segmentation. BMC Dev Biol. 9, 30. 10.1186/1471-213X-9-30 19463158 PMC2693541

[B134] Ikeya M. Takada S. (1998). Wnt Signaling from the Dorsal Neural Tube Is Required for the Formation of the Medial Dermomyotome. Development 125, 4969–4976. 10.1242/dev.125.24.4969 9811581

[B333] Innan H. Kondrashov F. (2010). The Evolution Of Gene Duplications: Classifying And Distinguishing Between Models. Nat. Rev. Genet. 11 (2), 97–108. 10.1038/nrg2689 20051986

[B135] Inoue J. Satoh N. (2018). Deuterostome Genomics: Lineage-Specific Protein Expansions that Enabled Chordate Muscle EvolutionErratum in. Mol. Biol. Evolmol Biol. Evol. 3535, 9141821–9141924. 10.1093/molbev/msy002 PMC5888912 29319812

[B136] Ishibashi J. Perry R. L. Asakura A. Rudnicki M. A. (2005). MyoD Induces Myogenic Differentiation through Cooperation of its NH2- and COOH-Terminal Regions. J. Cel Biol. 171, 471–482. 10.1083/jcb.200502101 PMC2171269 16275751

[B137] Isogai S. Horiguchi M. Weinstein B. M. (2001). The Vascular Anatomy of the Developing Zebrafish: An Atlas of Embryonic and Early Larval Development. Dev. Biol. 230, 278–301. 10.1006/dbio.2000.9995 11161578

[B138] Jackson H. E. Ingham P. W. (2013). Control of Muscle Fibre-type Diversity during Embryonic Development: The Zebrafish Paradigm. Mech. Dev. 130, 447–457. 10.1016/j.mod.2013.06.001 23811405

[B139] Jaffredo T. Lempereur A. Richard C. Bollerot K. Gautier R. Canto P.-Y. (2013). Dorso-ventral Contributions in the Formation of the Embryonic Aorta and the Control of Aortic Hematopoiesis. Blood Cell Mol. Dis. 51, 232–238. 10.1016/j.bcmd.2013.07.004 23932235

[B140] Janesick A. Tang W. Nguyen T. T. L. Blumberg B. (2017). RARβ2 Is Required for Vertebrate Somitogenesis. Development 144, 1997–2008. 10.1242/dev.144345 28432217

[B141] Janesick A. Tang W. Shioda T. Blumberg B. (2018). RARγ Is Required for Mesodermal Gene Expression Prior to Gastrulation. Development 145, 2–9. 10.1242/dev.147769 30111657

[B142] Jayne B. C. Lauder G. V. (1994). How Swimming Fish Use Slow and Fast Muscle Fibers: Implications for Models of Vertebrate Muscle Recruitment. J. Comp. Physiol. A. 175, 123–131. 10.1007/BF00217443 8083846

[B143] Jen W. C. Wettstein D. Turner D. Chitnis A. Kintner C. (1997). The Notch Ligand, X-delta-2, Mediates Segmentation of the Paraxial Mesoderm in Xenopus Embryos. Development 124, 1169–1178. 10.1242/dev.124.6.1169 9102304

[B144] Johnson J. Rhee J. Parsons S. M. Brown D. Olson E. N. Rawls A. (2001). The Anterior/posterior Polarity of Somites Is Disrupted in Paraxis-Deficient Mice. Dev. Biol. 229, 176–187. 10.1006/dbio.2000.9969 11133162

[B145] Jones C. M. Kuehn M. R. Hogan B. L. Smith J. C. Wright C. V. (1995). Nodal-related Signals Induce Axial Mesoderm and Dorsalize Mesoderm during Gastrulation. Development 121, 3651–3662. 10.1242/dev.121.11.3651 8582278

[B146] Joseph E. M. Melton D. A. (1997). Xnr4:AXenopusNodal-Related Gene Expressed in the Spemann Organizer. Dev. Biol. 184, 367–372. 10.1006/dbio.1997.8510 9133442

[B147] Kague E. Hughes S. M. Lawrence E. A. Cross S. Martin‐Silverstone E. Hammond C. L. (2019). Scleraxis Genes Are Required for normal Musculoskeletal Development and for Rib Growth and Mineralization in Zebrafish. FASEB j. 33, 9116–9130. 10.1096/fj.201802654RR 31100023 PMC6662971

[B148] Kahane N. Cinnamon Y. Kalcheim C. (1998a). The Origin and Fate of pioneer Myotomal Cells in the Avian Embryo. Mech. Dev. 74, 59–73. 10.1016/S0925-4773(98)00066-5 9651481

[B149] Kahane N. Cinnamon Y. Kalcheim C. (1998b). The Cellular Mechanism by Which the Dermomyotome Contributes to the Second Wave of Myotome Development. Development 125, 4259–4271. 10.1242/dev.125.21.4259 9753680

[B150] Kahane N. Cinnamon Y. Bachelet I. Kalcheim C. (2001). The Third Wave of Myotome Colonization by Mitotically Competent Progenitors: Regulating the Balance between Differentiation and Proliferation during Muscle Development. Development 128, 2187–2198. 10.1242/dev.128.12.2187 11493539

[B151] Kahane N. Ben-Yair R. Kalcheim C. (2007). Medial pioneer Fibers Pattern the Morphogenesis of Early Myoblasts Derived from the Lateral Somite. Dev. Biol. 305, 439–450. 10.1016/j.ydbio.2007.02.030 17382923

[B152] Kahane N. Ribes V. Kicheva A. Briscoe J. Kalcheim C. (2013). The Transition from Differentiation to Growth during Dermomyotome-Derived Myogenesis Depends on Temporally Restricted Hedgehog Signaling. Development 140, 1740–1750. 10.1242/dev.092726 23533174 PMC3621491

[B153] Kardon G. (1998). Muscle and Tendon Morphogenesis in the Avian Hind Limb. Development 125, 4019–4032. 10.1242/dev.125.20.4019 9735363

[B154] Kassar-Duchossoy L. Gayraud-Morel B. Gomès D. Rocancourt D. Buckingham M. Shinin V. (2004). Mrf4 Determines Skeletal Muscle Identity in Myf5:Myod Double-Mutant Mice. Nature 431, 466–471. 10.1038/nature02876 15386014

[B155] Kato K. Gurdon J. B. (1993). Single-Cell Transplantation Determines the Time when Xenopus Muscle Precursor Cells Acquire a Capacity for Autonomous Differentiation. Proc. Natl. Acad. Sci. 90, 1310–1314. 10.1073/pnas.90.4.1310 8381963 PMC45862

[B156] Kazanskaya O. Glinka A. del Barco Barrantes I. Stannek P. Niehrs C. Wu W. (2004). R-Spondin2 Is a Secreted Activator of Wnt/β-Catenin Signaling and Is Required for Xenopus Myogenesis. Dev. Cel. 7, 525–534. 10.1016/j.devcel.2004.07.019 15469841

[B157] Keenan S. R. Currie P. D. (2019). The Developmental Phases of Zebrafish Myogenesis. J.Dev. Biol. 7, 12. 10.3390/JDB7020012 PMC6632013 31159511

[B158] Keller G. (2000). An Ergodic Theoretic Approach to Mean Field Coupled Maps. Curr. Top. Dev. Biol. 47, 183–208. 10.1007/978-3-0348-8380-1_9 10595306

[B159] Khokha M. K. Yeh J. Grammer T. C. Harland R. M. (2005). Depletion of Three BMP Antagonists from Spemann's Organizer Leads to a Catastrophic Loss of Dorsal Structures. Dev. Cel. 8, 401–411. 10.1016/j.devcel.2005.01.013 15737935

[B160] Kiecker C. Bates T. Bell E. (2016). Molecular Specification of Germ Layers in Vertebrate Embryos. Cell. Mol. Life Sci. 73, 923–947. 10.1007/s00018-015-2092-y 26667903 PMC4744249

[B161] Kiełbówna L. Daczewska M. (2005). The Origin of Syncytial Muscle Fibres in the Myotomes of *Xenopus laevis* - A Revision. Folia Biol. (Krakow) 53, 39–44. 10.3409/1734916054663401 16212106

[B162] Kielbowna L. (1981). The Formation of Somites and Early Myotomal Myogenesis in *Xenopus laevis*, *Bombina variegata* and *Pelobates fuscus* . J. Embryol. Exp. Morphol. 64, 295–304. 7310306

[B163] Kimelman D. (2006). Mesoderm Induction: From Caps to Chips. Nat. Rev. Genet. 7, 360–372. 10.1038/nrg1837 16619051

[B164] Kimmel C. B. Ballard W. W. Kimmel S. R. Ullmann B. Schilling T. F. (1995). Stages of Embryonic Development of the Zebrafish. Dev. Dyn. 203, 253–310. 10.1002/aja.1002030302 8589427

[B165] Kjolby R. A. S. Truchado M. Iruvanti S. Harland R. M. (2019). Integration of Wnt and FGF Signaling in the Xenopus Gastrula at TCF and Ets Binding Sites Shows the Importance of Short Range Repression in Patterning the Marginal Zone. Development 146, dev179580. 10.1242/dev.179580 31285353 PMC6703714

[B166] Kohli V. Schumacher J. A. Desai S. P. Rehn K. Sumanas S. (2013). Arterial and Venous Progenitors of the Major Axial Vessels Originate at Distinct Locations. Dev. Cel. 25, 196–206. 10.1016/j.devcel.2013.03.017 PMC3744361 23639444

[B167] Kolpakova A. Katz S. Keren A. Rojtblat A. Bengal E. (2013). Transcriptional Regulation of Mesoderm Genes by MEF2D during Early Xenopus Development. PLoS One 8, e69693. 10.1371/journal.pone.0069693 23894525 PMC3716644

[B168] Kondo M. (2007). Bone Morphogenetic Proteins in the Early Development of Zebrafish. FEBS J. 274, 2960–2967. 10.1111/j.1742-4658.2007.05838.x 17521339

[B332] Konstantinides N. Averof M. (2014). A Common Cellular Basis For Muscle Regeneration In Arthropods And Vertebrates. Science 343 (6172), 788–791. 10.1126/science.1243529 24385602

[B169] Kremnyov S. Henningfeld K. Viebahn C. Tsikolia N. (2018). Divergent Axial Morphogenesis and Early Shh Expression in Vertebrate Prospective Floor Plate. Evodevo 9, 1–17. 10.1186/s13227-017-0090-x 29423139 PMC5791209

[B170] Krneta-Stankic V. Sabillo A. Domingo C. R. (2010). Temporal and Spatial Patterning of Axial Myotome Fibers in *Xenopus laevis* . Dev. Dyn. 239, 1162–1177. 10.1002/dvdy.22275 20235228 PMC3086394

[B171] Kumano G. Smith W. C. (2000). FGF Signaling Restricts the Primary Blood Islands to Ventral Mesoderm. Dev. Biol. 228, 304–314. 10.1006/dbio.2000.9937 11112331

[B172] Kusakabe R. Kuratani S. (2005). Evolution and Developmental Patterning of the Vertebrate Skeletal Muscles: Perspectives from the Lamprey. Dev. Dyn. 234, 824–834. 10.1002/dvdy.20587 16252276

[B173] Lacalli T. C. Kelly S. J. (1999). Somatic Motoneurones in Amphioxus Larvae: Cell Types, Cell Position and Innervation Patterns. Acta Zool. 80, 113–124. 10.1046/j.1463-6395.1999.80220004.x

[B174] Lagha M. Sato T. Bajard L. Daubas P. Esner M. Montarras D. (2008a). Regulation of Skeletal Muscle Stem Cell Behavior by Pax3 and Pax7. Cold Spring Harbor Symp. Quantit. Biol. 73, 307–315. 10.1101/sqb.2008.73.006 19022756

[B175] Lagha M. Kormish J. D. Rocancourt D. Manceau M. Epstein J. A. Zaret K. S. (2008b). Pax3 Regulation of FGF Signaling Affects the Progression of Embryonic Progenitor Cells into the Myogenic Program. Genes Dev. 22, 1828–1837. 10.1101/gad.477908 18593883 PMC2492669

[B176] Lagha M. Brunelli S. Messina G. Cumano A. Kume T. Relaix F. (2009). Pax3:Foxc2 Reciprocal Repression in the Somite Modulates Muscular versus Vascular Cell Fate Choice in Multipotent Progenitors. Dev. Cel. 17, 892–899. 10.1016/j.devcel.2009.10.021 20059958

[B177] Lauri A. Brunet T. Handberg-Thorsager M. Fischer A. H. L. Simakov O. Steinmetz P. R. H. (2014). Development of the Annelid Axochord: Insights into Notochord Evolution. Science 345, 1365–1368. 10.1126/science.1253396 25214631

[B178] Laurichesse Q. Soler C. (2020). Muscle Development : a View from Adult Myogenesis in Drosophila. Semin. Cel Dev. Biol. 104, 39–50. 10.1016/j.semcdb.2020.02.009 32144008

[B179] Le Guellec D. Morvan-Dubois G. Sire J.-Y. (2004). Skin Development in Bony Fish with Particular Emphasis on Collagen Deposition in the Dermis of the Zebrafish (*Danio rerio*). Int. J. Dev. Biol. 48, 217–231. 10.1387/ijdb.15272388 15272388

[B180] Leéjard V. Brideau G. Blais F. Salingcarnboriboon R. Wagner G. Roehrl M. H. A. (2007). Scleraxis and NFATc Regulate the Expression of the Pro-α1(I) Collagen Gene in Tendon Fibroblasts. J. Biol. Chem. 282, 17665–17675. 10.1074/jbc.M610113200 17430895

[B181] Leal M. A. Fickel S. R. Sabillo A. Ramirez J. Vergara H. M. Nave C. (2014). The Role of Sdf-1α Signaling inXenopus Laevissomite Morphogenesis. Dev. Dyn. 243, 509–526. 10.1002/dvdy.24092 24357195 PMC4040348

[B182] Levine A. J. Munoz-Sanjuan I. Bell E. North A. J. Brivanlou A. H. (2003). Fluorescent Labeling of Endothelial Cells Allows *In Vivo*, Continuous Characterization of the Vascular Development of *Xenopus laevis* . Dev. Biol. 254, 50–67. 10.1016/S0012-1606(02)00029-5 12606281

[B183] Lewandowski D. Dubińska-Magiera M. Migocka-Patrzałek M. Niedbalska-Tarnowska J. Haczkiewicz-Leśniak K. Dzięgiel P. (2020). Everybody Wants to Move-Evolutionary Implications of Trunk Muscle Differentiation in Vertebrate Species. Semin. Cel Dev. Biol. 104, 3–13. 10.1016/j.semcdb.2019.10.009 31759871

[B184] Leyns L. Bouwmeester T. Kim S.-H. Piccolo S. De Robertis E. M. (1997). Frzb-1 Is a Secreted Antagonist of Wnt Signaling Expressed in the Spemann Organizer. Cell 88, 747–756. 10.1016/S0092-8674(00)81921-2 9118218 PMC3061830

[B185] Li H.-Y. Bourdelas A. Carron C. Gomez C. Boucaut J.-C. Shi D.-L. (2006). FGF8, Wnt8 and Myf5 Are Target Genes of Tbx6 during Anteroposterior Specification in Xenopus Embryo. Dev. Biol. 290, 470–481. 10.1016/j.ydbio.2005.11.020 16343478

[B186] Li H.-Y. Bourdelas A. Carron C. Shi D.-L. (2010). The RNA-Binding Protein Seb4/RBM24 Is a Direct Target of MyoD and Is Required for Myogenesis during Xenopus Early Development. Mech. Dev. 127, 281–291. 10.1016/j.mod.2010.03.002 20338237

[B187] Lin Q. Schwarz J. Bucana C. N. Olson E. (1997). Control of Mouse Cardiac Morphogenesis and Myogenesis by Transcription Factor MEF2C. Science 276, 1404–1407. 10.1126/science.276.5317.1404 9162005 PMC4437729

[B188] Lin Q. Lu J. Yanagisawa H. Webb R. Lyons G. E. Richardson J. A. (1998). Requirement of the MADS-Box Transcription Factor MEF2C for Vascular Development. Development 125, 4565–4574. 10.1242/dev.125.22.4565 9778514

[B189] Lin C.-Y. Yung R.-F. Lee H.-C. Chen W.-T. Chen Y.-H. Tsai H.-J. (2006). Myogenic Regulatory Factors Myf5 and Myod Function Distinctly during Craniofacial Myogenesis of Zebrafish. Dev. Biol. 299, 594–608. 10.1016/j.ydbio.2006.08.042 17007832

[B190] Linker C. Lesbros C. Gros J. Burrus L. W. Rawls A. Marcelle C. (2005). β-Catenin-dependent Wnt Signalling Controls the Epithelial Organisation of Somites through the Activation Ofparaxis. Development 132, 3895–3905. 10.1242/dev.01961 16100089

[B191] Lou X. Fang P. Li S. Hu R.-Y. Kuerner K.-M. Steinbeisser H. (2006). Xenopus Tbx6 Mediates Posterior Patterning via Activation of Wnt and FGF Signalling. Cell Res. 16, 771–779. 10.1038/sj.cr.7310093 16953215

[B192] Ma R. C. Jacobs C. T. Sharma P. Kocha K. M. Huang P. (2018). Stereotypic Generation of Axial Tenocytes from Bipartite Sclerotome Domains in Zebrafish. Plos Genet. 14, e1007775–29. 10.1371/journal.pgen.1007775 30388110 PMC6235400

[B193] Maguire R. J. Isaacs H. V. Elizabeth Pownall M. (2012). Early Transcriptional Targets of MyoD Link Myogenesis and Somitogenesis. Dev. Biol. 371, 256–268. 10.1016/j.ydbio.2012.08.027 22954963

[B194] Mankoo B. S. Skuntz S. Harrigan I. Grigorieva E. Candia A. Wright C. V. E. (2003). The Concerted Action of Meox Homeobox Genes Is Required Upstream of Genetic Pathways Essential for the Formation, Patterning and Differentiation of Somites. Development 130, 4655–4664. 10.1242/dev.00687 12925591

[B195] Mansfield J. H. Haller E. Holland N. D. Brent A. E. (2015). Development of Somites and Their Derivatives in Amphioxus, and Implications for the Evolution of Vertebrate Somites. Evodevo 6, 1–30. 10.1186/s13227-015-0007-5 26052418 PMC4458041

[B328] Mariani F. V. Choi G. B. Harland R. M. (2001). The Neural Plate Specifies Somite Size In The Xenopus Laevis Gastrula. Dev Cell. 1 (1), 115–126. 10.1016/s1534-5807(01)00018-1 11703929

[B196] Martin B. L. Harland R. M. (2001). Hypaxial Muscle Migration during Primary Myogenesis in *Xenopus laevis* . Dev. Biol. 239, 270–280. 10.1006/dbio.2001.0434 11784034

[B197] Martin B. L. Kimelman D. (2012). Canonical Wnt Signaling Dynamically Controls Multiple Stem Cell Fate Decisions during Vertebrate Body Formation. Dev. Cel. 22, 223–232. 10.1016/j.devcel.2011.11.001 PMC3465166 22264734

[B198] Martin B. L. Peyrot S. M. Harland R. M. (2007). Hedgehog Signaling Regulates the Amount of Hypaxial Muscle Development during Xenopus Myogenesis. Dev. Biol. 304, 722–734. 10.1016/j.ydbio.2007.01.022 17320852 PMC2080674

[B199] Mayeuf-Louchart A. Lagha M. Danckaert A. Rocancourt D. Relaix F. Vincent S. D. (2014). Notch Regulation of Myogenic versus Endothelial Fates of Cells that Migrate from the Somite to the Limb. Proc. Natl. Acad. Sci. 111, 8844–8849. 10.1073/pnas.1407606111 24927569 PMC4066530

[B200] Mayeuf-Louchart A. Montarras D. Bodin C. Kume T. Vincent S. D. Buckingham M. (2016). Endothelial Cell Specification in the Somite Is Compromised in Pax3-Positive Progenitors of Foxc1/2 Conditional Mutants, with Loss of Forelimb Myogenesis. Development 143, 872–879. 10.1242/dev.128017 26839363 PMC4813335

[B201] Mise T. Iijima M. Inohaya K. Kudo A. Wada H. (2008). Function ofPax1 andPax9 in the Sclerotome of Medaka Fish. Genesis 46, 185–192. 10.1002/dvg.20381 18395830

[B202] Miura S. Davis S. Klingensmith J. Mishina Y. (2006). BMP Signaling in the Epiblast Is Required for Proper Recruitment of the Prospective Paraxial Mesoderm and Development of the Somites. Development 133, 3767–3775. 10.1242/dev.02552 16943278

[B203] Monsoro-Burq A. H. Duprez D. Watanabe Y. Bontoux M. Vincent C. Brickell P. (1996). The Role of Bone Morphogenetic Proteins in Vertebral Development. Development 122, 3607–3616. 10.1242/dev.122.11.3607 8951076

[B204] Mookerjee H. K. (1930). On the Development of the Vertebral Column of Urodela. Philos. Trans. R. Soc. Lond. Ser B 218, 415–446.

[B320] Mookerjee H. K. (1931). On The Development Of The Vertebral Column Of Anura. Philos. Trans. R Soc. London Ser. B 219, 165–196.

[B205] Morin-Kensicki E. M. Eisen J. S. (1997). Sclerotome Development and Peripheral Nervous System Segmentation in Embryonic Zebrafish. Development 124, 159–167. 10.1242/dev.124.1.159 9006077

[B206] Morkel M. Huelsken J. Wakamiya M. Ding J. van de Wetering M. Clevers H. (2003). β-Catenin Regulates Cripto- and Wnt3-dependent Gene Expression Programs in Mouse axis and Mesoderm Formation. Development 130, 6283–6294. 10.1242/dev.00859 14623818

[B207] Morley R. H. Lachani K. Keefe D. Gilchrist M. J. Flicek P. Smith J. C. (2009). A Gene Regulatory Network Directed by Zebrafish No Tail Accounts for its Roles in Mesoderm Formation. Proc. Natl. Acad. Sci. 106, 3829–3834. 10.1073/pnas.0808382106 19225104 PMC2656165

[B208] Murai K. Vernon A. E. Philpott A. Jones P. (2007). Hes6 Is Required for MyoD Induction during Gastrulation. Dev. Biol. 312, 61–76. 10.1016/j.ydbio.2007.09.011 17950722

[B210] Murchison N. D. Price B. A. Conner D. A. Keene D. R. Olson E. N. Tabin C. J. (2007). Regulation of Tendon Differentiation by Scleraxis Distinguishes Force-Transmitting Tendons from Muscle-Anchoring Tendons. Development 134, 2697–2708. 10.1242/dev.001933 17567668

[B211] Nakamura Y. De Paiva Alves E. Veenstra G. J. Hoppler S. (2016). Tissue- and Stage-Specific Wnt Target Gene Expression Is Controlled Subsequent to β-Catenin Recruitment. Development 143, 1914–1925. 10.1242/dev.131664 27068107 PMC4920159

[B212] Nakatani Y. Shingate P. Ravi V. Pillai N. E. Prasad A. McLysaght A. (2021). Reconstruction of Proto-Vertebrate, Proto-Cyclostome and Proto-Gnathostome Genomes Provides New Insights into Early Vertebrate Evolution. Nat. Commun. 12, 4489. 10.1038/s41467-021-24573-z 34301952 PMC8302630

[B213] Neff A. W. Malacinski G. M. Chung H.-M. (1989). Amphibian (Urodele) Myotomes Display Transitory Anterior/posterior and Medial/lateral Differentiation Patterns. Dev. Biol. 132, 529–543. 10.1016/0012-1606(89)90248-0 2647546

[B214] Nentwich O. Dingwell K. S. Nordheim A. Smith J. C. (2009). Downstream of FGF during Mesoderm Formation in Xenopus: The Roles of Elk-1 and Egr-1. Dev. Biol. 336, 313–326. 10.1016/j.ydbio.2009.09.039 19799892

[B215] Neyt C. Jagla K. Thisse C. Thisse B. Haines L. Currie P. D. (2000). Evolutionary Origins of Vertebrate Appendicular Muscle. Nature 408, 82–86. 10.1038/35040549 11081511

[B216] Nguyen P. D. Hollway G. E. Sonntag C. Miles L. B. Hall T. E. Berger S. (2014). Haematopoietic Stem Cell Induction by Somite-Derived Endothelial Cells Controlled by Meox1. Nature 512, 314–318. 10.1038/nature13678 25119043

[B217] Nguyen-Chi M. E. Bryson-Richardson R. Sonntag C. Hall T. E. Gibson A. Sztal T. (2012). Morphogenesis and Cell Fate Determination within the Adaxial Cell Equivalence Group of the Zebrafish Myotome. Plos Genet. 8, e1003014. 10.1371/journal.pgen.1003014 23133395 PMC3486873

[B218] Nichols J. T. Blanco-Sánchez B. Brooks E. P. Parthasarathy R. Dowd J. Subramanian A. (2016). Ligament versus Bone Cell Identity in the Zebrafish Hyoid Skeleton Is Regulated by Mef2ca. Development 143, 4430–4440. 10.1242/dev.141036 27789622 PMC5201047

[B219] Nicolas N. Gallien C.-L. Chanoine C. (1998). Expression of Myogenic Regulatory Factors during Muscle Development ofXenopus: Myogenin mRNA Accumulation Is Limited Strictly to Secondary Myogenesis. Dev. Dyn. 213, 309–321. 10.1002/(sici)1097-0177(199811)213:3<309:aid-aja7>3.0.co;2-z 9825866

[B220] Nimmagadda S. Geetha Loganathan P. Huang R. Scaal M. Schmidt C. Christ B. (2005). BMP4 and Noggin Control Embryonic Blood Vessel Formation by Antagonistic Regulation of VEGFR-2 (Quek1) Expression. Dev. Biol. 280, 100–110. 10.1016/j.ydbio.2005.01.005 15766751

[B221] Nowicki J. L. Takimoto R. Burke A. C. (2003). The Lateral Somitic Frontier: Dorso-Ventral Aspects of Anterio-Posterior Regionalization in Avian Embryos. Mech. Dev. 120, 227–240. 10.1016/S0925-4773(02)00415-X 12559495

[B319] Ohno S. (1970). Evolution by Gene Duplication New York: Springer

[B318] Onai T. (2018). The Evolutionary Origin Of Chordate Segmentation: Revisiting The Enterocoel Theory. Theory Biosci. 137 (1), 1–16. 10.1007/s12064-018-0260-y 29488055

[B222] Osborn D. P. S. Li K. Cutty S. J. Nelson A. C. Wardle F. C. Hinits Y. (2020). Fgf-driven Tbx Protein Activities Directly Induce Myf5 and Myod to Initiate Zebrafish Myogenesis. Development 147, dev184689. 10.1242/DEV.184689 32345657 PMC7197714

[B223] Pardanaud L. Dieterlen-Lièvre F. (1999). Manipulation des potentialités angiopoïétique/hémangiopoïétique chez l'embryon d'oiseau. J. Soc. Biol. 193, 171–179. 10.1051/jbio/1999193020171 10451352

[B224] Pardanaud L. Luton D. Prigent M. Bourcheix L. M. Catala M. Dieterlen-Lièvre F. (1996). Two Distinct Endothelial Lineages in Ontogeny, One of Them Related to Hemopoiesis. Development 122, 1363–1371. 10.1242/dev.122.5.1363 8625825

[B225] Patterson S. E. Bird N. C. Devoto S. H. (2010). BMP Regulation of Myogenesis in Zebrafish. Dev. Dyn. 239, 806–817. 10.1002/dvdy.22243 20151472 PMC2963064

[B226] Polli M. Amaya E. (2002). A Study of Mesoderm Patterning through the Analysis of the Regulation of Xmyf-5 Expression. Development 129, 2917–2927. 10.1242/dev.129.12.2917 12050139

[B227] Potthoff M. J. Olson E. N. (2007). MEF2: A central Regulator of Diverse Developmental Programs. Development 134, 4131–4140. 10.1242/dev.008367 17959722

[B228] Pouget C. Gautier R. Teillet M.-A. Jaffredo T. (2006). Somite-derived Cells Replace Ventral Aortic Hemangioblasts and Provide Aortic Smooth Muscle Cells of the Trunk. Development 133, 1013–1022. 10.1242/dev.02269 16467362

[B229] Pouget C. Pottin K. Jaffredo T. (2008). Sclerotomal Origin of Vascular Smooth Muscle Cells and Pericytes in the Embryo. Dev. Biol. 315, 437–447. 10.1016/j.ydbio.2007.12.045 18255054

[B230] Pourquié O. Coltey M. Bréant C. Le Douarin N. M. (1995). Control of Somite Patterning by Signals from the Lateral Plate. Proc. Natl. Acad. Sci. 92, 3219–3223. 10.1073/pnas.92.8.3219 7724542 PMC42137

[B231] Pourquié O. Fan C.-M. Coltey M. Hirsinger E. Watanabe Y. Bréant C. (1996). Lateral and Axial Signals Involved in Avian Somite Patterning: A Role for BMP4. Cell 84, 461–471. 10.1016/S0092-8674(00)81291-X 8608600

[B232] Prummel K. D. Hess C. Nieuwenhuize S. Parker H. J. Rogers K. W. Kozmikova I. (2019). A Conserved Regulatory Program Initiates Lateral Plate Mesoderm Emergence across Chordates. Nat. Commun. 10, 1–15. 10.1038/s41467-019-11561-7 31451684 PMC6710290

[B233] Radice G. P. Neff A. W. Shim Y. H. Brustis J. J. Malacinski G. M. (1989). Developmental Histories in Amphibian Myogenesis. Int. J. Dev. Biol. 33, 325–343. 2702121

[B234] Radice G. P. (1995). Spatial Expression of Two Tadpole Stage Specific Myosin Heavy Chains in *Xenopus laevis* . Acta Anat. 153, 254–262. 10.1159/000147726 8659249

[B235] Rajan A. M. Ma R. C. Kocha K. M. Zhang D. J. Huang P. (2020). Dual Function of Perivascular Fibroblasts in Vascular Stabilization in Zebrafish. Plos Genet. 16, e1008800. 10.1371/journal.pgen.1008800 33104690 PMC7644104

[B236] Razy-Krajka F. Stolfi A. (2019). Regulation and Evolution of Muscle Development in Tunicates. Evodevo 10, 1–34. 10.1186/s13227-019-0125-6 31249657 PMC6589888

[B237] Relaix F. Rocancourt D. Mansouri A. Buckingham M. (2005). A Pax3/Pax7-dependent Population of Skeletal Muscle Progenitor Cells. Nature 435, 948–953. 10.1038/nature03594 15843801

[B238] Rescan P. Y. Ralliere C. Chauvigné F. Cauty C. (2005). Expression Patterns of Collagen I (α1) Encoding Gene and Muscle-specific Genes Reveal that the Lateral Domain of the Fish Somite Forms a Connective Tissue Surrounding the Myotome. Dev. Dyn. 233, 605–611. 10.1002/dvdy.20337 15768397

[B239] Rhodes S. J. Konieczny S. F. (1989). Identification of MRF4: A New Member of the Muscle Regulatory Factor Gene Family. Genes Dev. 3, 2050–2061. 10.1101/gad.3.12b.2050 2560751

[B240] Row R. H. Pegg A. Kinney B. A. Farr G. H. Maves L. Lowell S. (2018). BMP and FGF Signaling Interact to Pattern Mesoderm by Controlling Basic helix-loop-helix Transcription Factor Activity. Elife 7, 1–27. 10.7554/eLife.31018 PMC6013256 29877796

[B241] Rudnicki M. A. Schnegelsberg P. N. J. Stead R. H. Braun T. Arnold H.-H. Jaenisch R. (1993). MyoD or Myf-5 Is Required for the Formation of Skeletal Muscle. Cell 75, 1351–1359. 10.1016/0092-8674(93)90621-V 8269513

[B242] Ryke P. A. J. (1953). The Ontogenetic Development of the Somatic Musculature of the Trunk of the Aglossal Anuran *Xenopus laevis* (Daudin). Acta Zool. 34, 1–70. 10.1111/j.1463-6395.1953.tb00367.x

[B243] Sánchez R. S. Sánchez S. S. (2013). Characterization Ofpax1,pax9, Anduncxsclerotomal Genes duringXenopus Laevisembryogenesis. Dev. Dyn. 242, 572–579. 10.1002/dvdy.23945 23401059

[B244] Sánchez S. S. Sánchez R. S. (2021). Delineating the Anuran Axial Skeleton. Int. J. Dev. Biol. 65, 177–186. 10.1387/ijdb.200230ss 32930370

[B245] Sabillo A. Ramirez J. Domingo C. R. (2016). Making Muscle: Morphogenetic Movements and Molecular Mechanisms of Myogenesis in *Xenopus laevis* . Semin. Cel Dev. Biol. 51, 80–91. 10.1016/j.semcdb.2016.02.006 PMC4798873 26853935

[B246] Sadahiro T. Isomi M. Muraoka N. Kojima H. Haginiwa S. Kurotsu S. (2018). Tbx6 Induces Nascent Mesoderm from Pluripotent Stem Cells and Temporally Controls Cardiac versus Somite Lineage Diversification. Cel. Stem Cel. 23, 382–395.e5. 10.1016/j.stem.2018.07.001 PMC6190602 30100166

[B247] Sambasivan R. Gayraud-Morel B. Dumas G. Cimper C. Paisant S. Kelly R. G. (2009). Distinct Regulatory Cascades Govern Extraocular and Pharyngeal Arch Muscle Progenitor Cell Fates. Dev. Cel. 16, 810–821. Erratum in: 2009. Dev. Cell, 17,150. 10.1016/j.devcel.2009.05.008 19531352

[B248] Sanchez-Gurmaches J. Guertin D. A. (2014). Adipocytes Arise from Multiple Lineages that Are Heterogeneously and Dynamically Distributed. Nat. Commun. 5, 4099. 10.1038/ncomms5099 24942009 PMC4066194

[B249] Sandmann T. Jensen L. J. Jakobsen J. S. Karzynski M. M. Eichenlaub M. P. Bork P. (2006). A Temporal Map of Transcription Factor Activity: Mef2 Directly Regulates Target Genes at All Stages of Muscle Development. Dev. Cel. 10, 797–807. 10.1016/j.devcel.2006.04.009 16740481

[B250] Sandmann T. Girardot C. Brehme M. Tongprasit W. Stolc V. Furlong E. E. M. (2007). A Core Transcriptional Network for Early Mesoderm Development in *Drosophila melanogaster* . Genes Dev. 21, 436–449. 10.1101/gad.1509007 17322403 PMC1804332

[B251] Sato Y. (2013). Dorsal Aorta Formation: Separate Origins, Lateral-To-Medial Migration, and Remodeling. Develop. Growth Differ. 55, 113–129. 10.1111/dgd.12010 23294360

[B252] Scaal M. Wiegreffe C. (2006). Somite Compartments in Anamniotes. Brain Struct. Funct. 211, 9–19. 10.1007/s00429-006-0127-8 17006657

[B253] Scaal M. (2016). Early Development of the Vertebral Column. Semin. Cel Dev. Biol. 49, 83–91. 10.1016/j.semcdb.2015.11.003 26564689

[B254] Scales J. B. Olson E. N. Perry M. (1991). Differential Expression of Two Distinct Myod Genes in *Xenopus* . Cell Growth Differ. 2, 619–629. 1809374

[B255] Schmidt C. Stoeckelhuber M. McKinnell I. Putz R. Christ B. Patel K. (2004). Wnt 6 Regulates the Epithelialisation Process of the Segmental Plate Mesoderm Leading to Somite Formation. Dev. Biol. 271, 198–209. 10.1016/j.ydbio.2004.03.016 15196961

[B256] Schnapp E. Pistocchi A. S. Karampetsou E. Foglia E. Lamia C. L. Cotelli F. (2009). Induced Early Expression of Mrf4 but Not Myog Rescues Myogenesis in the Myod/myf5 Double-Morphant Zebrafish Embryo. J. Cel Sci. 122, 481–488. 10.1242/jcs.038356 19193870

[B257] Schuster-Gossler K. Cordes R. Gossler A. (2007). Premature Myogenic Differentiation and Depletion of Progenitor Cells Cause Severe Muscle Hypotrophy in Delta1 Mutants. Proc. Natl. Acad. Sci. 104, 537–542. 10.1073/pnas.0608281104 17194759 PMC1766420

[B258] Schweitzer R. Chyung J. H. Murtaugh L. C. Brent A. E. Rosen V. Olson E. N. (2001). Analysis of the Tendon Cell Fate Using Scleraxis, a Specific Marker for Tendons and Ligaments. Development 128, 3855–3866. 10.1242/dev.128.19.3855 11585810

[B259] Sebo Z. L. Jeffery E. Holtrup B. Rodeheffer M. S. (2018). A Mesodermal Fate Map for Adipose Tissue. Development 145, 1–11. 10.1242/dev.166801 PMC6141776 30045918

[B260] Seger C. Hargrave M. Wang X. Chai R. J. Elworthy S. Ingham P. W. (2011). Analysis of Pax7 Expressing Myogenic Cells in Zebrafish Muscle Development, Injury, and Models of Disease. Dev. Dyn. 240, 2440–2451. 10.1002/dvdy.22745 21954137

[B261] Shearman R. M. Burke A. C. (2009). The Lateral Somitic Frontier in Ontogeny and Phylogeny. J. Exp. Zool. 312B, 603–612. 10.1002/jez.b.21246 PMC2962407 19021255

[B262] Shi D.-L. Bourdelas A. Umbhauer M. Boucaut J.-C. (2002). Zygotic Wnt/β-Catenin Signaling Preferentially Regulates the Expression of Myf5 Gene in the Mesoderm of Xenopus. Dev. Biol. 245, 124–135. 10.1006/dbio.2002.0633 11969260

[B334] Shimeld S. M. Holland P. W. (2000). Vertebrate innovations. Proc. Natl. Acad. Sci. USA 97 (9), 4449–4452. 10.1073/pnas.97.9.4449 10781042 PMC34320

[B263] Shukunami C. Takimoto A. Oro M. Hiraki Y. (2006). Scleraxis Positively Regulates the Expression of Tenomodulin, a Differentiation Marker of Tenocytes. Dev. Biol. 298, 234–247. 10.1016/j.ydbio.2006.06.036 16876153

[B264] Shukunami C. Takimoto A. Nishizaki Y. Yoshimoto Y. Tanaka S. Miura S. (2018). Scleraxis Is a Transcriptional Activator that Regulates the Expression of Tenomodulin, a Marker of Mature Tenocytes and Ligamentocytes. Sci. Rep. 8, 1–17. 10.1038/s41598-018-21194-3 29453333 PMC5816641

[B265] Simakov O. Marlétaz F. Yue J.-X. O’Connell B. Jenkins J. Brandt A. (2020). Deeply Conserved Synteny Resolves Early Events in Vertebrate Evolution. Nat. Ecol. Evol. 4, 820–830. 10.1038/s41559-020-1156-z 32313176 PMC7269912

[B266] Sobkow L. Epperlein H.-H. Herklotz S. Straube W. L. Tanaka E. M. (2006). A Germline GFP Transgenic Axolotl and its Use to Track Cell Fate: Dual Origin of the Fin Mesenchyme during Development and the Fate of Blood Cells during Regeneration. Dev. Biol. 290, 386–397. 10.1016/j.ydbio.2005.11.037 16387293

[B267] Somorjai I. M. L. Somorjai R. L. Garcia-Fernandez J. Escriva H. (2012). Vertebrate-like Regeneration in the Invertebrate Chordate Amphioxus. Proc. Natl. Acad. Sci. 109, 517–522. 10.1073/pnas.1100045109 22203957 PMC3258630

[B268] Stafford D. A. Brunet L. J. Khokha M. K. Economides A. N. Harland R. M. (2011). Cooperative Activity of Noggin and Gremlin 1 in Axial Skeleton Development. Development 138, 1005–1014. 10.1242/dev.051938 21303853 PMC3035099

[B269] Standley H. J. Zorn A. M. Gurdon J. B. (2001). eFGF and its Mode of Action in the Community Effect during Xenopus Myogenesis. Development 128, 1347–1357. 10.1242/dev.128.8.1347 11262235

[B270] Steinbacher P. Haslett J. R. Sänger A. M. Stoiber W. (2006). Evolution of Myogenesis in Fish: A sturgeon View of the Mechanisms of Muscle Development. Anat. Embryol. 211, 311–322. 10.1007/s00429-006-0082-4 16506067

[B271] Steinmetz P. R. H. Kraus J. E. M. Larroux C. Hammel J. U. Amon-Hassenzahl A. Houliston E. (2012). Independent Evolution of Striated Muscles in Cnidarians and Bilaterians. Nature 487, 231–234. 10.1038/nature11180 22763458 PMC3398149

[B272] Stellabotte F. Dobbs-McAuliffe B. Fernández D. A. Feng X. Devoto S. H. (2007). Dynamic Somite Cell Rearrangements lead to Distinct Waves of Myotome Growth. Development 134, 1253–1257. 10.1242/dev.000067 17314134

[B273] Stickney H. L. Barresi M. J. F. Devoto S. H. (2000). Somite Development in Zebrafish. Dev. Dyn. 219, 287–303. 10.1002/1097-0177(2000)9999:9999<:AID-DVDY1065>3.0.CO;2-A 11066087

[B274] Stratman A. N. Pezoa S. A. Farrelly O. M. Castranova D. Dye L. E. Butler M. G. (2017). Mural-Endothelial Cell-Cell Interactions Stabilize the Developing Zebrafish Dorsal Aorta. Development 144, 115–127. 10.1242/dev.143131 27913637 PMC5278630

[B275] Tajbakhsh S. Rocancourt D. Cossu G. Buckingham M. (1997). Redefining the Genetic Hierarchies Controlling Skeletal Myogenesis: Pax-3 and Myf-5 Act Upstream of MyoD. Cell 89, 127–138. 10.1016/S0092-8674(00)80189-0 9094721

[B330] Takahashi Y. Takagi A. Hiraoka S. Koseki H. Kanno J. Rawls A. (2007). Transcription Factors Mesp2 And Paraxis Have Critical Roles In Axial Musculoskeletal Formation. Dev. Dyn. 236 (6), 1484–1494. 10.1002/dvdy.21178 17477400

[B276] Tani S. Chung U.-i. Ohba S. Hojo H. (2020). Understanding Paraxial Mesoderm Development and Sclerotome Specification for Skeletal Repair. Exp. Mol. Med. 52, 1166–1177. 10.1038/s12276-020-0482-1 32788657 PMC8080658

[B277] Tarazona O. A. Slota L. A. Lopez D. H. Zhang G. Cohn M. J. (2016). The Genetic Program for Cartilage Development Has Deep Homology within Bilateria. Nature 533, 86–89. 10.1038/nature17398 27111511

[B278] Teillet M. Watanabe Y. Jeffs P. Duprez D. Lapointe F. Le Douarin N. M. (1998). Sonic Hedgehog Is Required for Survival of Both Myogenic and Chondrogenic Somitic Lineages. Development 125, 2019–2030. 10.1242/dev.125.11.2019 9570767

[B279] Teräväinen H. (1971). Anatomical and Physiological Studies on Muscles of Lamprey. J. Neurophysiol. 34, 954–973. 10.1152/jn.1971.34.6.954 5115912

[B280] Thor S. Thomas J. B. (2002). Motor Neuron Specification in Worms, Flies and Mice: Conserved and 'lost' Mechanisms. Curr. Opin. Genet. Dev. 12, 558–564. 10.1016/s0959-437x(02)00340-4 12200161

[B281] Tonegawa A. Funayama N. Ueno N. Takahashi Y. (1997). Mesodermal Subdivision along the Mediolateral axis in Chicken Controlled by Different Concentrations of BMP-4. Development 124, 1975–1984. 10.1242/dev.124.10.1975 9169844

[B282] Venuti J. M. Morris J. H. Vivian J. L. Olson E. N. Klein W. H. (1995). Myogenin Is Required for Late but Not Early Aspects of Myogenesis during Mouse Development. J. Cel Biol. 128, 563–576. 10.1083/jcb.128.4.563 PMC2199898 7532173

[B283] Vergara H. M. Ramirez J. Rosing T. Nave C. Blandino R. Saw D. (2018). miR-206 Is Required for Changes in Cell Adhesion that Drive Muscle Cell Morphogenesis in *Xenopus laevis* . Dev. Biol. 438, 94–110. 10.1016/j.ydbio.2018.03.021 29596841

[B284] Vernon A. E. Philpott A. (2003). A Single Cdk Inhibitor, p27Xic1, Functions beyond Cell Cycle Regulation to Promote Muscle Differentiation inXenopus. Development 130, 71–83. 10.1242/dev.00180 12441292

[B285] Verzi M. P. Agarwal P. Brown C. McCulley D. J. Schwarz J. J. Black B. L. (2007). The Transcription Factor MEF2C Is Required for Craniofacial Development. Dev. Cel. 12, 645–652. 10.1016/j.devcel.2007.03.007 PMC1920108 17420000

[B286] Vogel F. Gemballa S. (2000). Locomotory Design of 'cyclostome' Fishes: Spatial Arrangement and Architecture of Myosepta and Lamellae. Acta Zool. 81, 267–283. 10.1046/j.1463-6395.2000.00056.x

[B287] Wagner J. Schmidt C. Nikowits W. Christ B. (2000). Compartmentalization of the Somite and Myogenesis in Chick Embryos Are Influenced by Wnt Expression. Dev. Biol. 228, 86–94. 10.1006/dbio.2000.9921 11087628

[B288] Walmsley M. Cleaver D. Patient R. (2008). Fibroblast Growth Factor Controls the Timing of Scl, Lmo2, and Runx1 Expression during Embryonic Blood Development. Blood 111, 1157–1166. 10.1182/blood-2007-03-081323 17942750

[B321] Wang G. Jacquet L. Karamariti E. Xu Q. (2015). Origin And Differentiation Of Vascular Smooth Muscle Cells. J. Physiol. 593 (14), 3013–3030. 10.1113/JP270033 25952975 PMC4532522

[B289] Weinberg E. S. Allende M. L. Kelly C. S. Abdelhamid A. Murakami T. Andermann P. (1996). Developmental Regulation of Zebrafish MyoD in Wild-type, No Tail and Spadetail Embryos. Development 122, 271–280. 10.1242/dev.122.1.271 8565839

[B290] Wiegreffe C. Christ B. Huang R. Scaal M. (2007). Sclerotomal Origin of Smooth Muscle Cells in the wall of the Avian Dorsal Aorta. Dev. Dyn. 236, 2578–2585. 10.1002/dvdy.21279 17685486

[B291] Wijgerde M. Karp S. McMahon J. McMahon A. P. (2005). Noggin Antagonism of BMP4 Signaling Controls Development of the Axial Skeleton in the Mouse. Dev. Biol. 286, 149–157. 10.1016/j.ydbio.2005.07.016 16122729

[B324] Williams S. Alkhatib B. Serra R. (2019). Development Of The Axial Skeleton And Intervertebral Disc. Curr. Top Dev. Biol. 133, 49–90. 10.1016/bs.ctdb.2018.11.018 30902259 PMC6800124

[B292] Wilm B. James R. G. Schultheiss T. M. Hogan B. L. M. (2004). The Forkhead Genes, Foxc1 and Foxc2, Regulate Paraxial versus Intermediate Mesoderm Cell Fate. Dev. Biol. 271, 176–189. 10.1016/j.ydbio.2004.03.034 15196959

[B323] Wilson P. A. Oster G. Keller R. (1989). Cell Rearrangement And Segmentation In Xenopus: Direct Observation Of Cultured Explants. Development 105 (1), 155–166. 2806114 10.1242/dev.105.1.155

[B293] Wilson V. Rashbass P. Beddington R. S. (1993). Chimeric Analysis of T (Brachyury) Gene Function. Development 117, 1321–1331. 10.1242/dev.117.4.1321 8404534

[B294] Wilson-Rawls J. Hurt C. R. Parsons S. M. Rawls A. (1999). Differential Regulation of Epaxial and Hypaxial Muscle Development by Paraxis. Development 126, 5217–5229. 10.1242/dev.126.23.5217 10556048

[B295] Wilson-Rawls J. Rhee J. M. Rawls A. (2004). Paraxis Is a Basic helix-loop-helix Protein that Positively Regulates Transcription through Binding to Specific E-Box Elements. J. Biol. Chem. 279, 37685–37692. 10.1074/jbc.M401319200 15226298

[B296] Wilting J. Becker J. (2006). Two Endothelial Cell Lines Derived from the Somite. Brain Struct. Funct. 211, 57–63. 10.1007/s00429-006-0120-2 17047989

[B297] Wilting J. Brand-Saberi B. Huang R. Zhi Q. Köntges G. Ordahl C. P. (1995). Angiogenic Potential of the Avian Somite. Dev. Dyn. 202, 165–171. 10.1002/aja.1002020208 7537553

[B298] Windner S. E. Bird N. C. Patterson S. E. Doris R. A. Devoto S. H. (2012). Fss/Tbx6 Is Required for central Dermomyotome Cell Fate in Zebrafish. Biol. Open 1, 806–814. 10.1242/bio.20121958 23213474 PMC3507223

[B299] Wittenberger T. Steinbach O. C. Authaler A. Kopan R. Rupp R. A. W. (1999). MyoD Stimulates Delta-1 Transcription and Triggers Notch Signaling in the Xenopus Gastrula. EMBO J. 18, 1915–1922. 10.1093/emboj/18.7.1915 10202155 PMC1171277

[B322] Wolff C. Roy S. Ingham P. W. (2003). Multiple Muscle Cell Identities Induced By Distinct Levels And Timing Of Hedgehog Activity In The Zebrafish Embryo. Curr Biol. 15 (14), 1169–1181. 10.1016/s0960-9822(03)00461-5 12867027

[B300] Wotton K. R. Schubert F. R. Dietrich S. (2015). Hypaxial Muscle: Controversial Classification and Controversial Data? Cell Differ. 56, 25–48. 10.1007/978-3-662-44608-9_2 25344665

[B301] Wu W. Folter S. d. Shen X. Zhang W. Tao S. (2011). Vertebrate Paralogous MEF2 Genes: Origin, Conservation, and Evolution. PLoS One 6, e17334. 10.1371/journal.pone.0017334 21394201 PMC3048864

[B302] Wylie C. Kofron M. Payne C. Anderson R. Hosobuchi M. Joseph E. (1996). Maternal Beta-Catenin Establishes a 'dorsal Signal' in Early Xenopus Embryos. Development 122, 2987–2996. 10.1242/dev.122.10.2987 8898213

[B303] Yagi K. Takatori N. Satou Y. Satoh N. (2005). Ci-Tbx6b and Ci-Tbx6c Are Key Mediators of the Maternal Effect Gene Ci-Macho1 in Muscle Cell Differentiation in *Ciona intestinalis* Embryos. Dev. Biol. 282, 535–549. 10.1016/j.ydbio.2005.03.029 15950616

[B304] Yamamoto M. Legendre N. P. Biswas A. A. Lawton A. Yamamoto S. Tajbakhsh S. (2018). Loss of MyoD and Myf5 in Skeletal Muscle Stem Cells Results in Altered Myogenic Programming and Failed Regeneration. Stem Cel Rep. 10, 956–969. 10.1016/j.stemcr.2018.01.027 PMC5918368 29478898

[B305] Yasuoka Y. (2020). Morphogenetic Mechanisms Forming the Notochord Rod: The Turgor Pressure‐sheath Strength Model. Develop. Growth Differ. 62, 379–390. 10.1111/dgd.12665 32275068

[B306] Yasutake J. Inohaya K. Kudo A. (2004). Twist Functions in Vertebral Column Formation in Medaka, *Oryzias latipes* . Mech. Dev. 121, 883–894. 10.1016/j.mod.2004.03.008 15210193

[B307] Yin J. Lee R. Ono Y. Ingham P. W. Saunders T. E. (2018). Spatiotemporal Coordination of FGF and Shh Signaling Underlies the Specification of Myoblasts in the Zebrafish Embryo. Dev. Cel. 46, 735–750.e4. 10.1016/j.devcel.2018.08.024 30253169

[B308] Yong L. W. Lu T.-M. Tung C.-H. Chiou R.-J. Li K.-L. Yu J.-K. (2021). Somite Compartments in Amphioxus and its Implications on the Evolution of the Vertebrate Skeletal Tissues. Front. Cel Dev. Biol. 9, 607057. 10.3389/fcell.2021.607057 PMC8141804 34041233

[B309] Yoon J. K. Wold B. (2000). The bHLH Regulator pMesogenin1 Is Required for Maturation and Segmentation of Paraxial Mesoderm. Genes Dev. 14, 3204–3214. 10.1101/gad.850000 11124811 PMC317151

[B310] Yoshikawa Y. Fujimori T. McMahon A. P. Takada S. (1997). Evidence that Absence ofWnt-3aSignaling Promotes Neuralization Instead of Paraxial Mesoderm Development in the Mouse. Dev. Biol. 183, 234–242. 10.1006/dbio.1997.8502 9126297

[B311] Youn B. W. Malacinski G. M. (1981a). Somitogenesis in the Amphibian *Xenopus laevis*: Scanning Electron Microscopic Analysis of Intrasomitic Cellular Arrangements during Somite Rotation. J. Embryol. Exp. Morphol. 64, 23–43. 10.1242/dev.64.1.23 7310302

[B312] Youn B. W. Malacinski G. M. (1981b). Comparative Analysis of Amphibian Somite Morphogenesis: Cell Rearrangement Patterns during Rosette Formation and Myoblast Fusion. J. Embryol. Exp. Morphol. 66, 1–26. 10.1242/dev.66.1.1 7338706

[B313] Youn B. W. Keller R. E. Malacinski G. M. (1980). An Atlas of Notochord and Somite Morphogenesis in Several Anuran and Urodelean Amphibians. J. Embryol. Exp. Morphol. 59, 223–247. 10.1242/dev.59.1.223 6971322

[B314] Yvernogeau L. Auda-Boucher G. Fontaine-Perus J. (2012). Limb Bud Colonization by Somite-Derived Angioblasts Is a Crucial Step for Myoblast Emigration. Development 139, 277–287. 10.1242/dev.067678 22129828

[B315] Zhong T. P. Childs S. Leu J. P. Fishman M. C. (2001). Gridlock Signalling Pathway Fashions the First Embryonic Artery. Nature 414, 216–220. 10.1038/35102599 11700560

[B316] Zinski J. Tajer B. Mullins M. C. (2018). TGF-β Family Signaling in Early Vertebrate Development. Cold Spring Harb. Perspect. Biol. 10, a033274–76. 10.1101/cshperspect.a033274 28600394 PMC5983195

